# Supplementary material for: Correlated evolution between repertoire size and song plasticity predicts that sexual selection on song promotes open-ended learning
Source: eLife. 2019 Sep 3;8:e44454. doi: 10.7554/eLife.44454 (PMC6721395; doi:10.7554/eLife.44454)
Supplement: Figure 1—source data 1. — There are three plots with associated tables for each song characteristic: The first plot and table set gives the values when the binary categorization of song stability (song-stable and song-plastic) is used. The second plot and table set gives the values when the ternary categorization of early song-stable, delayed song-stable, and song-plastic is used. The final plot and table set gives values when the continuous categorization is used. In the table, ‘Node’ corresponds to the numbered boxes on the tree nodes. ‘State Likelihood’ gives the probability that the common ancestor at a node was in a given leaning state when discrete categorizations are used. S = song stable, p=song plastic, E = early song-stable, D = delayed song-stable. ‘Length of Plasticity’ predicts the value along a continuous spectrum. ‘Trait’ is the predicted value of the song trait being examined in the associated tree for each internal node. [file elife-44454-fig1-data1.pdf]

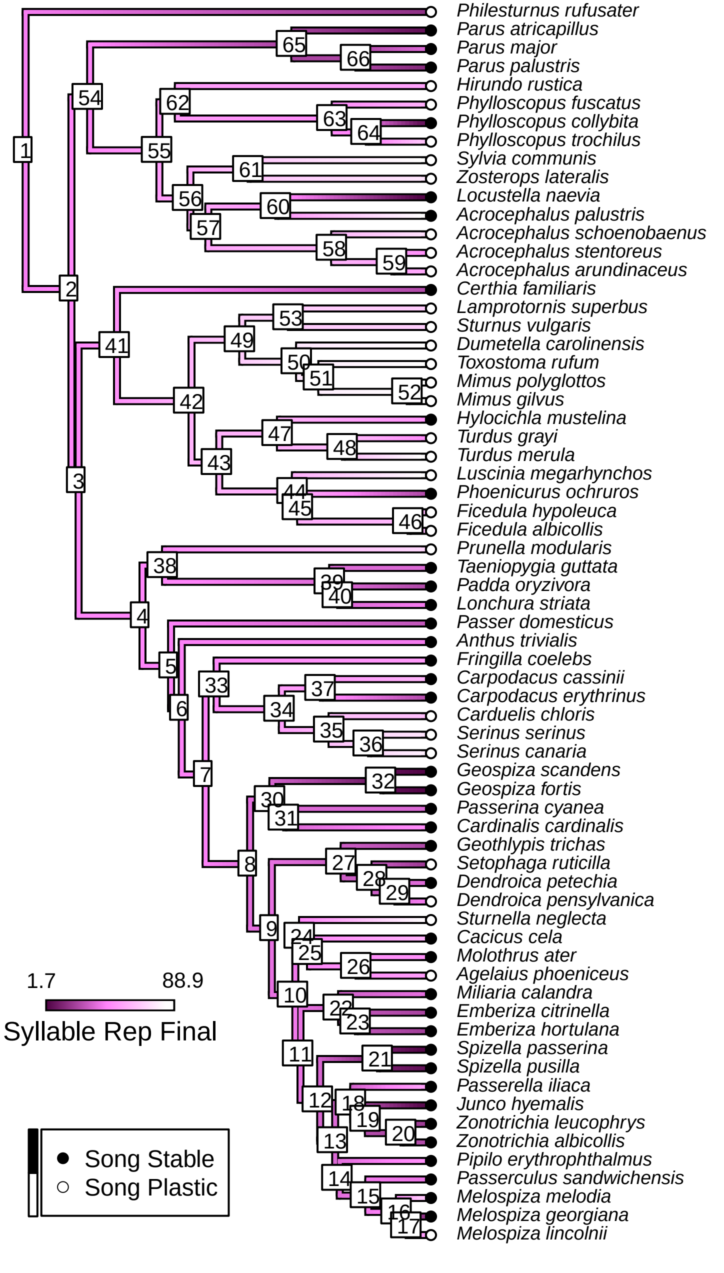

Node	State Likelihood	Trait
1:	S: 0.45, P: 0.55	13.36
2:	S: 0.47, P: 0.53	15.65
3:	S: 0.48, P: 0.52	16.1
4:	S: 0.81, P: 0.19	13.91
5:	S: 0.92, P: 0.08	12.49
6:	S: 0.94, P: 0.06	12.38
7:	S: 0.97, P: 0.03	12.27
8:	S: 1, P: 0	8.85
9:	S: 0.99, P: 0.01	9.08
10:	S: 1, P: 0	10.29
11:	S: 1, P: 0	9.29
12:	S: 1, P: 0	7.19
13:	S: 1, P: 0	7.98
14:	S: 1, P: 0	8.51
15:	S: 1, P: 0	10.44
16:	S: 1, P: 0	13.73
17:	S: 0.97, P: 0.03	9.93
18:	S: 1, P: 0	7.04
19:	S: 1, P: 0	5.11
20:	S: 1, P: 0	4.6
21:	S: 1, P: 0	2.17
22:	S: 1, P: 0	7.05
23:	S: 1, P: 0	5.98
24:	S: 0.98, P: 0.02	12.19
25:	S: 0.98, P: 0.02	13.1
26:	S: 0.88, P: 0.12	15.14
27:	S: 0.85, P: 0.15	6.72
28:	S: 0.52, P: 0.48	6.63
29:	S: 0.52, P: 0.48	8.32
30:	S: 1, P: 0	7.33
31:	S: 0.99, P: 0.01	7.79
32:	S: 1, P: 0	1.74
33:	S: 0.96, P: 0.04	13.25
34:	S: 0.82, P: 0.18	19
35:	S: 0.14, P: 0.86	32.2
36:	S: 0.02, P: 0.98	48.74
37:	S: 0.93, P: 0.07	15.43
38:	S: 0.79, P: 0.21	14.38
39:	S: 0.99, P: 0.01	8.44
40:	S: 0.99, P: 0.01	8.26
41:	S: 0.4, P: 0.6	20.44
42:	S: 0.1, P: 0.9	45.77
43:	S: 0.1, P: 0.9	43.71
44:	S: 0.1, P: 0.9	52.08
45:	S: 0.11, P: 0.89	46.34
46:	S: 0, P: 1	30.57
47:	S: 0.15, P: 0.85	34.35
48:	S: 0.03, P: 0.97	37.52
49:	S: 0.03, P: 0.97	86.17
50:	S: 0.01, P: 0.99	254.69
51:	S: 0.01, P: 0.99	371.72
52:	S: 0, P: 1	331.49
53:	S: 0.02, P: 0.98	62.54
54:	S: 0.45, P: 0.55	15.51
55:	S: 0.21, P: 0.79	22.65
56:	S: 0.19, P: 0.81	29.27
57:	S: 0.22, P: 0.78	27.84
58:	S: 0.03, P: 0.97	29.67
59:	S: 0, P: 1	20.15
60:	S: 0.68, P: 0.32	22.07
61:	S: 0.12, P: 0.88	57.11
62:	S: 0.19, P: 0.81	21.5
63:	S: 0.13, P: 0.87	14.44
64:	S: 0.18, P: 0.82	10.66
65:	S: 0.93, P: 0.07	4.62
66:	S: 0.99, P: 0.01	4.78

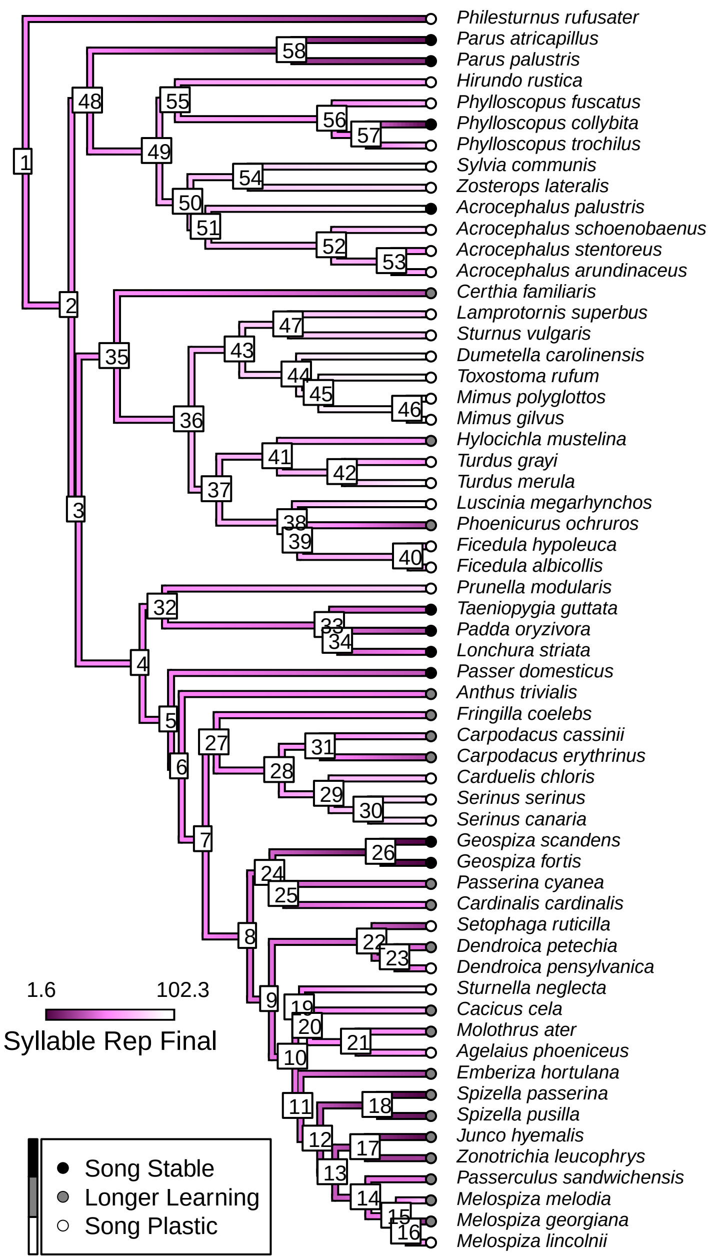

Node	State Likelihood	Trait
1:	E: 0.08, D: 0.1, P: 0.82	14.24
2:	E: 0.06, D: 0.08, P: 0.86	16.8
3:	E: 0.06, D: 0.09, P: 0.85	17.15
4:	E: 0.15, D: 0.34, P: 0.5	14.26
5:	E: 0.12, D: 0.65, P: 0.23	12.66
6:	E: 0.06, D: 0.8, P: 0.14	12.5
7:	E: 0.01, D: 0.92, P: 0.06	12.3
8:	E: 0.01, D: 0.98, P: 0.02	8.75
9:	E: 0, D: 0.97, P: 0.02	8.89
10:	E: 0, D: 0.99, P: 0.01	9.58
11:	E: 0, D: 1, P: 0	8.47
12:	E: 0, D: 1, P: 0	5.96
13:	E: 0, D: 1, P: 0	5.94
14:	E: 0, D: 1, P: 0	9.12
15:	E: 0, D: 1, P: 0	13.28
16:	E: 0, D: 0.99, P: 0.01	9.79
17:	E: 0, D: 1, P: 0	3.86
18:	E: 0, D: 1, P: 0	2.07
19:	E: 0, D: 0.98, P: 0.02	11.47
20:	E: 0, D: 0.98, P: 0.02	12.43
21:	E: 0.01, D: 0.92, P: 0.08	14.82
22:	E: 0, D: 0.31, P: 0.69	6.9
23:	E: 0, D: 0.32, P: 0.68	8.44
24:	E: 0.02, D: 0.97, P: 0.01	7.27
25:	E: 0.01, D: 0.98, P: 0.01	7.74
26:	E: 0.98, D: 0.02, P: 0	1.73
27:	E: 0.01, D: 0.92, P: 0.07	13.28
28:	E: 0.01, D: 0.85, P: 0.14	19.02
29:	E: 0.01, D: 0.08, P: 0.91	32.21
30:	E: 0, D: 0.01, P: 0.99	48.75
31:	E: 0.01, D: 0.96, P: 0.04	15.43
32:	E: 0.21, D: 0.29, P: 0.5	14.68
33:	E: 0.99, D: 0.01, P: 0.01	8.47
34:	E: 0.99, D: 0, P: 0	8.29
35:	E: 0.04, D: 0.09, P: 0.87	21.35
36:	E: 0, D: 0.01, P: 0.98	46.55
37:	E: 0, D: 0.02, P: 0.98	44.22
38:	E: 0, D: 0.03, P: 0.97	52.3
39:	E: 0, D: 0.03, P: 0.96	46.52
40:	E: 0, D: 0, P: 1	30.58
41:	E: 0.01, D: 0.05, P: 0.94	34.55
42:	E: 0, D: 0.01, P: 0.99	37.6
43:	E: 0, D: 0, P: 1	86.93
44:	E: 0, D: 0, P: 1	255.68
45:	E: 0, D: 0, P: 1	372.72
46:	E: 0, D: 0, P: 1	331.55
47:	E: 0, D: 0, P: 0.99	62.86
48:	E: 0.07, D: 0.06, P: 0.87	17.05
49:	E: 0.01, D: 0.01, P: 0.98	29.67
50:	E: 0.01, D: 0, P: 0.98	44.33
51:	E: 0.03, D: 0.01, P: 0.96	48.22
52:	E: 0, D: 0, P: 0.99	33.92
53:	E: 0, D: 0, P: 1	20.68
54:	E: 0.01, D: 0.01, P: 0.98	72.9
55:	E: 0.01, D: 0.01, P: 0.98	27.08
56:	E: 0.03, D: 0, P: 0.96	15.08
57:	E: 0.07, D: 0.01, P: 0.92	10.87
58:	E: 0.88, D: 0.03, P: 0.09	3.9

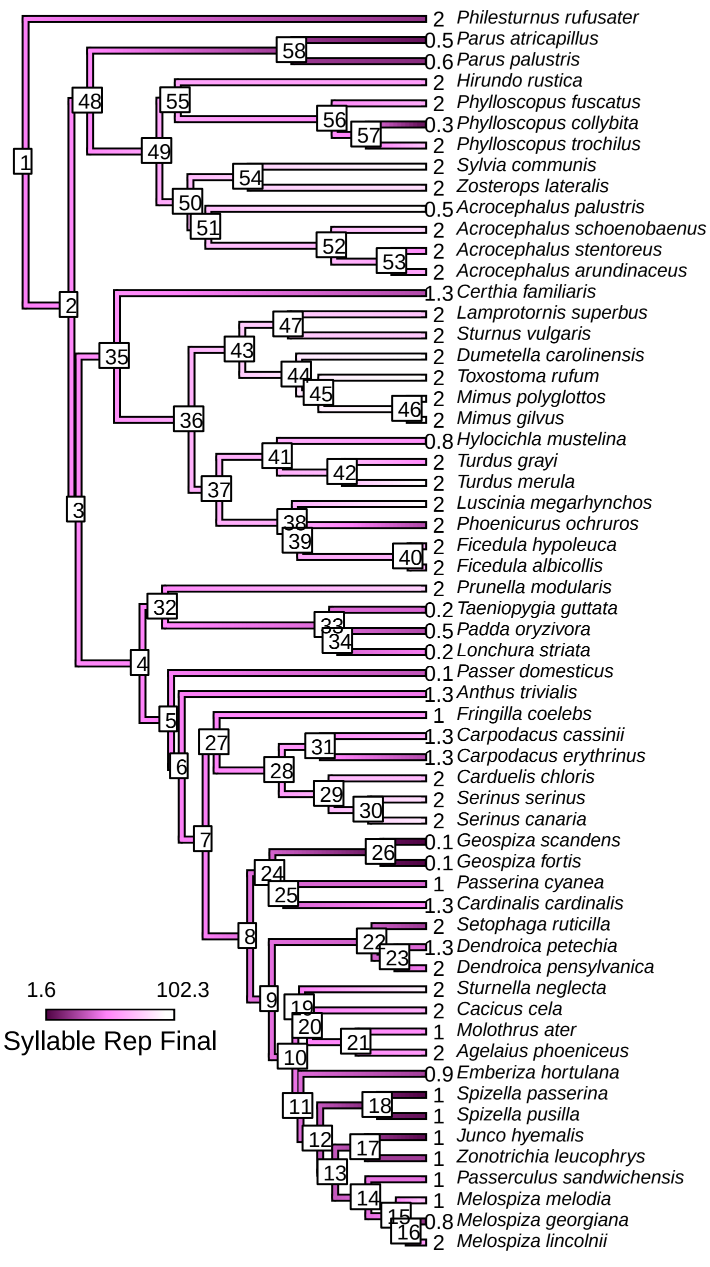

Node	Length of Plasticity	Trait
1:	1.46	14.24
2:	1.4	16.8
3:	1.4	17.15
4:	1.21	14.26
5:	1.16	12.66
6:	1.18	12.5
7:	1.23	12.3
8:	1.2	8.75
9:	1.31	8.89
10:	1.35	9.58
11:	1.3	8.47
12:	1.2	5.96
13:	1.16	5.94
14:	1.15	9.12
15:	1.22	13.28
16:	1.34	9.79
17:	1.07	3.86
18:	1.05	2.07
19:	1.42	11.47
20:	1.46	12.43
21:	1.48	14.82
22:	1.72	6.9
23:	1.69	8.44
24:	1.06	7.27
25:	1.08	7.74
26:	0.26	1.73
27:	1.26	13.28
28:	1.5	19.02
29:	1.78	32.21
30:	1.91	48.75
31:	1.42	15.43
32:	1.18	14.68
33:	0.44	8.47
34:	0.43	8.29
35:	1.49	21.35
36:	1.72	46.55
37:	1.73	44.22
38:	1.9	52.3
39:	1.91	46.52
40:	2	30.58
41:	1.62	34.55
42:	1.86	37.6
43:	1.86	86.93
44:	1.94	255.68
45:	1.96	372.72
46:	2	331.55
47:	1.92	62.86
48:	1.39	17.05
49:	1.54	29.67
50:	1.57	44.33
51:	1.53	48.22
52:	1.89	33.92
53:	1.98	20.68
54:	1.75	72.9
55:	1.57	27.08
56:	1.5	15.08
57:	1.3	10.87
58:	0.74	3.9

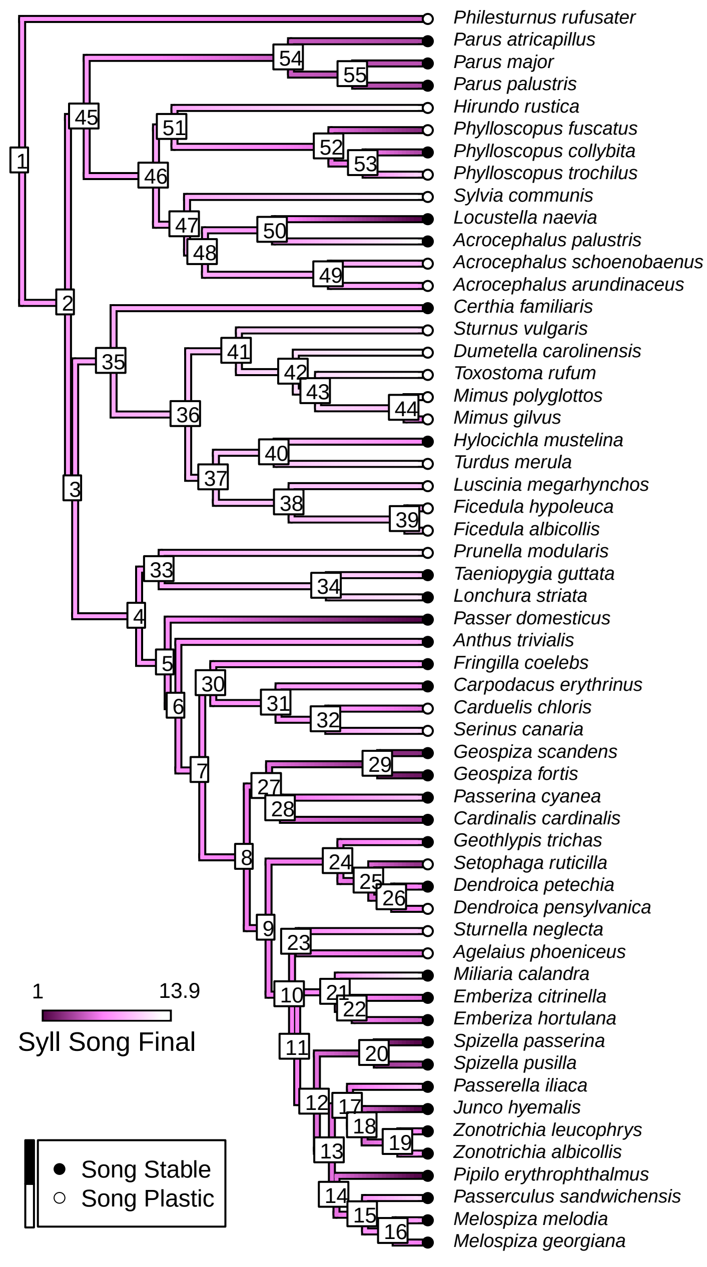

Node	State Likelihood	Trait
1:	S: 0.48, P: 0.52	5.49
2:	S: 0.51, P: 0.49	6.01
3:	S: 0.52, P: 0.48	6.17
4:	S: 0.81, P: 0.19	5.41
5:	S: 0.91, P: 0.09	4.47
6:	S: 0.93, P: 0.07	4.43
7:	S: 0.95, P: 0.05	4.23
8:	S: 0.99, P: 0.01	3.51
9:	S: 0.98, P: 0.02	3.58
10:	S: 0.97, P: 0.03	3.72
11:	S: 0.99, P: 0.01	3.67
12:	S: 1, P: 0	3.08
13:	S: 1, P: 0	3.11
14:	S: 1, P: 0	3.1
15:	S: 1, P: 0	4.43
16:	S: 1, P: 0	4.25
17:	S: 1, P: 0	3.21
18:	S: 1, P: 0	2.8
19:	S: 1, P: 0	3.88
20:	S: 1, P: 0	1.74
21:	S: 1, P: 0	4.75
22:	S: 1, P: 0	3.94
23:	S: 0.81, P: 0.19	3.82
24:	S: 0.84, P: 0.16	3.38
25:	S: 0.51, P: 0.49	2.86
26:	S: 0.51, P: 0.49	3.31
27:	S: 0.99, P: 0.01	3.15
28:	S: 0.99, P: 0.01	3.22
29:	S: 1, P: 0	1.53
30:	S: 0.93, P: 0.07	4.33
31:	S: 0.72, P: 0.28	4.75
32:	S: 0.2, P: 0.8	5.05
33:	S: 0.79, P: 0.21	6
34:	S: 0.96, P: 0.04	8.27
35:	S: 0.43, P: 0.57	7.72
36:	S: 0.11, P: 0.89	13.24
37:	S: 0.1, P: 0.9	11.13
38:	S: 0.04, P: 0.96	8.88
39:	S: 0, P: 1	7.32
40:	S: 0.2, P: 0.8	9.12
41:	S: 0.05, P: 0.95	26.26
42:	S: 0.01, P: 0.99	77.63
43:	S: 0.01, P: 0.99	84.94
44:	S: 0, P: 1	25.94
45:	S: 0.5, P: 0.5	5.81
46:	S: 0.29, P: 0.71	6.79
47:	S: 0.29, P: 0.71	7.46
48:	S: 0.32, P: 0.68	7.46
49:	S: 0.05, P: 0.95	6.06
50:	S: 0.72, P: 0.28	8.4
51:	S: 0.26, P: 0.74	6.69
52:	S: 0.15, P: 0.85	3.27
53:	S: 0.2, P: 0.8	3.78
54:	S: 0.94, P: 0.06	2.56
55:	S: 0.99, P: 0.01	2.24

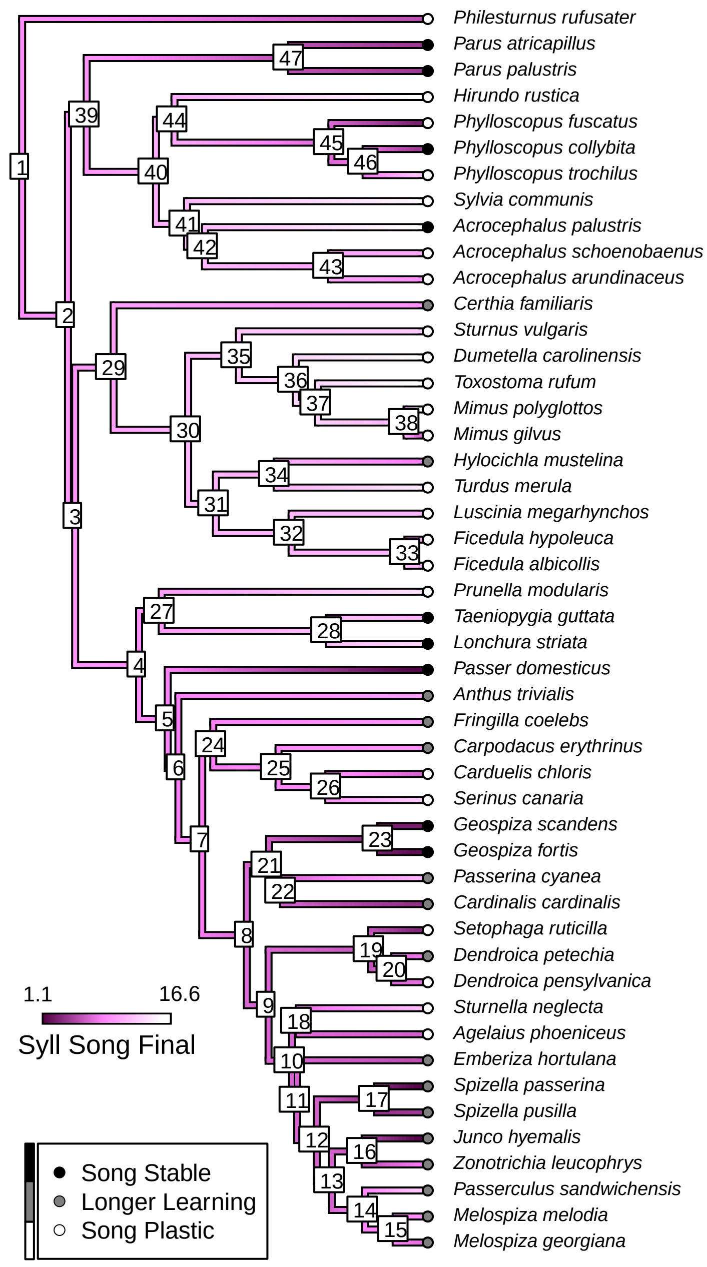

Node	State Likelihood	Trait
1:	E: 0.09, D: 0.1, P: 0.82	5.88
2:	E: 0.06, D: 0.07, P: 0.86	6.49
3:	E: 0.06, D: 0.08, P: 0.86	6.61
4:	E: 0.14, D: 0.28, P: 0.58	5.5
5:	E: 0.11, D: 0.51, P: 0.37	4.45
6:	E: 0.06, D: 0.63, P: 0.3	4.38
7:	E: 0.02, D: 0.73, P: 0.25	4.11
8:	E: 0.01, D: 0.8, P: 0.18	3.26
9:	E: 0.01, D: 0.77, P: 0.22	3.17
10:	E: 0, D: 0.79, P: 0.21	3.2
11:	E: 0, D: 0.91, P: 0.09	3.11
12:	E: 0, D: 0.99, P: 0.01	2.86
13:	E: 0, D: 1, P: 0	3.07
14:	E: 0, D: 1, P: 0	4.52
15:	E: 0, D: 1, P: 0	4.28
16:	E: 0, D: 1, P: 0	2.43
17:	E: 0, D: 1, P: 0	1.7
18:	E: 0.01, D: 0.61, P: 0.39	3.34
19:	E: 0, D: 0.25, P: 0.75	2.65
20:	E: 0, D: 0.26, P: 0.74	3.22
21:	E: 0.04, D: 0.85, P: 0.11	2.99
22:	E: 0.03, D: 0.9, P: 0.07	3.08
23:	E: 0.98, D: 0.02, P: 0	1.51
24:	E: 0.02, D: 0.73, P: 0.25	4.22
25:	E: 0.02, D: 0.6, P: 0.37	4.7
26:	E: 0.01, D: 0.12, P: 0.87	5.02
27:	E: 0.19, D: 0.24, P: 0.57	6.08
28:	E: 0.93, D: 0.03, P: 0.04	8.29
29:	E: 0.05, D: 0.08, P: 0.87	8.09
30:	E: 0, D: 0.01, P: 0.98	13.5
31:	E: 0, D: 0.02, P: 0.98	11.28
32:	E: 0, D: 0.01, P: 0.99	8.93
33:	E: 0, D: 0, P: 1	7.32
34:	E: 0.02, D: 0.09, P: 0.89	9.19
35:	E: 0, D: 0.01, P: 0.99	26.55
36:	E: 0, D: 0, P: 1	78.02
37:	E: 0, D: 0, P: 1	85.23
38:	E: 0, D: 0, P: 1	25.94
39:	E: 0.07, D: 0.06, P: 0.87	6.46
40:	E: 0.03, D: 0.01, P: 0.96	8.59
41:	E: 0.03, D: 0.01, P: 0.96	10.59
42:	E: 0.05, D: 0.01, P: 0.94	11.64
43:	E: 0.01, D: 0, P: 0.99	6.81
44:	E: 0.03, D: 0.01, P: 0.96	8.18
45:	E: 0.04, D: 0.01, P: 0.96	3.4
46:	E: 0.08, D: 0.01, P: 0.91	3.84
47:	E: 0.88, D: 0.03, P: 0.09	2.65

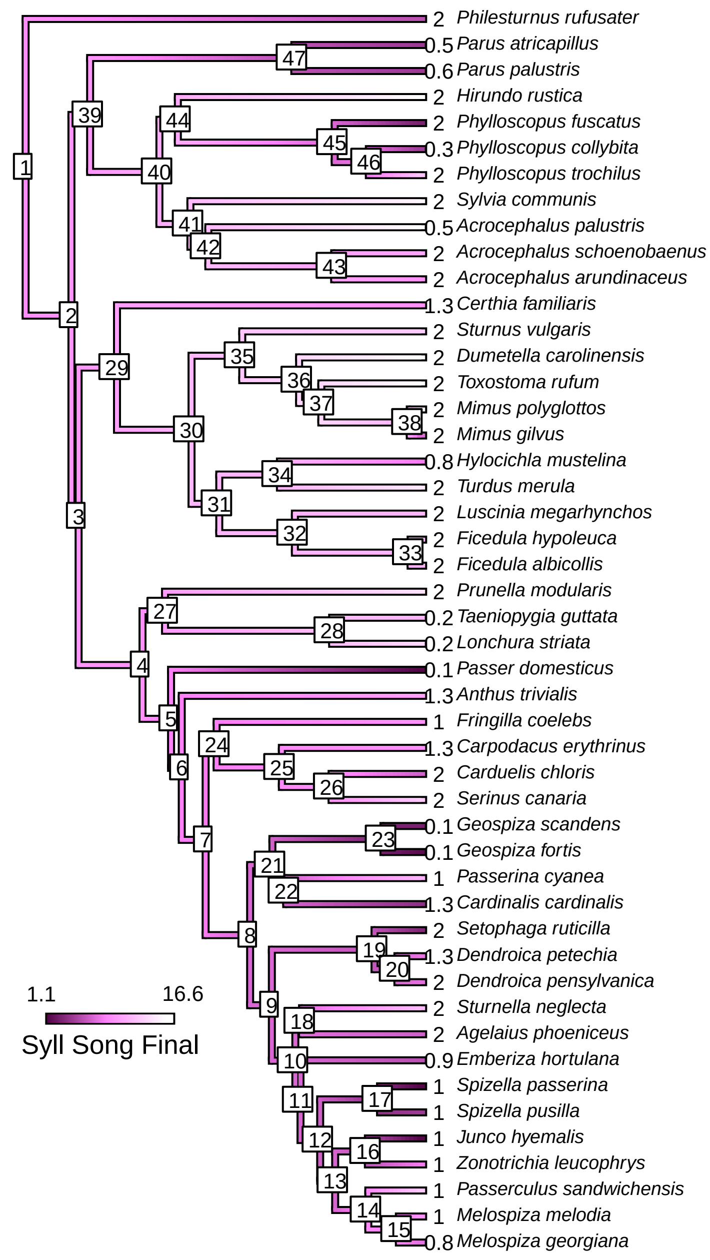

Node	Length of Plasticity	Trait
1:	1.44	5.88
2:	1.38	6.49
3:	1.38	6.61
4:	1.19	5.5
5:	1.15	4.45
6:	1.17	4.38
7:	1.22	4.11
8:	1.19	3.26
9:	1.3	3.17
10:	1.32	3.2
11:	1.27	3.11
12:	1.16	2.86
13:	1.1	3.07
14:	1.01	4.52
15:	0.95	4.28
16:	1.04	2.43
17:	1.04	1.7
18:	1.39	3.34
19:	1.72	2.65
20:	1.69	3.22
21:	1.06	2.99
22:	1.07	3.08
23:	0.26	1.51
24:	1.25	4.22
25:	1.51	4.7
26:	1.76	5.02
27:	1.16	6.08
28:	0.41	8.29
29:	1.47	8.09
30:	1.68	13.5
31:	1.69	11.28
32:	1.86	8.93
33:	1.99	7.32
34:	1.56	9.19
35:	1.82	26.55
36:	1.92	78.02
37:	1.95	85.23
38:	2	25.94
39:	1.36	6.46
40:	1.51	8.59
41:	1.52	10.59
42:	1.49	11.64
43:	1.87	6.81
44:	1.54	8.18
45:	1.5	3.4
46:	1.3	3.84
47:	0.74	2.65

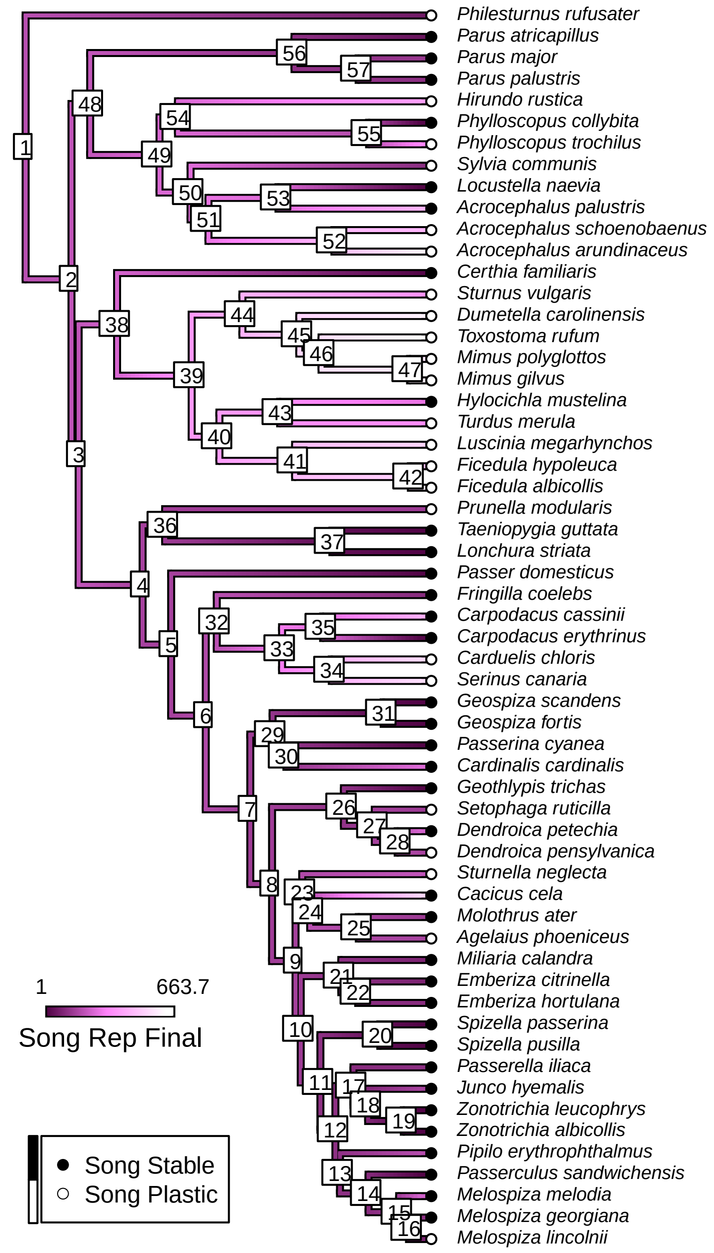

Node	State Likelihood	Trait
1:	S: 0.54, P: 0.46	6.99
2:	S: 0.58, P: 0.42	8.17
3:	S: 0.58, P: 0.42	8.3
4:	S: 0.8, P: 0.2	5.01
5:	S: 0.88, P: 0.12	4.9
6:	S: 0.94, P: 0.06	5.92
7:	S: 0.99, P: 0.01	4.21
8:	S: 0.99, P: 0.01	4.3
9:	S: 1, P: 0	4.81
10:	S: 1, P: 0	4.25
11:	S: 1, P: 0	3.13
12:	S: 1, P: 0	3.06
13:	S: 1, P: 0	3.12
14:	S: 1, P: 0	2.74
15:	S: 0.99, P: 0.01	4.2
16:	S: 0.97, P: 0.03	3.15
17:	S: 1, P: 0	2.75
18:	S: 1, P: 0	2.47
19:	S: 1, P: 0	1.22
20:	S: 1, P: 0	1.35
21:	S: 1, P: 0	3.07
22:	S: 1, P: 0	2.85
23:	S: 0.98, P: 0.02	5.82
24:	S: 0.97, P: 0.03	7.14
25:	S: 0.86, P: 0.14	5.39
26:	S: 0.83, P: 0.17	3.29
27:	S: 0.51, P: 0.49	4.64
28:	S: 0.51, P: 0.49	6.44
29:	S: 0.99, P: 0.01	3.47
30:	S: 0.99, P: 0.01	3.51
31:	S: 1, P: 0	1.21
32:	S: 0.93, P: 0.07	6.85
33:	S: 0.81, P: 0.19	21.56
34:	S: 0.25, P: 0.75	81.91
35:	S: 0.92, P: 0.08	14.75
36:	S: 0.78, P: 0.22	4.27
37:	S: 0.95, P: 0.05	1.36
38:	S: 0.48, P: 0.52	12.22
39:	S: 0.15, P: 0.85	47.66
40:	S: 0.14, P: 0.86	53.81
41:	S: 0.06, P: 0.94	116.85
42:	S: 0, P: 1	216.29
43:	S: 0.24, P: 0.76	37.67
44:	S: 0.07, P: 0.93	96.35
45:	S: 0.02, P: 0.98	279.23
46:	S: 0.01, P: 0.99	381.25
47:	S: 0, P: 1	412.84
48:	S: 0.57, P: 0.43	8.36
49:	S: 0.43, P: 0.57	12.75
50:	S: 0.41, P: 0.59	14.5
51:	S: 0.43, P: 0.57	17.64
52:	S: 0.07, P: 0.93	115.21
53:	S: 0.74, P: 0.26	13.35
54:	S: 0.41, P: 0.59	13.21
55:	S: 0.47, P: 0.53	6.2
56:	S: 0.93, P: 0.07	3.11
57:	S: 0.98, P: 0.02	3.27

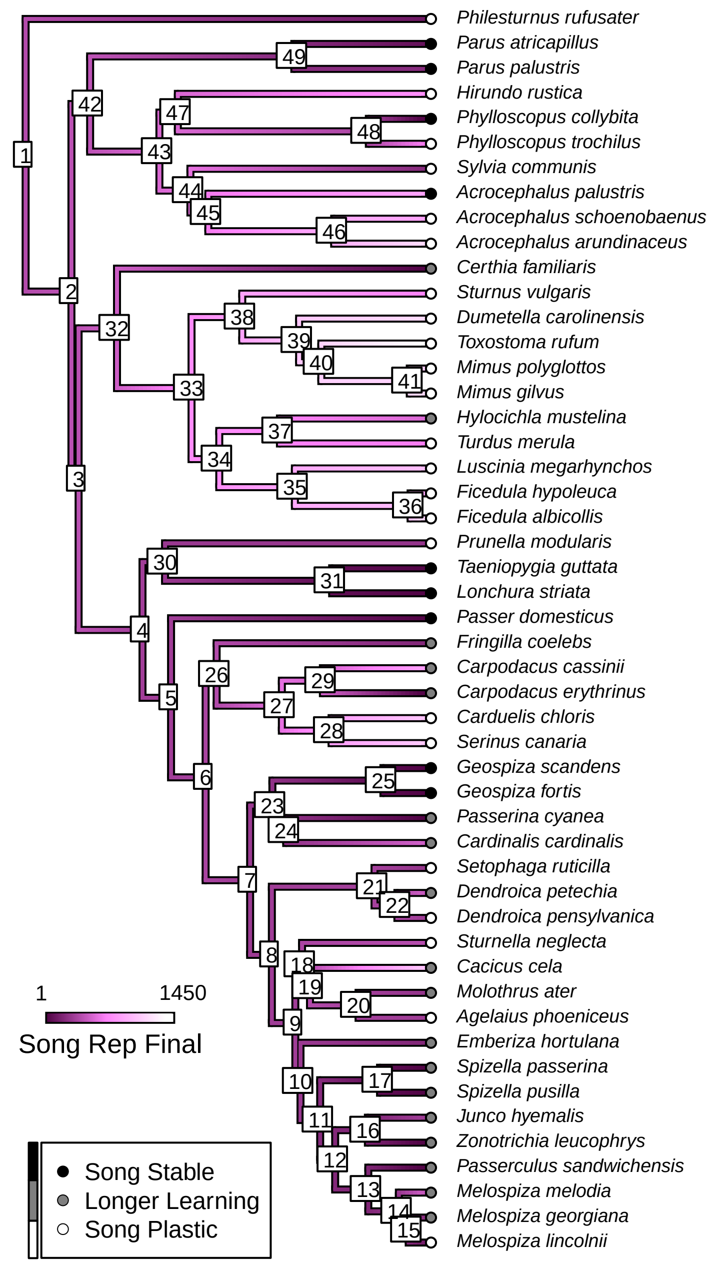

Node	State Likelihood	Trait
1:	E: 0.14, D: 0.12, P: 0.73	7.68
2:	E: 0.12, D: 0.1, P: 0.78	9.08
3:	E: 0.12, D: 0.11, P: 0.77	9.14
4:	E: 0.21, D: 0.29, P: 0.5	5.33
5:	E: 0.19, D: 0.49, P: 0.32	5.19
6:	E: 0.04, D: 0.83, P: 0.13	6.31
7:	E: 0.01, D: 0.94, P: 0.04	4.66
8:	E: 0.01, D: 0.95, P: 0.04	5
9:	E: 0, D: 0.99, P: 0.01	5.25
10:	E: 0, D: 0.99, P: 0.01	4.62
11:	E: 0, D: 1, P: 0	3.11
12:	E: 0, D: 1, P: 0	2.83
13:	E: 0, D: 1, P: 0	2.61
14:	E: 0, D: 1, P: 0	4.15
15:	E: 0, D: 0.98, P: 0.02	3.13
16:	E: 0, D: 1, P: 0	2.49
17:	E: 0, D: 1, P: 0	1.35
18:	E: 0, D: 0.98, P: 0.02	6.27
19:	E: 0, D: 0.97, P: 0.02	7.61
20:	E: 0.01, D: 0.9, P: 0.09	5.53
21:	E: 0.01, D: 0.3, P: 0.7	5.7
22:	E: 0, D: 0.31, P: 0.68	6.94
23:	E: 0.03, D: 0.94, P: 0.03	3.72
24:	E: 0.02, D: 0.96, P: 0.02	3.72
25:	E: 0.98, D: 0.02, P: 0	1.22
26:	E: 0.03, D: 0.84, P: 0.13	7.23
27:	E: 0.02, D: 0.78, P: 0.2	22.06
28:	E: 0.02, D: 0.17, P: 0.81	82.81
29:	E: 0.01, D: 0.93, P: 0.06	14.93
30:	E: 0.25, D: 0.25, P: 0.49	4.5
31:	E: 0.93, D: 0.03, P: 0.04	1.38
32:	E: 0.09, D: 0.11, P: 0.81	13.08
33:	E: 0.01, D: 0.02, P: 0.97	49
34:	E: 0.01, D: 0.03, P: 0.97	54.88
35:	E: 0.01, D: 0.01, P: 0.98	117.9
36:	E: 0, D: 0, P: 1	216.38
37:	E: 0.03, D: 0.11, P: 0.87	38.08
38:	E: 0.01, D: 0.01, P: 0.99	97.87
39:	E: 0, D: 0, P: 1	281.17
40:	E: 0, D: 0, P: 1	383.07
41:	E: 0, D: 0, P: 1	412.98
42:	E: 0.14, D: 0.08, P: 0.78	9.56
43:	E: 0.09, D: 0.03, P: 0.88	17.08
44:	E: 0.09, D: 0.02, P: 0.89	22.33
45:	E: 0.1, D: 0.03, P: 0.87	30.41
46:	E: 0.01, D: 0.01, P: 0.98	132.82
47:	E: 0.1, D: 0.03, P: 0.87	17
48:	E: 0.29, D: 0.02, P: 0.68	6.4
49:	E: 0.86, D: 0.04, P: 0.09	3.04

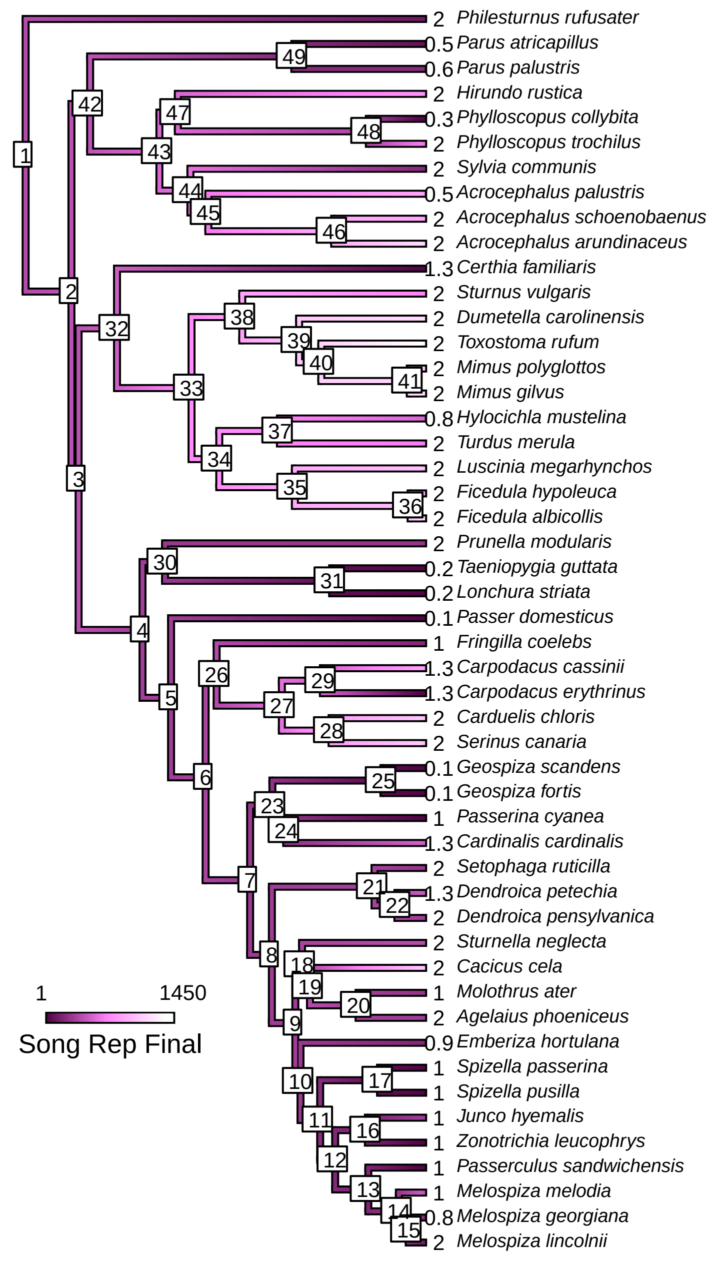

Node	Length of Plasticity	Trait
1:	1.42	7.68
2:	1.35	9.08
3:	1.35	9.14
4:	1.17	5.33
5:	1.12	5.19
6:	1.2	6.31
7:	1.19	4.66
8:	1.31	5
9:	1.34	5.25
10:	1.3	4.62
11:	1.2	3.11
12:	1.16	2.83
13:	1.15	2.61
14:	1.22	4.15
15:	1.34	3.13
16:	1.07	2.49
17:	1.05	1.35
18:	1.41	6.27
19:	1.46	7.61
20:	1.48	5.53
21:	1.72	5.7
22:	1.69	6.94
23:	1.06	3.72
24:	1.07	3.72
25:	0.26	1.22
26:	1.23	7.23
27:	1.48	22.06
28:	1.75	82.81
29:	1.41	14.93
30:	1.14	4.5
31:	0.41	1.38
32:	1.45	13.08
33:	1.68	49
34:	1.68	54.88
35:	1.86	117.9
36:	1.99	216.38
37:	1.56	38.08
38:	1.82	97.87
39:	1.92	281.17
40:	1.94	383.07
41:	2	412.98
42:	1.33	9.56
43:	1.45	17.08
44:	1.48	22.33
45:	1.46	30.41
46:	1.86	132.82
47:	1.46	17
48:	1.18	6.4
49:	0.73	3.04

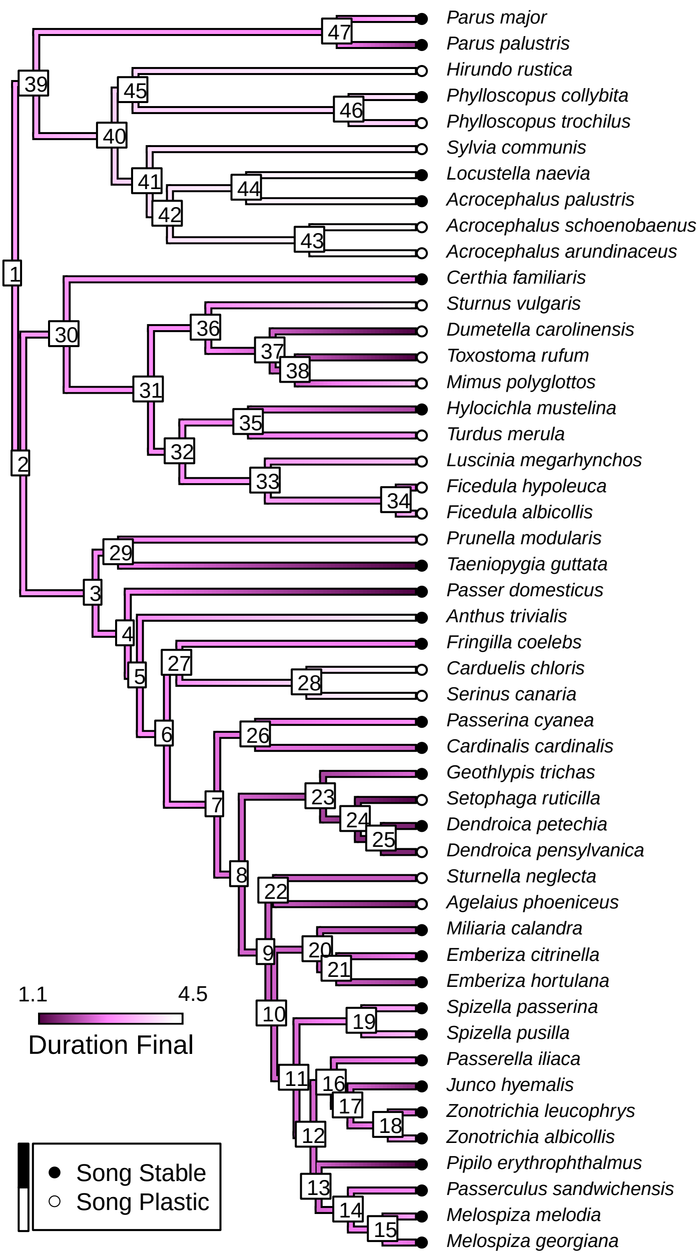

Node	State Likelihood	Trait
1:	S: 0.54, P: 0.46	3.03
2:	S: 0.55, P: 0.45	2.94
3:	S: 0.73, P: 0.27	2.49
4:	S: 0.83, P: 0.17	2.51
5:	S: 0.84, P: 0.16	2.82
6:	S: 0.86, P: 0.14	3.3
7:	S: 0.94, P: 0.06	2.32
8:	S: 0.94, P: 0.06	2
9:	S: 0.95, P: 0.05	1.86
10:	S: 0.98, P: 0.02	1.86
11:	S: 1, P: 0	1.92
12:	S: 1, P: 0	1.83
13:	S: 1, P: 0	1.79
14:	S: 1, P: 0	2.04
15:	S: 1, P: 0	2.22
16:	S: 1, P: 0	1.89
17:	S: 1, P: 0	1.88
18:	S: 1, P: 0	2.23
19:	S: 1, P: 0	2.53
20:	S: 1, P: 0	1.77
21:	S: 1, P: 0	1.77
22:	S: 0.8, P: 0.2	1.82
23:	S: 0.79, P: 0.21	1.53
24:	S: 0.49, P: 0.51	1.26
25:	S: 0.49, P: 0.51	1.28
26:	S: 0.95, P: 0.05	2.22
27:	S: 0.83, P: 0.17	3.87
28:	S: 0.14, P: 0.86	28.92
29:	S: 0.71, P: 0.29	2.33
30:	S: 0.46, P: 0.54	2.75
31:	S: 0.15, P: 0.85	2.57
32:	S: 0.14, P: 0.86	2.49
33:	S: 0.07, P: 0.93	2.63
34:	S: 0, P: 1	2.44
35:	S: 0.24, P: 0.76	2.22
36:	S: 0.07, P: 0.93	2.62
37:	S: 0.02, P: 0.98	1.32
38:	S: 0.02, P: 0.98	1.34
39:	S: 0.53, P: 0.47	3.29
40:	S: 0.41, P: 0.59	4.93
41:	S: 0.39, P: 0.61	6.12
42:	S: 0.41, P: 0.59	7.15
43:	S: 0.07, P: 0.93	10.46
44:	S: 0.73, P: 0.27	10.61
45:	S: 0.4, P: 0.6	4.83
46:	S: 0.46, P: 0.54	3.77
47:	S: 0.97, P: 0.03	2.22

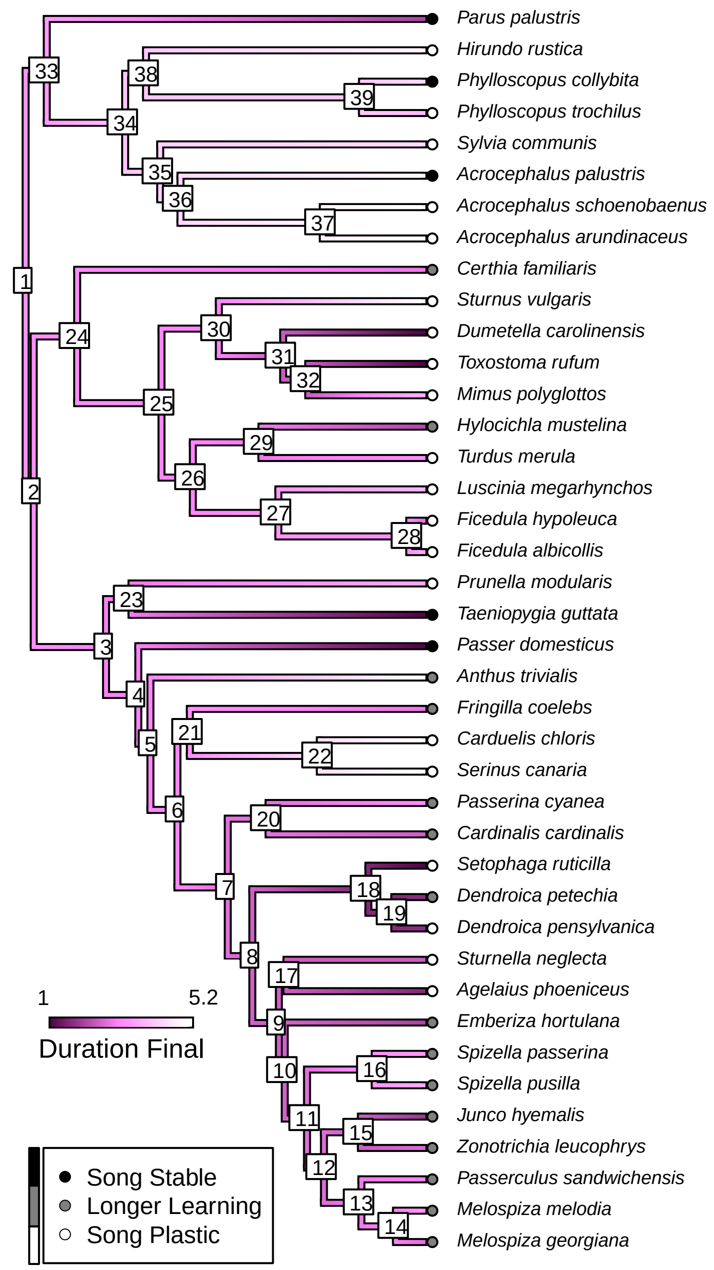

Node	State Likelihood	Trait
1:	E: 0.07, D: 0.1, P: 0.83	2.74
2:	E: 0.07, D: 0.11, P: 0.83	2.68
3:	E: 0.1, D: 0.25, P: 0.65	2.38
4:	E: 0.09, D: 0.41, P: 0.5	2.43
5:	E: 0.06, D: 0.49, P: 0.46	2.75
6:	E: 0.02, D: 0.55, P: 0.43	3.24
7:	E: 0.01, D: 0.7, P: 0.3	2.29
8:	E: 0, D: 0.67, P: 0.32	1.97
9:	E: 0, D: 0.7, P: 0.3	1.87
10:	E: 0, D: 0.87, P: 0.13	1.89
11:	E: 0, D: 0.99, P: 0.01	2.01
12:	E: 0, D: 1, P: 0	1.98
13:	E: 0, D: 1, P: 0	2.15
14:	E: 0, D: 1, P: 0	2.25
15:	E: 0, D: 1, P: 0	1.78
16:	E: 0, D: 1, P: 0	2.56
17:	E: 0.01, D: 0.55, P: 0.45	1.83
18:	E: 0, D: 0.22, P: 0.78	1.22
19:	E: 0, D: 0.24, P: 0.76	1.26
20:	E: 0.01, D: 0.85, P: 0.14	2.2
21:	E: 0.03, D: 0.54, P: 0.44	3.81
22:	E: 0.01, D: 0.05, P: 0.93	28.79
23:	E: 0.14, D: 0.23, P: 0.64	2.25
24:	E: 0.05, D: 0.1, P: 0.85	2.58
25:	E: 0.01, D: 0.02, P: 0.97	2.51
26:	E: 0.01, D: 0.02, P: 0.97	2.44
27:	E: 0.01, D: 0.01, P: 0.98	2.6
28:	E: 0, D: 0, P: 1	2.44
29:	E: 0.02, D: 0.1, P: 0.88	2.2
30:	E: 0, D: 0.01, P: 0.99	2.58
31:	E: 0, D: 0, P: 1	1.31
32:	E: 0, D: 0, P: 1	1.33
33:	E: 0.08, D: 0.08, P: 0.84	2.89
34:	E: 0.06, D: 0.02, P: 0.92	4.03
35:	E: 0.06, D: 0.02, P: 0.92	4.64
36:	E: 0.07, D: 0.02, P: 0.9	5.07
37:	E: 0.01, D: 0.01, P: 0.98	9.57
38:	E: 0.07, D: 0.03, P: 0.91	4.06
39:	E: 0.27, D: 0.02, P: 0.71	3.69

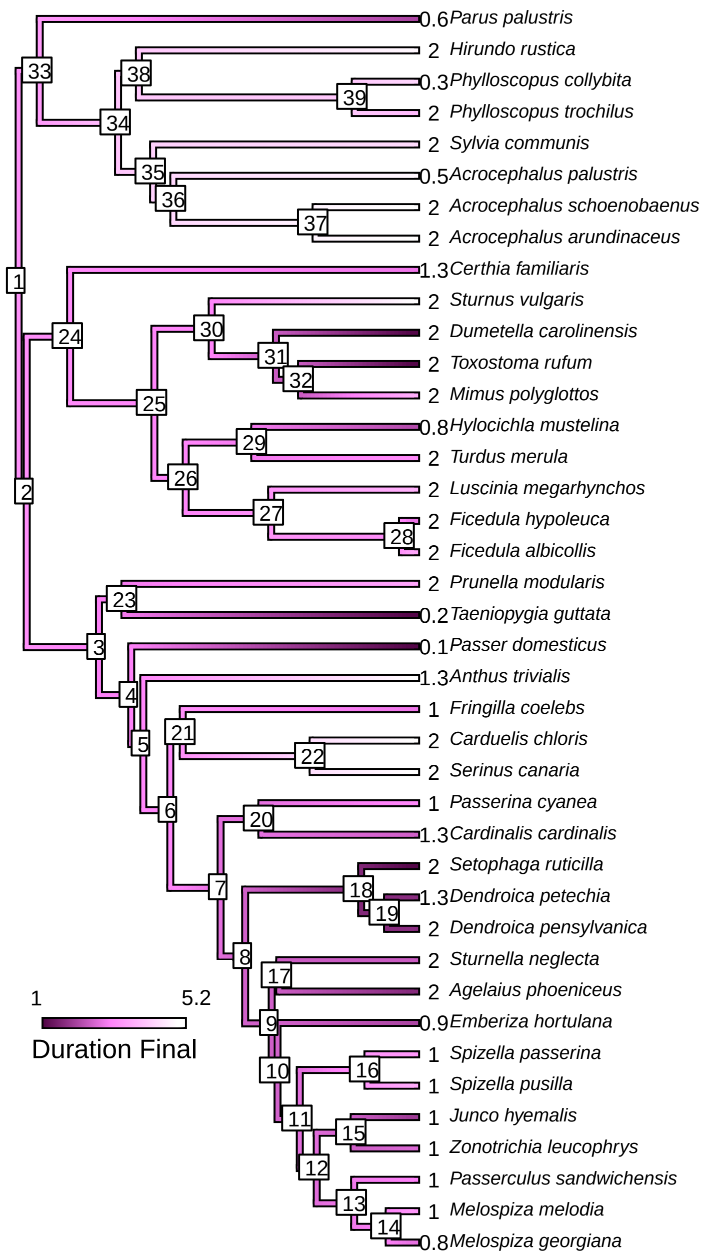

Node	Length of Plasticity	Trait
1:	1.36	2.74
2:	1.36	2.68
3:	1.24	2.38
4:	1.21	2.43
5:	1.24	2.75
6:	1.32	3.24
7:	1.34	2.29
8:	1.39	1.97
9:	1.37	1.87
10:	1.32	1.89
11:	1.18	2.01
12:	1.11	1.98
13:	1.02	2.15
14:	0.95	2.25
15:	1.05	1.78
16:	1.05	2.56
17:	1.43	1.83
18:	1.74	1.22
19:	1.69	1.26
20:	1.28	2.2
21:	1.34	3.81
22:	1.81	28.79
23:	1.22	2.25
24:	1.46	2.58
25:	1.68	2.51
26:	1.69	2.44
27:	1.86	2.6
28:	1.99	2.44
29:	1.56	2.2
30:	1.82	2.58
31:	1.92	1.31
32:	1.94	1.33
33:	1.34	2.89
34:	1.45	4.03
35:	1.48	4.64
36:	1.46	5.07
37:	1.86	9.57
38:	1.47	4.06
39:	1.18	3.69

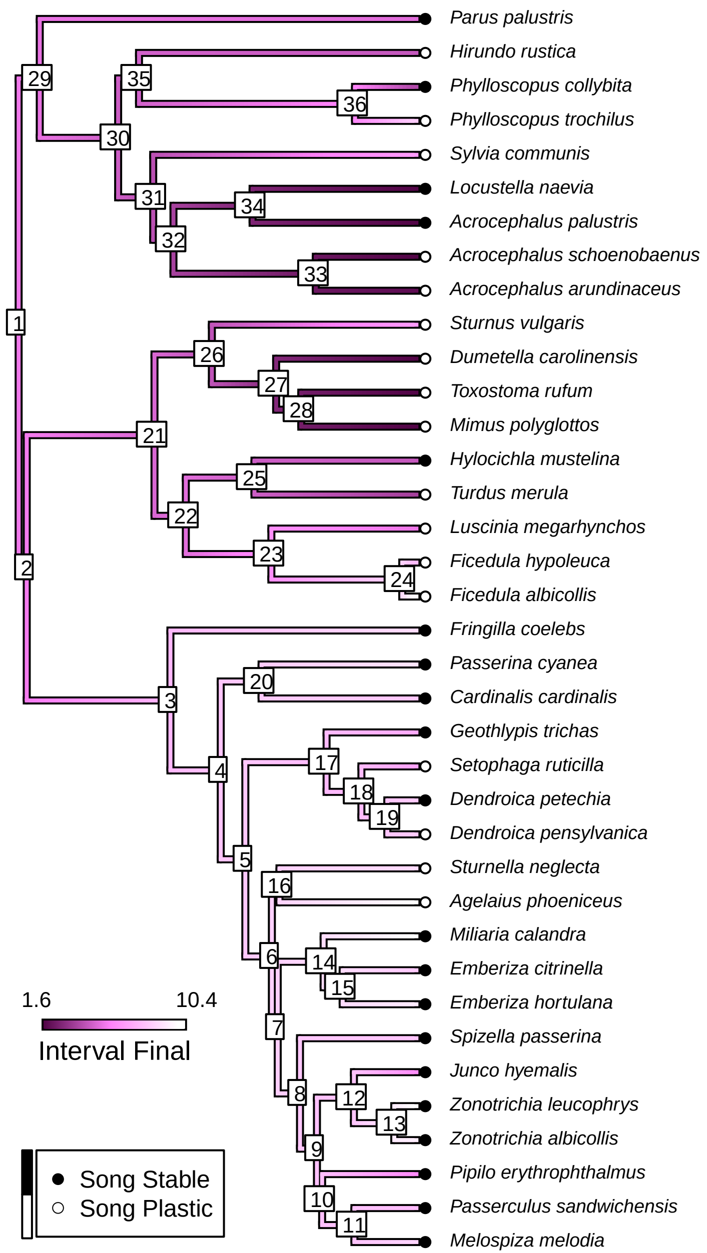

Node	State Likelihood	Trait
1:	S: 0.43, P: 0.57	3.28
2:	S: 0.43, P: 0.57	3.34
3:	S: 0.84, P: 0.16	5.91
4:	S: 0.93, P: 0.07	6.78
5:	S: 0.93, P: 0.07	7.06
6:	S: 0.94, P: 0.06	7.65
7:	S: 0.98, P: 0.02	7.59
8:	S: 0.99, P: 0.01	7.06
9:	S: 1, P: 0	6.72
10:	S: 1, P: 0	6.6
11:	S: 1, P: 0	6.76
12:	S: 1, P: 0	6.87
13:	S: 1, P: 0	9.25
14:	S: 0.99, P: 0.01	8.33
15:	S: 1, P: 0	8.2
16:	S: 0.8, P: 0.2	7.9
17:	S: 0.79, P: 0.21	6.3
18:	S: 0.48, P: 0.52	6.28
19:	S: 0.48, P: 0.52	6.81
20:	S: 0.95, P: 0.05	7.08
21:	S: 0.12, P: 0.88	2.64
22:	S: 0.12, P: 0.88	3.02
23:	S: 0.06, P: 0.94	4.58
24:	S: 0, P: 1	10.28
25:	S: 0.23, P: 0.77	2.91
26:	S: 0.06, P: 0.94	1.86
27:	S: 0.02, P: 0.98	0.92
28:	S: 0.02, P: 0.98	0.92
29:	S: 0.43, P: 0.57	3.13
30:	S: 0.35, P: 0.65	2.59
31:	S: 0.34, P: 0.66	2.18
32:	S: 0.37, P: 0.63	1.86
33:	S: 0.07, P: 0.93	1.6
34:	S: 0.71, P: 0.29	1.08
35:	S: 0.34, P: 0.66	2.72
36:	S: 0.45, P: 0.55	4.17

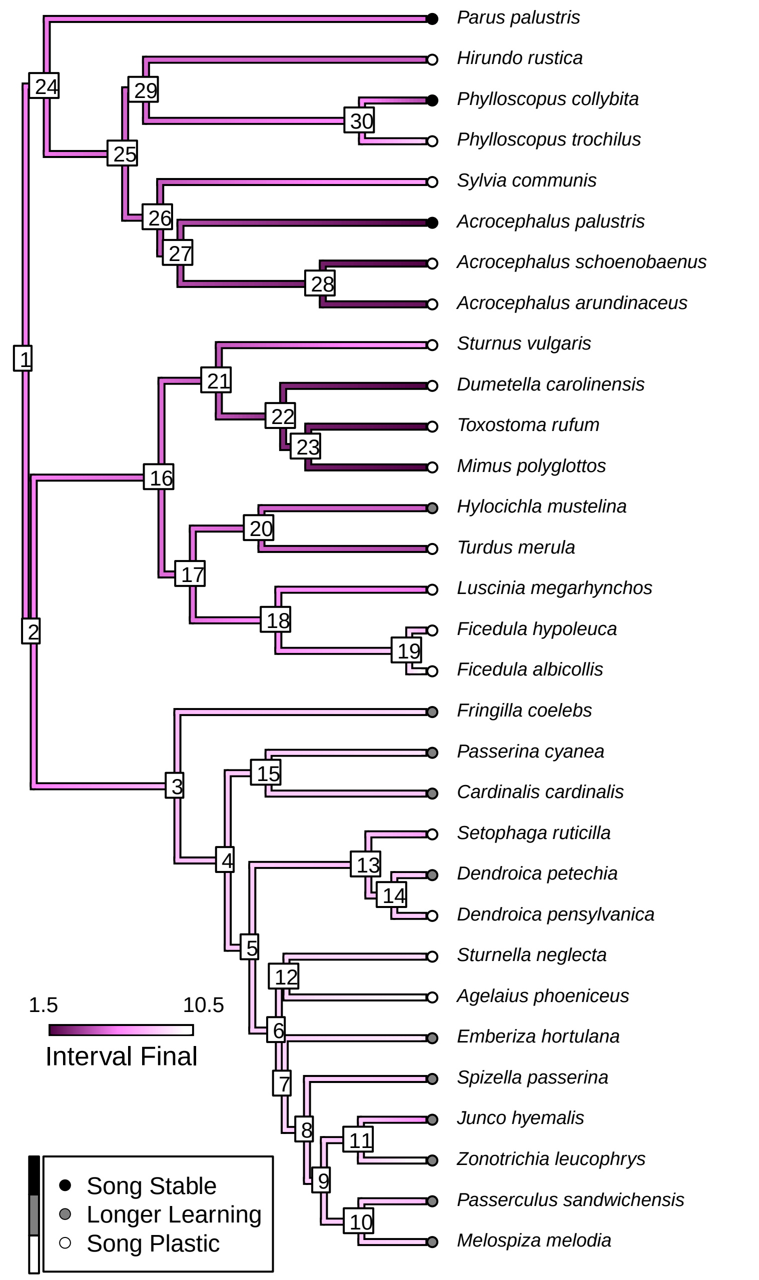

Node	State Likelihood	Trait
1:	E: 0.05, D: 0.09, P: 0.86	3.23
2:	E: 0.05, D: 0.09, P: 0.86	3.3
3:	E: 0.02, D: 0.77, P: 0.21	5.96
4:	E: 0, D: 0.84, P: 0.16	6.9
5:	E: 0, D: 0.8, P: 0.2	7.24
6:	E: 0, D: 0.81, P: 0.19	7.83
7:	E: 0, D: 0.92, P: 0.08	7.79
8:	E: 0, D: 0.99, P: 0.01	7.44
9:	E: 0, D: 1, P: 0	7.29
10:	E: 0, D: 1, P: 0	7.09
11:	E: 0, D: 1, P: 0	7.15
12:	E: 0.01, D: 0.6, P: 0.39	8.07
13:	E: 0, D: 0.25, P: 0.74	6.42
14:	E: 0, D: 0.27, P: 0.73	6.86
15:	E: 0.01, D: 0.93, P: 0.07	7.17
16:	E: 0, D: 0.01, P: 0.99	2.63
17:	E: 0, D: 0.01, P: 0.99	3.02
18:	E: 0, D: 0, P: 0.99	4.57
19:	E: 0, D: 0, P: 1	10.28
20:	E: 0.01, D: 0.08, P: 0.91	2.91
21:	E: 0, D: 0, P: 0.99	1.85
22:	E: 0, D: 0, P: 1	0.92
23:	E: 0, D: 0, P: 1	0.92
24:	E: 0.06, D: 0.07, P: 0.87	3.08
25:	E: 0.04, D: 0.01, P: 0.95	2.49
26:	E: 0.04, D: 0.01, P: 0.95	2.07
27:	E: 0.05, D: 0.01, P: 0.94	1.73
28:	E: 0.01, D: 0, P: 0.99	1.57
29:	E: 0.04, D: 0.01, P: 0.94	2.63
30:	E: 0.23, D: 0.01, P: 0.76	4.15

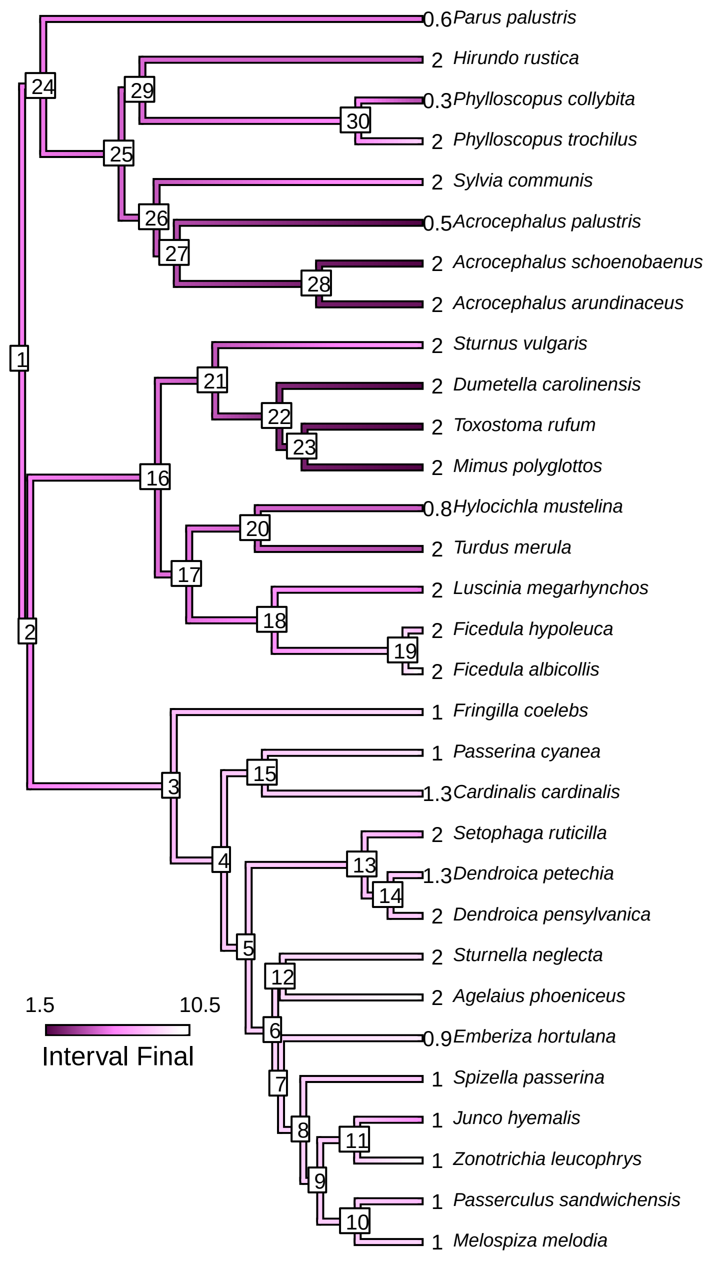

Node	Length of Plasticity	Trait
1:	1.44	3.23
2:	1.45	3.3
3:	1.33	5.96
4:	1.35	6.9
5:	1.4	7.24
6:	1.38	7.83
7:	1.33	7.79
8:	1.2	7.44
9:	1.14	7.29
10:	1.06	7.09
11:	1.06	7.15
12:	1.44	8.07
13:	1.74	6.42
14:	1.69	6.86
15:	1.29	7.17
16:	1.71	2.63
17:	1.71	3.02
18:	1.87	4.57
19:	1.99	10.28
20:	1.58	2.91
21:	1.84	1.85
22:	1.93	0.92
23:	1.95	0.92
24:	1.41	3.08
25:	1.48	2.49
26:	1.5	2.07
27:	1.48	1.73
28:	1.86	1.57
29:	1.49	2.63
30:	1.18	4.15

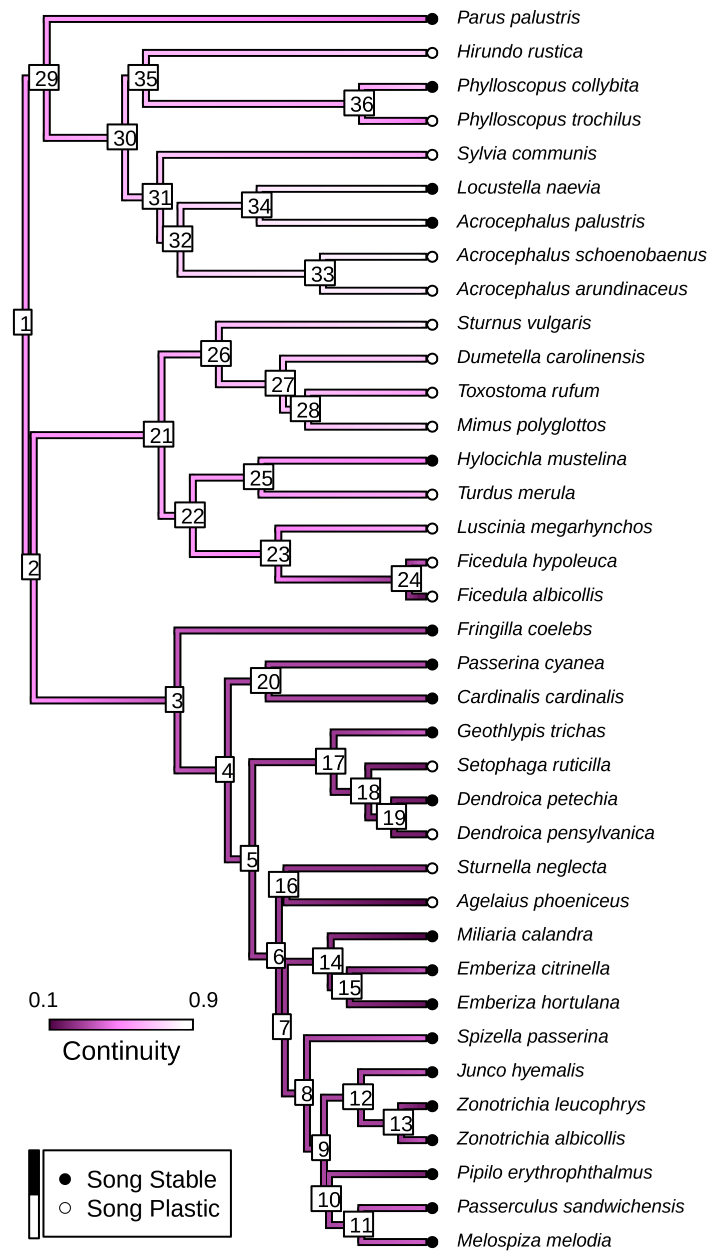

Node	State Likelihood	Trait
1:	S: 0.43, P: 0.57	0.39
2:	S: 0.43, P: 0.57	0.38
3:	S: 0.84, P: 0.16	0.24
4:	S: 0.93, P: 0.07	0.21
5:	S: 0.93, P: 0.07	0.19
6:	S: 0.94, P: 0.06	0.18
7:	S: 0.98, P: 0.02	0.18
8:	S: 0.99, P: 0.01	0.2
9:	S: 1, P: 0	0.2
10:	S: 1, P: 0	0.21
11:	S: 1, P: 0	0.23
12:	S: 1, P: 0	0.21
13:	S: 1, P: 0	0.19
14:	S: 0.99, P: 0.01	0.17
15:	S: 1, P: 0	0.17
16:	S: 0.8, P: 0.2	0.17
17:	S: 0.79, P: 0.21	0.19
18:	S: 0.48, P: 0.52	0.16
19:	S: 0.48, P: 0.52	0.16
20:	S: 0.95, P: 0.05	0.21
21:	S: 0.12, P: 0.88	0.43
22:	S: 0.12, P: 0.88	0.4
23:	S: 0.06, P: 0.94	0.33
24:	S: 0, P: 1	0.19
25:	S: 0.23, P: 0.77	0.4
26:	S: 0.06, P: 0.94	0.52
27:	S: 0.02, P: 0.98	0.55
28:	S: 0.02, P: 0.98	0.56
29:	S: 0.43, P: 0.57	0.41
30:	S: 0.35, P: 0.65	0.52
31:	S: 0.34, P: 0.66	0.58
32:	S: 0.37, P: 0.63	0.64
33:	S: 0.07, P: 0.93	0.8
34:	S: 0.71, P: 0.29	0.78
35:	S: 0.34, P: 0.66	0.52
36:	S: 0.45, P: 0.55	0.44

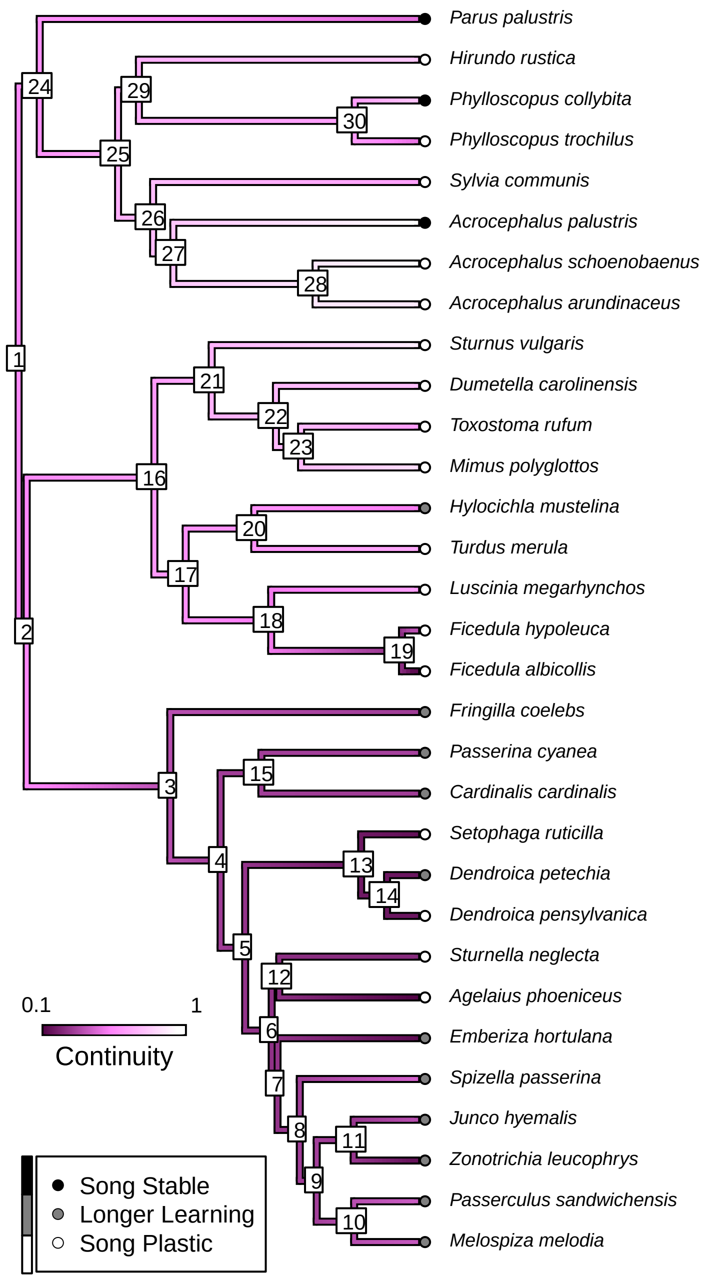

Node	State Likelihood	Trait
1:	E: 0.05, D: 0.09, P: 0.86	0.38
2:	E: 0.05, D: 0.09, P: 0.86	0.37
3:	E: 0.02, D: 0.77, P: 0.21	0.23
4:	E: 0, D: 0.84, P: 0.16	0.2
5:	E: 0, D: 0.8, P: 0.2	0.18
6:	E: 0, D: 0.81, P: 0.19	0.18
7:	E: 0, D: 0.92, P: 0.08	0.18
8:	E: 0, D: 0.99, P: 0.01	0.2
9:	E: 0, D: 1, P: 0	0.21
10:	E: 0, D: 1, P: 0	0.23
11:	E: 0, D: 1, P: 0	0.2
12:	E: 0.01, D: 0.6, P: 0.39	0.17
13:	E: 0, D: 0.25, P: 0.74	0.16
14:	E: 0, D: 0.27, P: 0.73	0.15
15:	E: 0.01, D: 0.93, P: 0.07	0.2
16:	E: 0, D: 0.01, P: 0.99	0.43
17:	E: 0, D: 0.01, P: 0.99	0.4
18:	E: 0, D: 0, P: 0.99	0.33
19:	E: 0, D: 0, P: 1	0.19
20:	E: 0.01, D: 0.08, P: 0.91	0.4
21:	E: 0, D: 0, P: 0.99	0.52
22:	E: 0, D: 0, P: 1	0.55
23:	E: 0, D: 0, P: 1	0.55
24:	E: 0.06, D: 0.07, P: 0.87	0.4
25:	E: 0.04, D: 0.01, P: 0.95	0.51
26:	E: 0.04, D: 0.01, P: 0.95	0.56
27:	E: 0.05, D: 0.01, P: 0.94	0.61
28:	E: 0.01, D: 0, P: 0.99	0.79
29:	E: 0.04, D: 0.01, P: 0.94	0.51
30:	E: 0.23, D: 0.01, P: 0.76	0.44

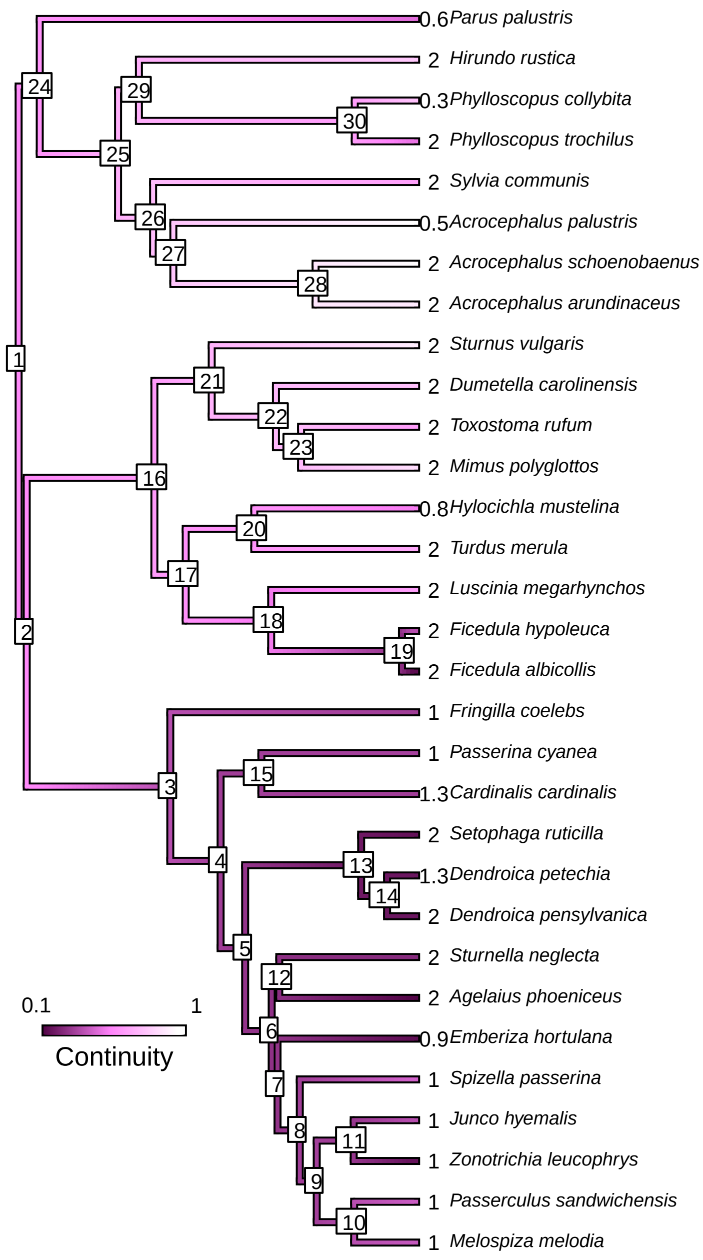

Node	Length of Plasticity	Trait
1:	1.44	0.38
2:	1.45	0.37
3:	1.33	0.23
4:	1.35	0.2
5:	1.4	0.18
6:	1.38	0.18
7:	1.33	0.18
8:	1.2	0.2
9:	1.14	0.21
10:	1.06	0.23
11:	1.06	0.2
12:	1.44	0.17
13:	1.74	0.16
14:	1.69	0.15
15:	1.29	0.2
16:	1.71	0.43
17:	1.71	0.4
18:	1.87	0.33
19:	1.99	0.19
20:	1.58	0.4
21:	1.84	0.52
22:	1.93	0.55
23:	1.95	0.55
24:	1.41	0.4
25:	1.48	0.51
26:	1.5	0.56
27:	1.48	0.61
28:	1.86	0.79
29:	1.49	0.51
30:	1.18	0.44

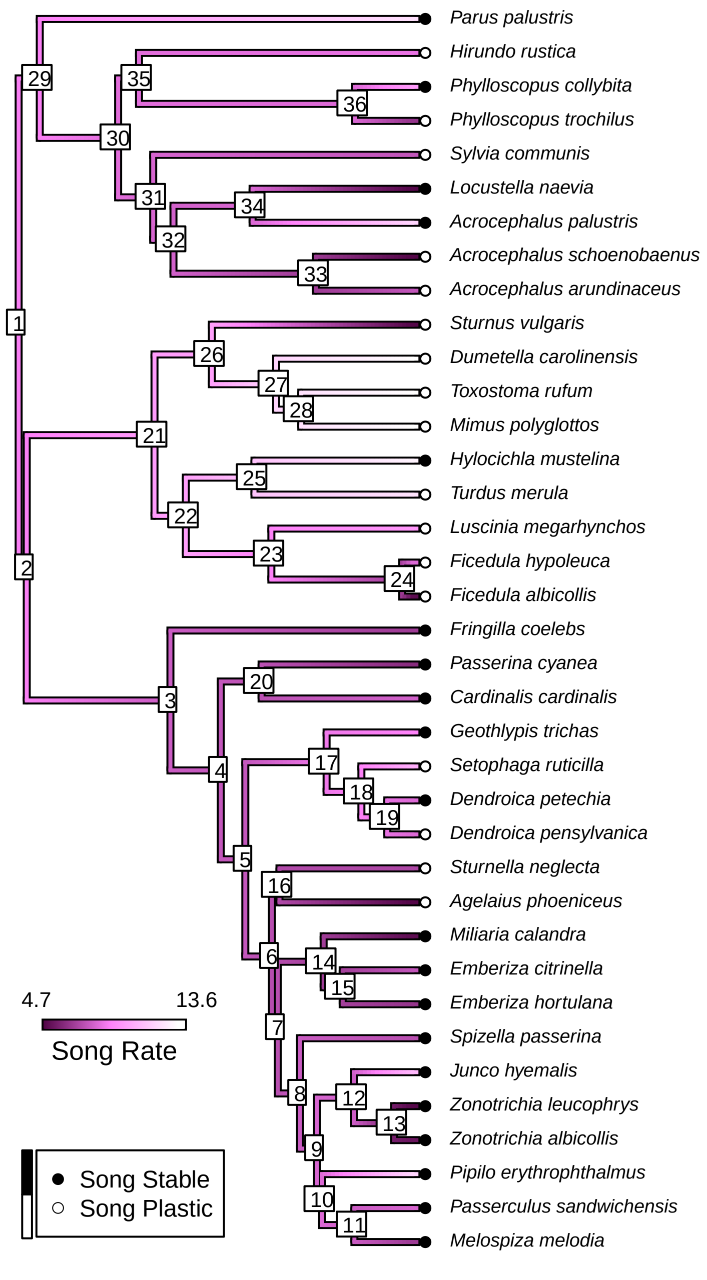

Node	State Likelihood	Trait
1:	S: 0.43, P: 0.57	7.71
2:	S: 0.43, P: 0.57	7.77
3:	S: 0.84, P: 0.16	6.7
4:	S: 0.93, P: 0.07	6.53
5:	S: 0.93, P: 0.07	6.53
6:	S: 0.94, P: 0.06	6.22
7:	S: 0.98, P: 0.02	6.26
8:	S: 0.99, P: 0.01	6.64
9:	S: 1, P: 0	6.98
10:	S: 1, P: 0	7.11
11:	S: 1, P: 0	6.74
12:	S: 1, P: 0	6.85
13:	S: 1, P: 0	5.2
14:	S: 0.99, P: 0.01	5.87
15:	S: 1, P: 0	5.96
16:	S: 0.8, P: 0.2	6.07
17:	S: 0.79, P: 0.21	7.57
18:	S: 0.48, P: 0.52	7.9
19:	S: 0.48, P: 0.52	7.4
20:	S: 0.95, P: 0.05	6.39
21:	S: 0.12, P: 0.88	9.96
22:	S: 0.12, P: 0.88	9.64
23:	S: 0.06, P: 0.94	7.58
24:	S: 0, P: 1	4.63
25:	S: 0.23, P: 0.77	10.89
26:	S: 0.06, P: 0.94	11.82
27:	S: 0.02, P: 0.98	24.98
28:	S: 0.02, P: 0.98	24.88
29:	S: 0.43, P: 0.57	7.56
30:	S: 0.35, P: 0.65	6.39
31:	S: 0.34, P: 0.66	5.75
32:	S: 0.37, P: 0.63	5.36
33:	S: 0.07, P: 0.93	4.6
34:	S: 0.71, P: 0.29	4.42
35:	S: 0.34, P: 0.66	6.51
36:	S: 0.45, P: 0.55	7.04

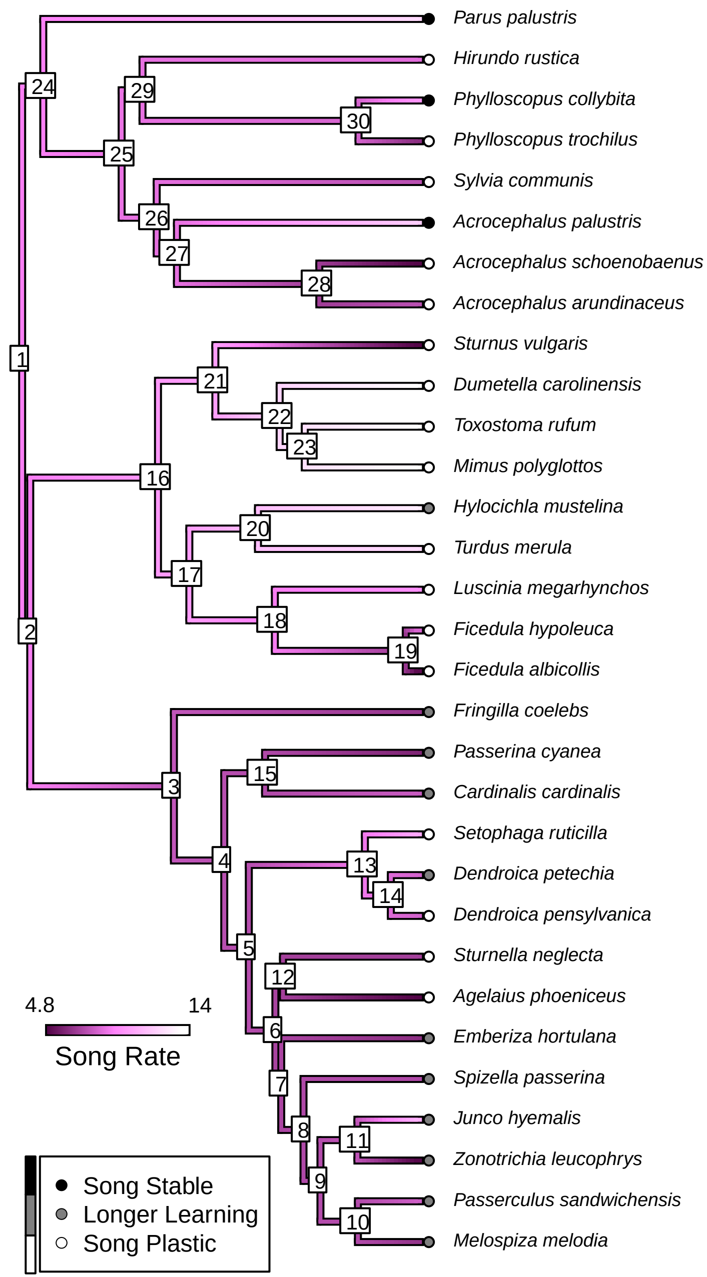

Node	State Likelihood	Trait
1:	E: 0.05, D: 0.09, P: 0.86	8.27
2:	E: 0.05, D: 0.09, P: 0.86	8.28
3:	E: 0.02, D: 0.77, P: 0.21	6.77
4:	E: 0, D: 0.84, P: 0.16	6.49
5:	E: 0, D: 0.8, P: 0.2	6.43
6:	E: 0, D: 0.81, P: 0.19	6.09
7:	E: 0, D: 0.92, P: 0.08	6.11
8:	E: 0, D: 0.99, P: 0.01	6.28
9:	E: 0, D: 1, P: 0	6.4
10:	E: 0, D: 1, P: 0	6.41
11:	E: 0, D: 1, P: 0	6.65
12:	E: 0.01, D: 0.6, P: 0.39	5.96
13:	E: 0, D: 0.25, P: 0.74	7.82
14:	E: 0, D: 0.27, P: 0.73	7.38
15:	E: 0.01, D: 0.93, P: 0.07	6.37
16:	E: 0, D: 0.01, P: 0.99	10.17
17:	E: 0, D: 0.01, P: 0.99	9.78
18:	E: 0, D: 0, P: 0.99	7.63
19:	E: 0, D: 0, P: 1	4.63
20:	E: 0.01, D: 0.08, P: 0.91	10.98
21:	E: 0, D: 0, P: 0.99	11.96
22:	E: 0, D: 0, P: 1	25.11
23:	E: 0, D: 0, P: 1	24.97
24:	E: 0.06, D: 0.07, P: 0.87	8.24
25:	E: 0.04, D: 0.01, P: 0.95	7.5
26:	E: 0.04, D: 0.01, P: 0.95	7.24
27:	E: 0.05, D: 0.01, P: 0.94	7.15
28:	E: 0.01, D: 0, P: 0.99	4.96
29:	E: 0.04, D: 0.01, P: 0.94	7.47
30:	E: 0.23, D: 0.01, P: 0.76	7.17

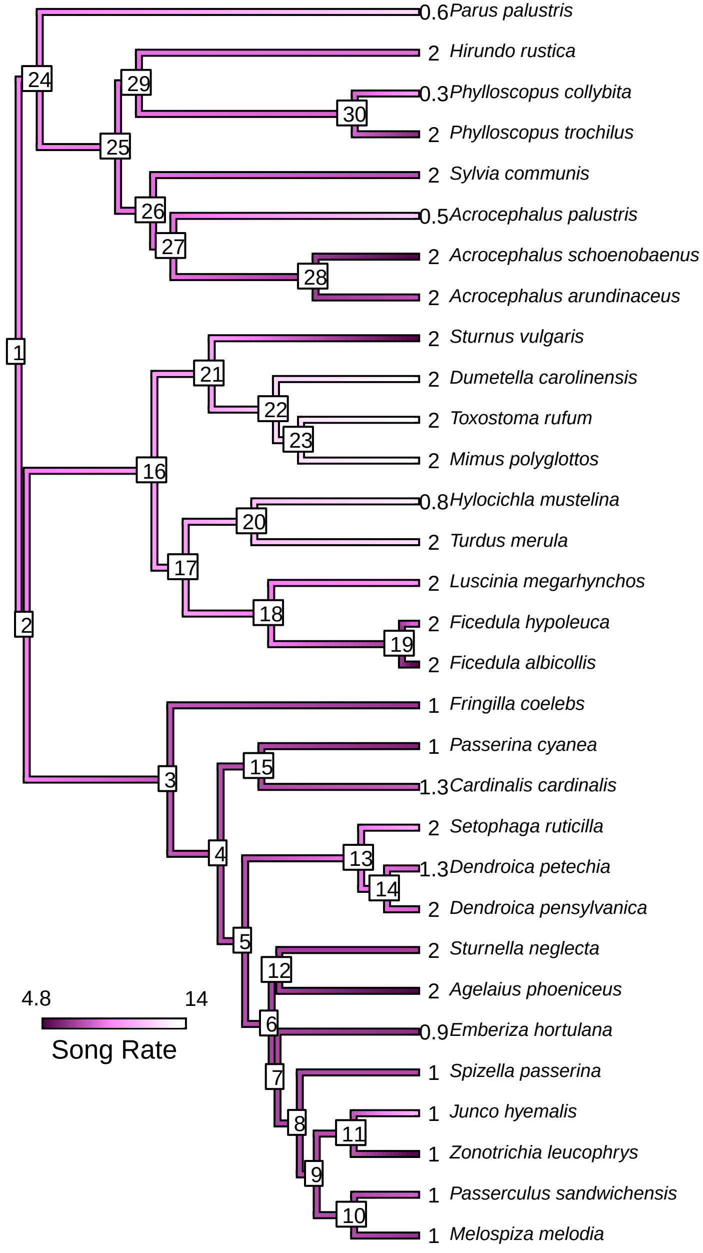

Node	Length of Plasticity	Trait
1:	1.44	8.27
2:	1.45	8.28
3:	1.33	6.77
4:	1.35	6.49
5:	1.4	6.43
6:	1.38	6.09
7:	1.33	6.11
8:	1.2	6.28
9:	1.14	6.4
10:	1.06	6.41
11:	1.06	6.65
12:	1.44	5.96
13:	1.74	7.82
14:	1.69	7.38
15:	1.29	6.37
16:	1.71	10.17
17:	1.71	9.78
18:	1.87	7.63
19:	1.99	4.63
20:	1.58	10.98
21:	1.84	11.96
22:	1.93	25.11
23:	1.95	24.97
24:	1.41	8.24
25:	1.48	7.5
26:	1.5	7.24
27:	1.48	7.15
28:	1.86	4.96
29:	1.49	7.47
30:	1.18	7.17
