# Supplementary material for: Correlated evolution between repertoire size and song plasticity predicts that sexual selection on song promotes open-ended learning
Source: eLife. 2019 Sep 3;8:e44454. doi: 10.7554/eLife.44454 (PMC6721395; doi:10.7554/eLife.44454)
Supplement: Figure 6—source data 1. — On page 1, the top two panels depict the mean rates from all runs across the values of syllable repertoire in the lower and upper half of thresholds, respectively. The bottom panel shows the p-values from the 100 runs per threshold, plotted against threshold. Colored bars denote low and high threshold segments. The blue line denotes p=0.05. On page 2, the top four panels depict the mean rates from all runs across the values of syllable repertoire in the first (top left), second (top right), third (middle left), and fourth (middle right) quartiles of the thresholds. The bottom panel follows the pattern of the bottom panel from page 1. On page 3, the five panels (top left and right, middle left and right, and bottom left) depict the mean rates from all runs across the values of syllable repertoire in the five quintiles of the of the thresholds, respectively. The bottom panel follows the pattern of the bottom panels from pages 1 and 2. The three-page pattern repeats for each song feature. [file elife-44454-fig6-data1.pdf]

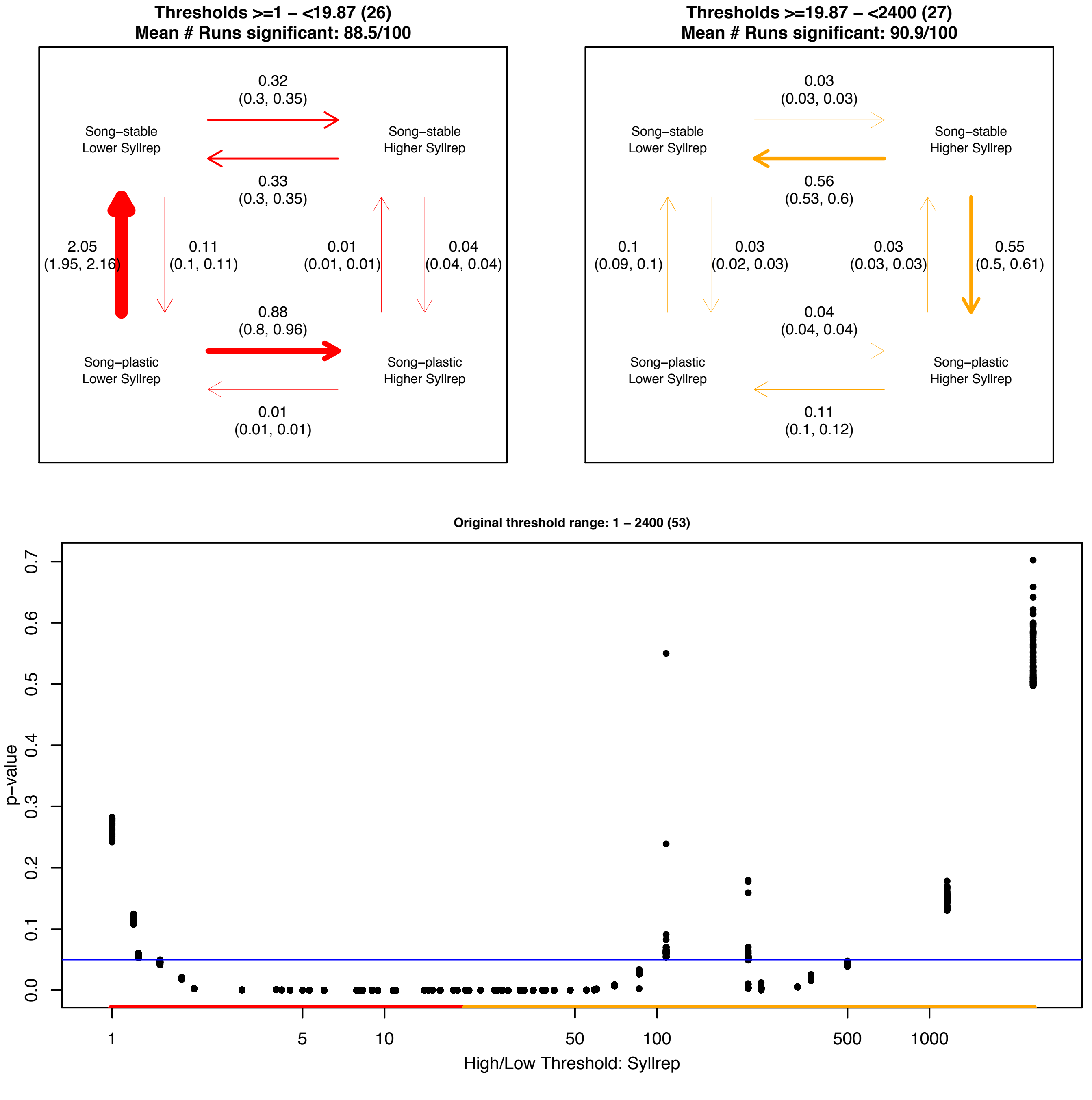

Thresholds >=1 – <19.87 (26)
Mean # Runs significant: 88.5/100
Song–stable Lower Syllrep
Song–stable Higher Syllrep
Song–plastic Lower Syllrep
Song–plastic Higher Syllrep
0.32 (0.3, 0.35)
0.33 (0.3, 0.35)
2.05 (1.95, 2.16)
0.11 (0.1, 0.11)
0.01 (0.01, 0.01)
0.04 (0.04, 0.04)
0.88 (0.8, 0.96)
0.01 (0.01, 0.01)
Thresholds >=19.87 – <2400 (27)
Mean # Runs significant: 90.9/100
Song–stable Lower Syllrep
Song–stable Higher Syllrep
Song–plastic Lower Syllrep
Song–plastic Higher Syllrep
0.03 (0.03, 0.03)
0.56 (0.53, 0.6)
0.1 (0.09, 0.1)
0.03 (0.02, 0.03)
0.03 (0.03, 0.03)
0.55 (0.5, 0.61)
0.04 (0.04, 0.04)
0.11 (0.1, 0.12)
Original threshold range: 1 – 2400 (53)
p–value
0.0 0.1 0.2 0.3 0.4 0.5 0.6 0.7
1 5 10 50 100 500 1000
High/Low Threshold: Syllrep

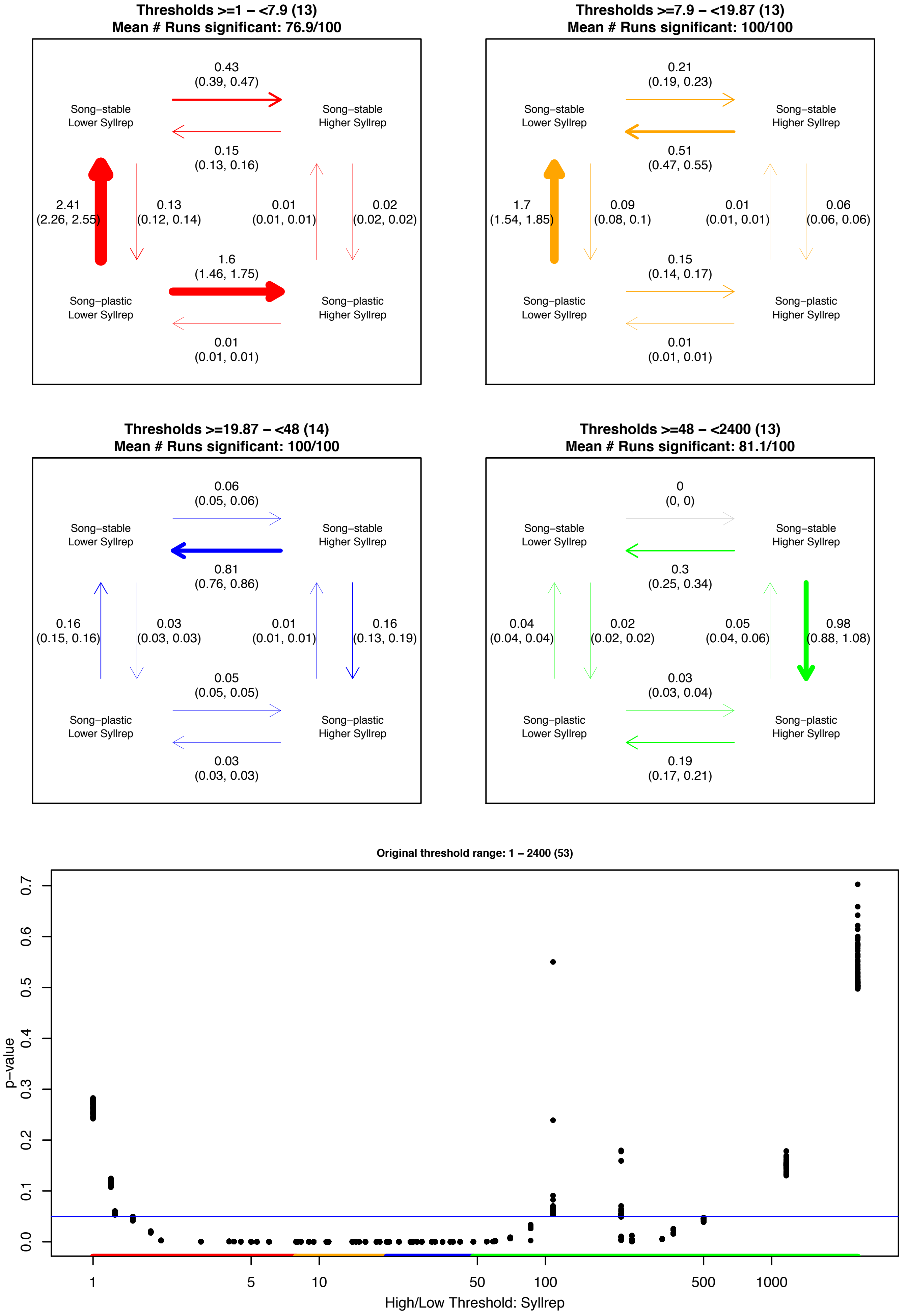
Thresholds >=1 – <7.9 (13)
Mean # Runs significant: 76.9/100
Song–stable Lower Syllrep
Song–stable Higher Syllrep
Song–plastic Lower Syllrep
Song–plastic Higher Syllrep
0.43 (0.39, 0.47)
0.15 (0.13, 0.16)
2.41 (2.26, 2.55)
0.13 (0.12, 0.14)
0.01 (0.01, 0.01)
0.02 (0.02, 0.02)
1.6 (1.46, 1.75)
0.01 (0.01, 0.01)
Thresholds >=7.9 – <19.87 (13)
Mean # Runs significant: 100/100
Song–stable Lower Syllrep
Song–stable Higher Syllrep
Song–plastic Lower Syllrep
Song–plastic Higher Syllrep
0.21 (0.19, 0.23)
0.51 (0.47, 0.55)
1.7 (1.54, 1.85)
0.09 (0.08, 0.1)
0.01 (0.01, 0.01)
0.06 (0.06, 0.06)
0.15 (0.14, 0.17)
0.01 (0.01, 0.01)
Thresholds >=19.87 – <48 (14)
Mean # Runs significant: 100/100
Song–stable Lower Syllrep
Song–stable Higher Syllrep
Song–plastic Lower Syllrep
Song–plastic Higher Syllrep
0.06 (0.05, 0.06)
0.81 (0.76, 0.86)
0.16 (0.15, 0.16)
0.03 (0.03, 0.03)
0.01 (0.01, 0.01)
0.16 (0.13, 0.19)
0.05 (0.05, 0.05)
0.03 (0.03, 0.03)
Thresholds >=48 – <2400 (13)
Mean # Runs significant: 81.1/100
Song–stable Lower Syllrep
Song–stable Higher Syllrep
Song–plastic Lower Syllrep
Song–plastic Higher Syllrep
0 (0, 0)
0.3 (0.25, 0.34)
0.04 (0.04, 0.04)
0.02 (0.02, 0.02)
0.05 (0.04, 0.06)
0.98 (0.88, 1.08)
0.03 (0.03, 0.04)
0.19 (0.17, 0.21)
Original threshold range: 1 – 2400 (53)
p–value
0.0
0.1
0.2
0.3
0.4
0.5
0.6
0.7
1
5
10
50
100
500
1000
High/Low Threshold: Syllrep

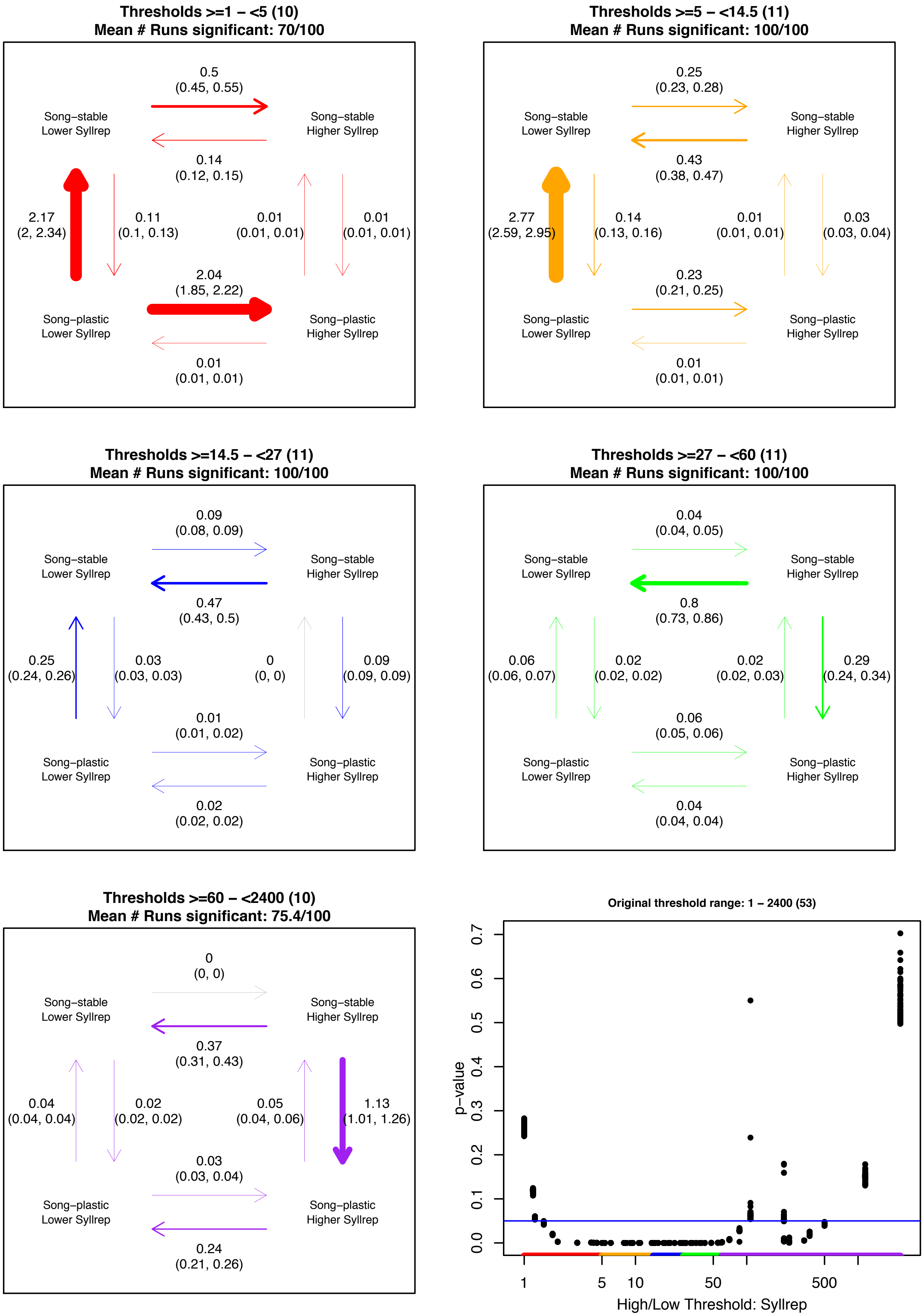

Thresholds >=1 – <5 (10)
Mean # Runs significant: 70/100
0.5
(0.45, 0.55)
Song–stable Lower Syllrep
Song–stable Higher Syllrep
0.14
(0.12, 0.15)
2.17
(2, 2.34)
0.11
(0.1, 0.13)
0.01
(0.01, 0.01)
0.01
(0.01, 0.01)
2.04
(1.85, 2.22)
Song–plastic Lower Syllrep
Song–plastic Higher Syllrep
0.01
(0.01, 0.01)
Thresholds >=5 – <14.5 (11)
Mean # Runs significant: 100/100
0.25
(0.23, 0.28)
Song–stable Lower Syllrep
Song–stable Higher Syllrep
0.43
(0.38, 0.47)
2.77
(2.59, 2.95)
0.14
(0.13, 0.16)
0.01
(0.01, 0.01)
0.03
(0.03, 0.04)
0.23
(0.21, 0.25)
Song–plastic Lower Syllrep
Song–plastic Higher Syllrep
0.01
(0.01, 0.01)
Thresholds >=14.5 – <27 (11)
Mean # Runs significant: 100/100
0.09
(0.08, 0.09)
Song–stable Lower Syllrep
Song–stable Higher Syllrep
0.47
(0.43, 0.5)
0.25
(0.24, 0.26)
0.03
(0.03, 0.03)
0
(0, 0)
0.09
(0.09, 0.09)
0.01
(0.01, 0.02)
Song–plastic Lower Syllrep
Song–plastic Higher Syllrep
0.02
(0.02, 0.02)
Thresholds >=27 – <60 (11)
Mean # Runs significant: 100/100
0.04
(0.04, 0.05)
Song–stable Lower Syllrep
Song–stable Higher Syllrep
0.8
(0.73, 0.86)
0.06
(0.06, 0.07)
0.02
(0.02, 0.02)
0.02
(0.02, 0.03)
0.29
(0.24, 0.34)
0.06
(0.05, 0.06)
Song–plastic Lower Syllrep
Song–plastic Higher Syllrep
0.04
(0.04, 0.04)
Thresholds >=60 – <2400 (10)
Mean # Runs significant: 75.4/100
0
(0, 0)
Song–stable Lower Syllrep
Song–stable Higher Syllrep
0.37
(0.31, 0.43)
0.04
(0.04, 0.04)
0.02
(0.02, 0.02)
0.05
(0.04, 0.06)
1.13
(1.01, 1.26)
0.03
(0.03, 0.04)
Song–plastic Lower Syllrep
Song–plastic Higher Syllrep
0.24
(0.21, 0.26)
Original threshold range: 1 – 2400 (53)
p–value
0.0
0.1
0.2
0.3
0.4
0.5
0.6
0.7
1
5
10
50
500
High/Low Threshold: Syllrep

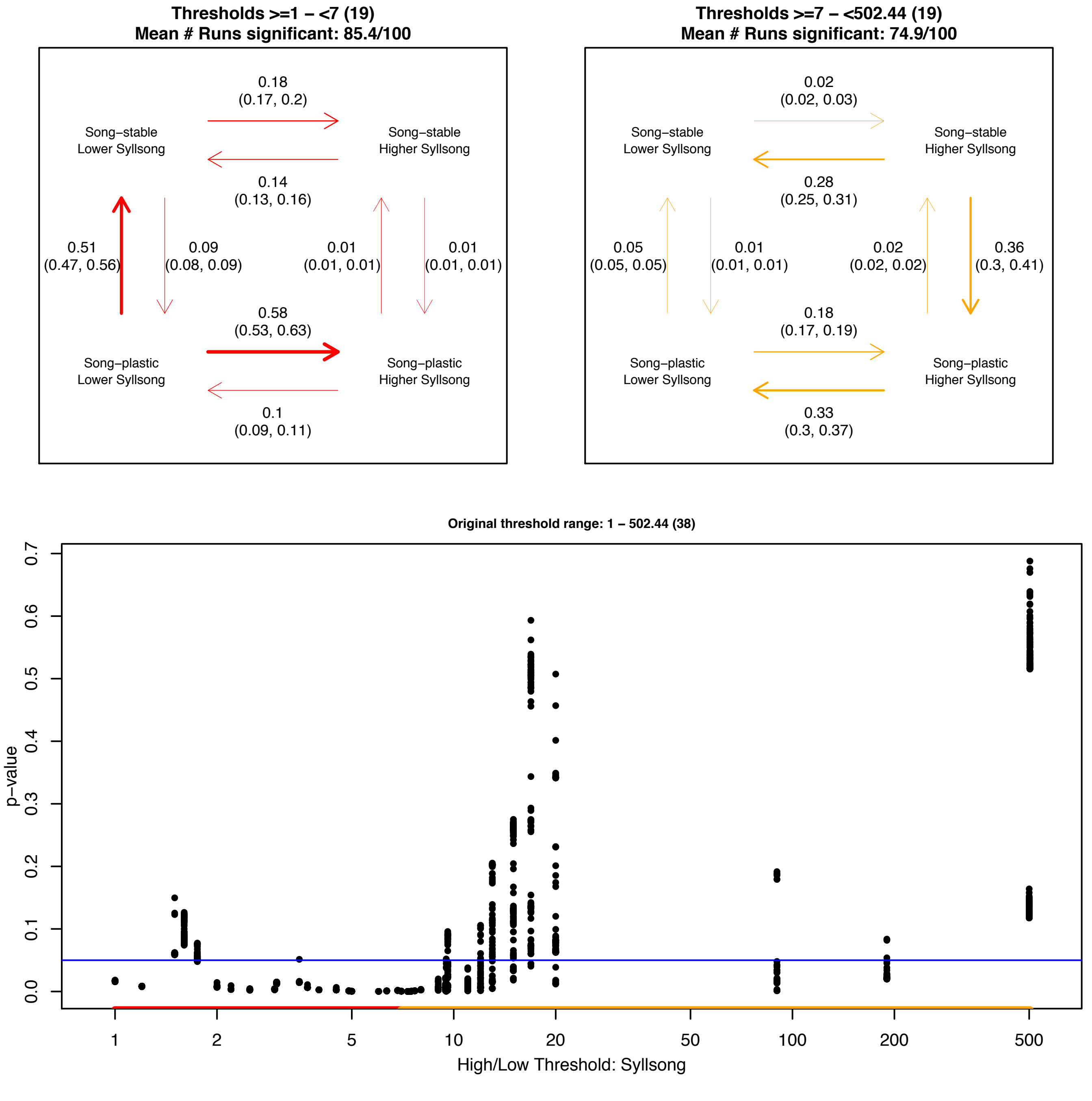

Thresholds >=1 – <7 (19)
Mean # Runs significant: 85.4/100
Song–stable Lower Syllsong
Song–stable Higher Syllsong
Song–plastic Lower Syllsong
Song–plastic Higher Syllsong
0.18 (0.17, 0.2)
0.14 (0.13, 0.16)
0.51 (0.47, 0.56)
0.09 (0.08, 0.09)
0.01 (0.01, 0.01)
0.01 (0.01, 0.01)
0.58 (0.53, 0.63)
0.1 (0.09, 0.11)
Thresholds >=7 – <502.44 (19)
Mean # Runs significant: 74.9/100
Song–stable Lower Syllsong
Song–stable Higher Syllsong
Song–plastic Lower Syllsong
Song–plastic Higher Syllsong
0.02 (0.02, 0.03)
0.28 (0.25, 0.31)
0.05 (0.05, 0.05)
0.01 (0.01, 0.01)
0.02 (0.02, 0.02)
0.36 (0.3, 0.41)
0.18 (0.17, 0.19)
0.33 (0.3, 0.37)
Original threshold range: 1 – 502.44 (38)
p–value
0.0
0.1
0.2
0.3
0.4
0.5
0.6
0.7
1
2
5
10
20
50
100
200
500
High/Low Threshold: Syllsong

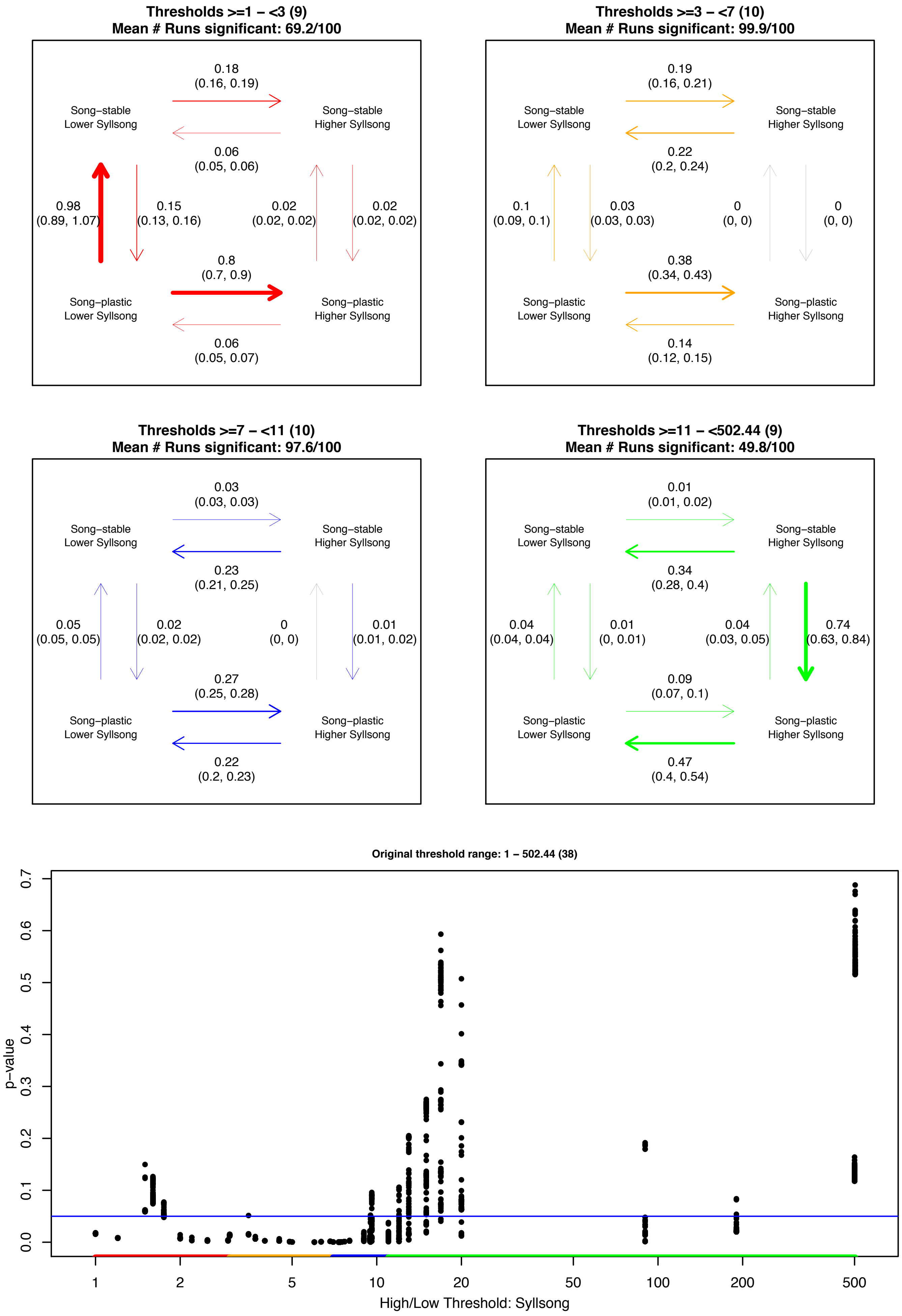

Thresholds >=1 – <3 (9)
Mean # Runs significant: 69.2/100
Song–stable Lower Syllsong
Song–stable Higher Syllsong
Song–plastic Lower Syllsong
Song–plastic Higher Syllsong
0.18 (0.16, 0.19)
0.06 (0.05, 0.06)
0.98 (0.89, 1.07)
0.15 (0.13, 0.16)
0.02 (0.02, 0.02)
0.02 (0.02, 0.02)
0.8 (0.7, 0.9)
0.06 (0.05, 0.07)
Thresholds >=3 – <7 (10)
Mean # Runs significant: 99.9/100
Song–stable Lower Syllsong
Song–stable Higher Syllsong
Song–plastic Lower Syllsong
Song–plastic Higher Syllsong
0.19 (0.16, 0.21)
0.22 (0.2, 0.24)
0.1 (0.09, 0.1)
0.03 (0.03, 0.03)
0 (0, 0)
0 (0, 0)
0.38 (0.34, 0.43)
0.14 (0.12, 0.15)
Thresholds >=7 – <11 (10)
Mean # Runs significant: 97.6/100
Song–stable Lower Syllsong
Song–stable Higher Syllsong
Song–plastic Lower Syllsong
Song–plastic Higher Syllsong
0.03 (0.03, 0.03)
0.23 (0.21, 0.25)
0.05 (0.05, 0.05)
0.02 (0.02, 0.02)
0 (0, 0)
0.01 (0.01, 0.02)
0.27 (0.25, 0.28)
0.22 (0.2, 0.23)
Thresholds >=11 – <502.44 (9)
Mean # Runs significant: 49.8/100
Song–stable Lower Syllsong
Song–stable Higher Syllsong
Song–plastic Lower Syllsong
Song–plastic Higher Syllsong
0.01 (0.01, 0.02)
0.34 (0.28, 0.4)
0.04 (0.04, 0.04)
0.01 (0, 0.01)
0.04 (0.03, 0.05)
0.74 (0.63, 0.84)
0.09 (0.07, 0.1)
0.47 (0.4, 0.54)
Original threshold range: 1 – 502.44 (38)
p–value
0.0
0.1
0.2
0.3
0.4
0.5
0.6
0.7
1
2
5
10
20
50
100
200
500
High/Low Threshold: Syllsong

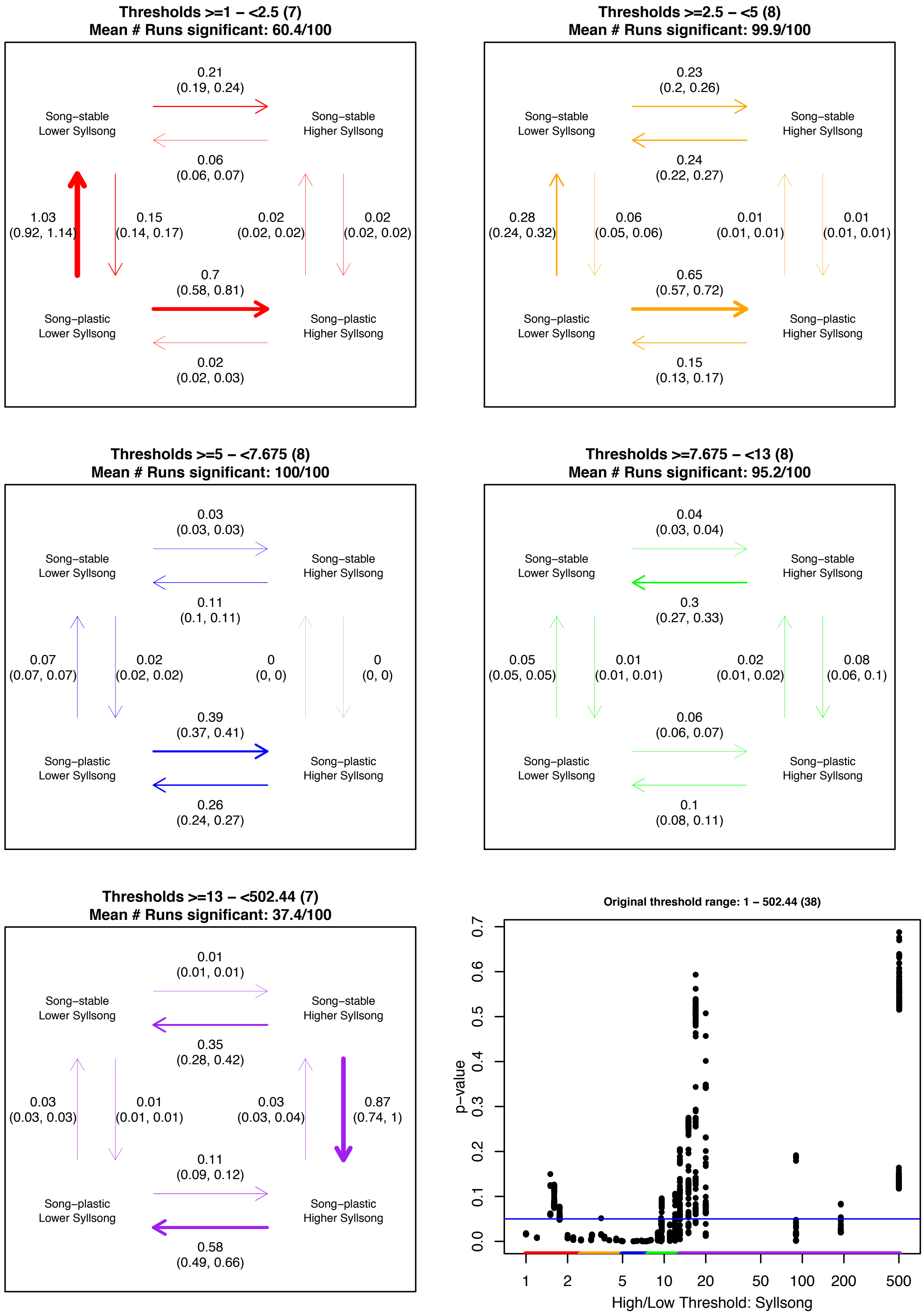
Thresholds >=1 – <2.5 (7)
Mean # Runs significant: 60.4/100
0.21
(0.19, 0.24)
Song–stable
Lower Syllsong
Song–stable
Higher Syllsong
0.06
(0.06, 0.07)
1.03
(0.92, 1.14)
0.15
(0.14, 0.17)
0.02
(0.02, 0.02)
0.02
(0.02, 0.02)
0.7
(0.58, 0.81)
Song–plastic
Lower Syllsong
Song–plastic
Higher Syllsong
0.02
(0.02, 0.03)
Thresholds >=2.5 – <5 (8)
Mean # Runs significant: 99.9/100
0.23
(0.2, 0.26)
Song–stable
Lower Syllsong
Song–stable
Higher Syllsong
0.24
(0.22, 0.27)
0.28
(0.24, 0.32)
0.06
(0.05, 0.06)
0.01
(0.01, 0.01)
0.01
(0.01, 0.01)
0.65
(0.57, 0.72)
Song–plastic
Lower Syllsong
Song–plastic
Higher Syllsong
0.15
(0.13, 0.17)
Thresholds >=5 – <7.675 (8)
Mean # Runs significant: 100/100
0.03
(0.03, 0.03)
Song–stable
Lower Syllsong
Song–stable
Higher Syllsong
0.11
(0.1, 0.11)
0.07
(0.07, 0.07)
0.02
(0.02, 0.02)
0
(0, 0)
0
(0, 0)
0.39
(0.37, 0.41)
Song–plastic
Lower Syllsong
Song–plastic
Higher Syllsong
0.26
(0.24, 0.27)
Thresholds >=7.675 – <13 (8)
Mean # Runs significant: 95.2/100
0.04
(0.03, 0.04)
Song–stable
Lower Syllsong
Song–stable
Higher Syllsong
0.3
(0.27, 0.33)
0.05
(0.05, 0.05)
0.01
(0.01, 0.01)
0.02
(0.01, 0.02)
0.08
(0.06, 0.1)
0.06
(0.06, 0.07)
Song–plastic
Lower Syllsong
Song–plastic
Higher Syllsong
0.1
(0.08, 0.11)
Thresholds >=13 – <502.44 (7)
Mean # Runs significant: 37.4/100
0.01
(0.01, 0.01)
Song–stable
Lower Syllsong
Song–stable
Higher Syllsong
0.35
(0.28, 0.42)
0.03
(0.03, 0.03)
0.01
(0.01, 0.01)
0.03
(0.03, 0.04)
0.87
(0.74, 1)
0.11
(0.09, 0.12)
Song–plastic
Lower Syllsong
Song–plastic
Higher Syllsong
0.58
(0.49, 0.66)
Original threshold range: 1 – 502.44 (38)
p–value
0.0
0.1
0.2
0.3
0.4
0.5
0.6
0.7
1
2
5
10
20
50
100
200
500
High/Low Threshold: Syllsong

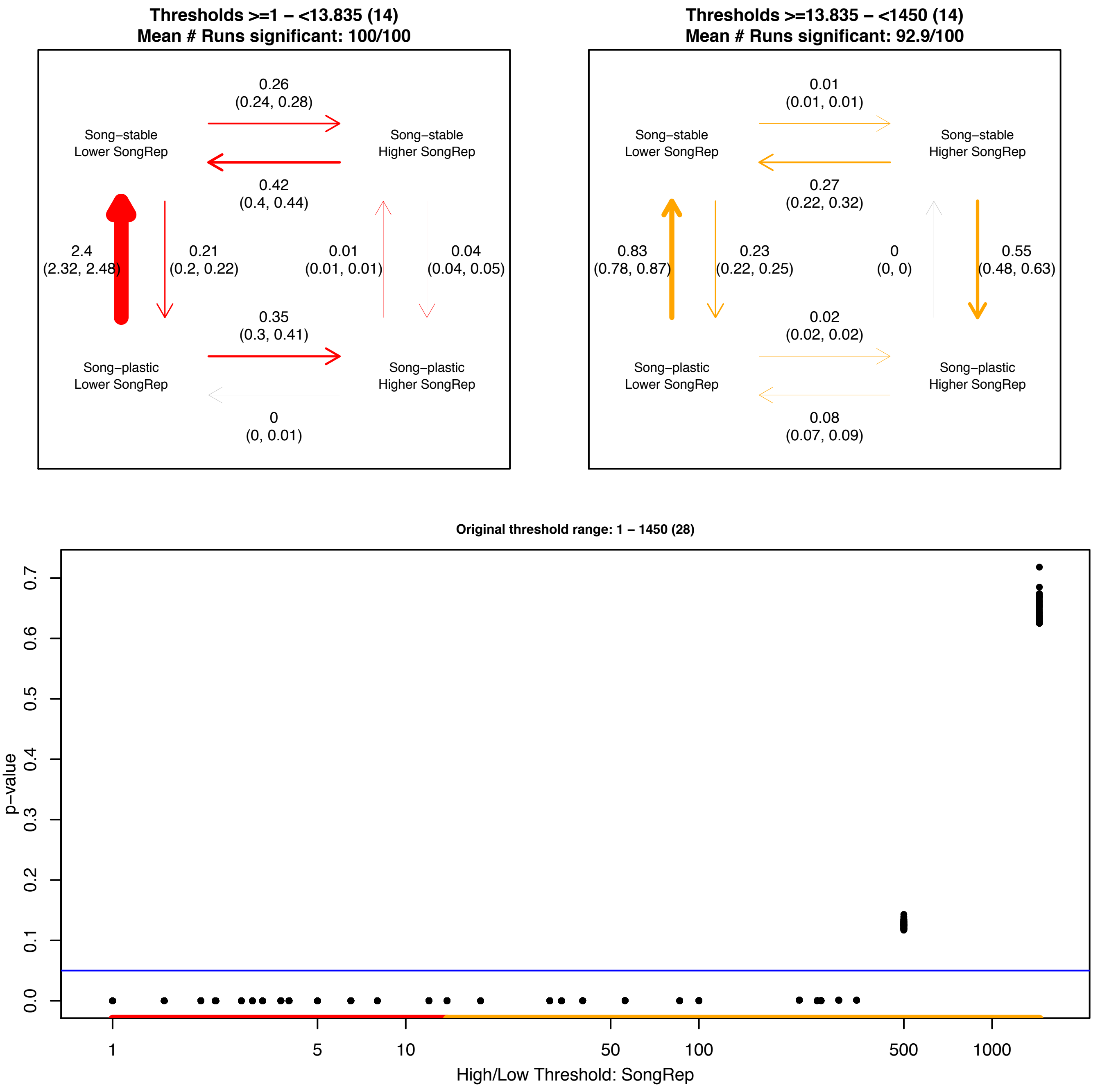

Thresholds >=1 – <13.835 (14)
Mean # Runs significant: 100/100
Song–stable Lower SongRep
Song–stable Higher SongRep
Song–plastic Lower SongRep
Song–plastic Higher SongRep
0.26 (0.24, 0.28)
0.42 (0.4, 0.44)
2.4 (2.32, 2.48)
0.21 (0.2, 0.22)
0.01 (0.01, 0.01)
0.04 (0.04, 0.05)
0.35 (0.3, 0.41)
0 (0, 0.01)
Thresholds >=13.835 – <1450 (14)
Mean # Runs significant: 92.9/100
0.01 (0.01, 0.01)
0.27 (0.22, 0.32)
0.83 (0.78, 0.87)
0.23 (0.22, 0.25)
0 (0, 0)
0.55 (0.48, 0.63)
0.02 (0.02, 0.02)
0.08 (0.07, 0.09)
Original threshold range: 1 – 1450 (28)
p–value
High/Low Threshold: SongRep

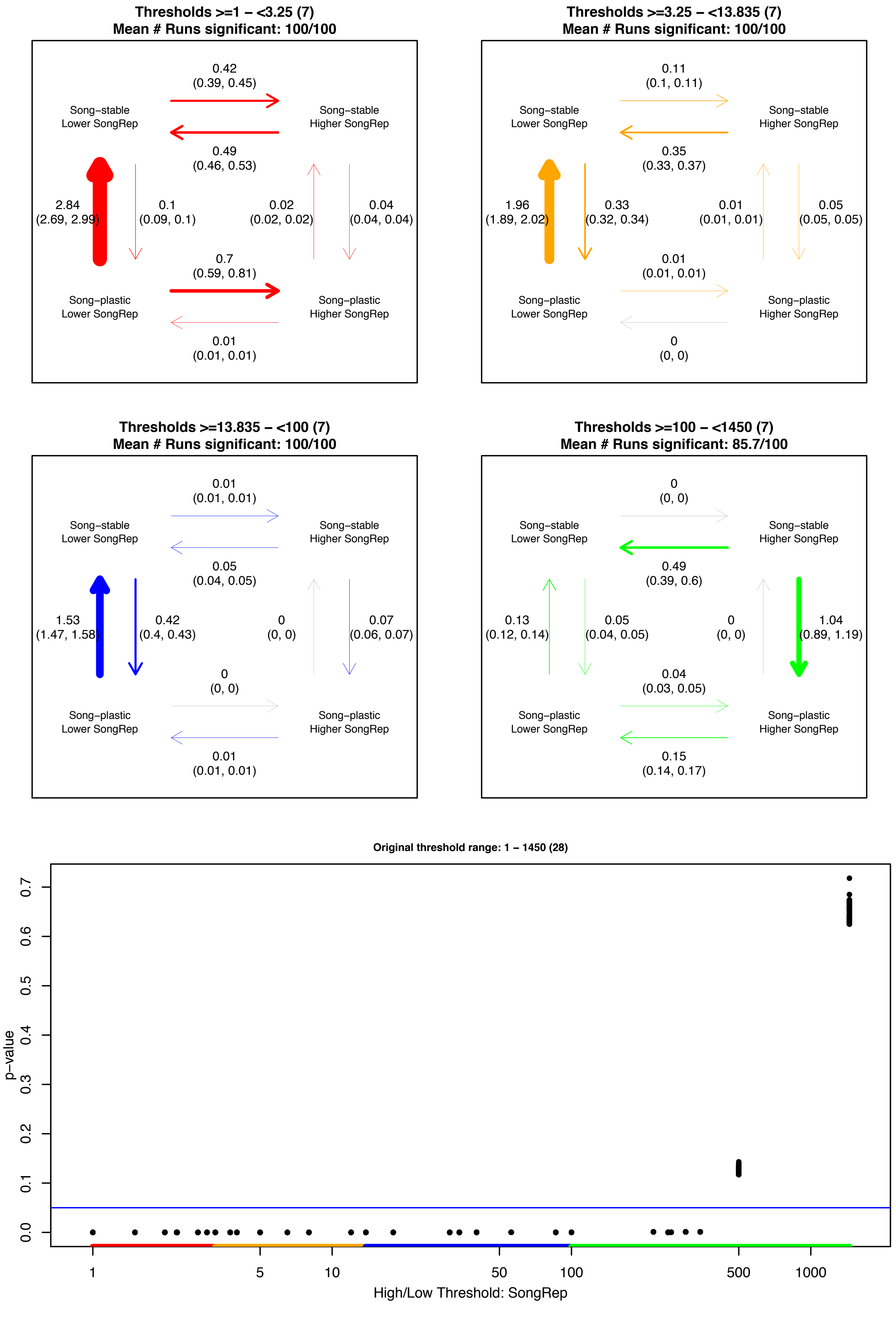
Thresholds >=1 – <3.25 (7)
Mean # Runs significant: 100/100
Song–stable Lower SongRep
Song–stable Higher SongRep
Song–plastic Lower SongRep
Song–plastic Higher SongRep
0.42 (0.39, 0.45)
0.49 (0.46, 0.53)
2.84 (2.69, 2.99)
0.1 (0.09, 0.1)
0.02 (0.02, 0.02)
0.04 (0.04, 0.04)
0.7 (0.59, 0.81)
0.01 (0.01, 0.01)
Thresholds >=3.25 – <13.835 (7)
Mean # Runs significant: 100/100
Song–stable Lower SongRep
Song–stable Higher SongRep
Song–plastic Lower SongRep
Song–plastic Higher SongRep
0.11 (0.1, 0.11)
0.35 (0.33, 0.37)
1.96 (1.89, 2.02)
0.33 (0.32, 0.34)
0.01 (0.01, 0.01)
0.05 (0.05, 0.05)
0.01 (0.01, 0.01)
0 (0, 0)
Thresholds >=13.835 – <100 (7)
Mean # Runs significant: 100/100
Song–stable Lower SongRep
Song–stable Higher SongRep
Song–plastic Lower SongRep
Song–plastic Higher SongRep
0.01 (0.01, 0.01)
0.05 (0.04, 0.05)
1.53 (1.47, 1.58)
0.42 (0.4, 0.43)
0 (0, 0)
0.07 (0.06, 0.07)
0 (0, 0)
0.01 (0.01, 0.01)
Thresholds >=100 – <1450 (7)
Mean # Runs significant: 85.7/100
Song–stable Lower SongRep
Song–stable Higher SongRep
Song–plastic Lower SongRep
Song–plastic Higher SongRep
0 (0, 0)
0.49 (0.39, 0.6)
0.13 (0.12, 0.14)
0.05 (0.04, 0.05)
0 (0, 0)
1.04 (0.89, 1.19)
0.04 (0.03, 0.05)
0.15 (0.14, 0.17)
Original threshold range: 1 – 1450 (28)
p–value
0.0
0.1
0.2
0.3
0.4
0.5
0.6
0.7
1
5
10
50
100
500
1000
High/Low Threshold: SongRep

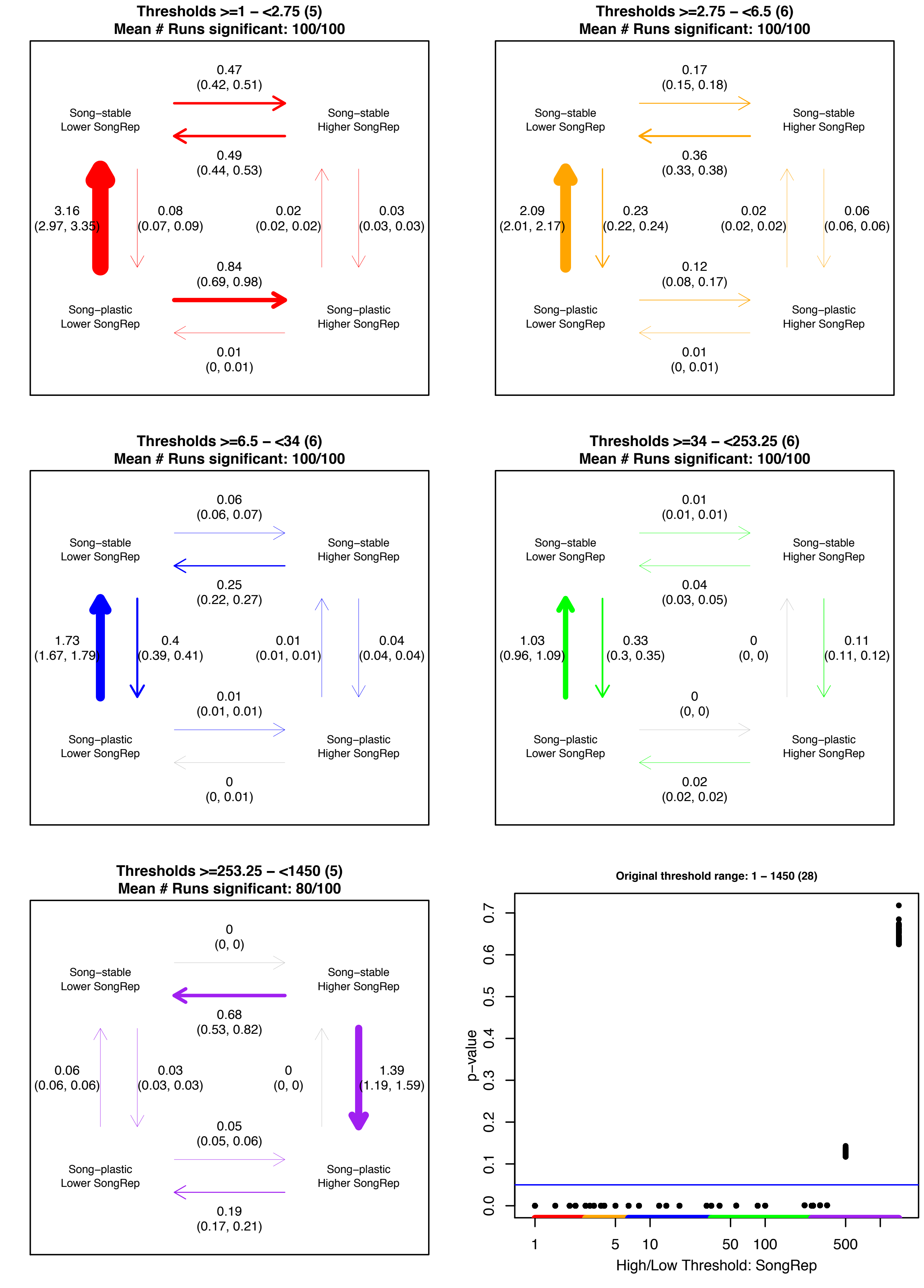

Thresholds >=1 – <2.75 (5)
Mean # Runs significant: 100/100
0.47
(0.42, 0.51)
Song–stable
Lower SongRep
Song–stable
Higher SongRep
0.49
(0.44, 0.53)
3.16
(2.97, 3.35)
0.08
(0.07, 0.09)
0.02
(0.02, 0.02)
0.03
(0.03, 0.03)
0.84
(0.69, 0.98)
Song–plastic
Lower SongRep
Song–plastic
Higher SongRep
0.01
(0, 0.01)
Thresholds >=2.75 – <6.5 (6)
Mean # Runs significant: 100/100
0.17
(0.15, 0.18)
Song–stable
Lower SongRep
Song–stable
Higher SongRep
0.36
(0.33, 0.38)
2.09
(2.01, 2.17)
0.23
(0.22, 0.24)
0.02
(0.02, 0.02)
0.06
(0.06, 0.06)
0.12
(0.08, 0.17)
Song–plastic
Lower SongRep
Song–plastic
Higher SongRep
0.01
(0, 0.01)
Thresholds >=6.5 – <34 (6)
Mean # Runs significant: 100/100
0.06
(0.06, 0.07)
Song–stable
Lower SongRep
Song–stable
Higher SongRep
0.25
(0.22, 0.27)
1.73
(1.67, 1.79)
0.4
(0.39, 0.41)
0.01
(0.01, 0.01)
0.04
(0.04, 0.04)
0.01
(0.01, 0.01)
Song–plastic
Lower SongRep
Song–plastic
Higher SongRep
0
(0, 0.01)
Thresholds >=34 – <253.25 (6)
Mean # Runs significant: 100/100
0.01
(0.01, 0.01)
Song–stable
Lower SongRep
Song–stable
Higher SongRep
0.04
(0.03, 0.05)
1.03
(0.96, 1.09)
0.33
(0.3, 0.35)
0
(0, 0)
0.11
(0.11, 0.12)
0
(0, 0)
Song–plastic
Lower SongRep
Song–plastic
Higher SongRep
0.02
(0.02, 0.02)
Thresholds >=253.25 – <1450 (5)
Mean # Runs significant: 80/100
0
(0, 0)
Song–stable
Lower SongRep
Song–stable
Higher SongRep
0.68
(0.53, 0.82)
0.06
(0.06, 0.06)
0.03
(0.03, 0.03)
0
(0, 0)
1.39
(1.19, 1.59)
0.05
(0.05, 0.06)
Song–plastic
Lower SongRep
Song–plastic
Higher SongRep
0.19
(0.17, 0.21)
Original threshold range: 1 – 1450 (28)
p–value
0.0
0.1
0.2
0.3
0.4
0.5
0.6
0.7
1
5
10
50
100
500
High/Low Threshold: SongRep

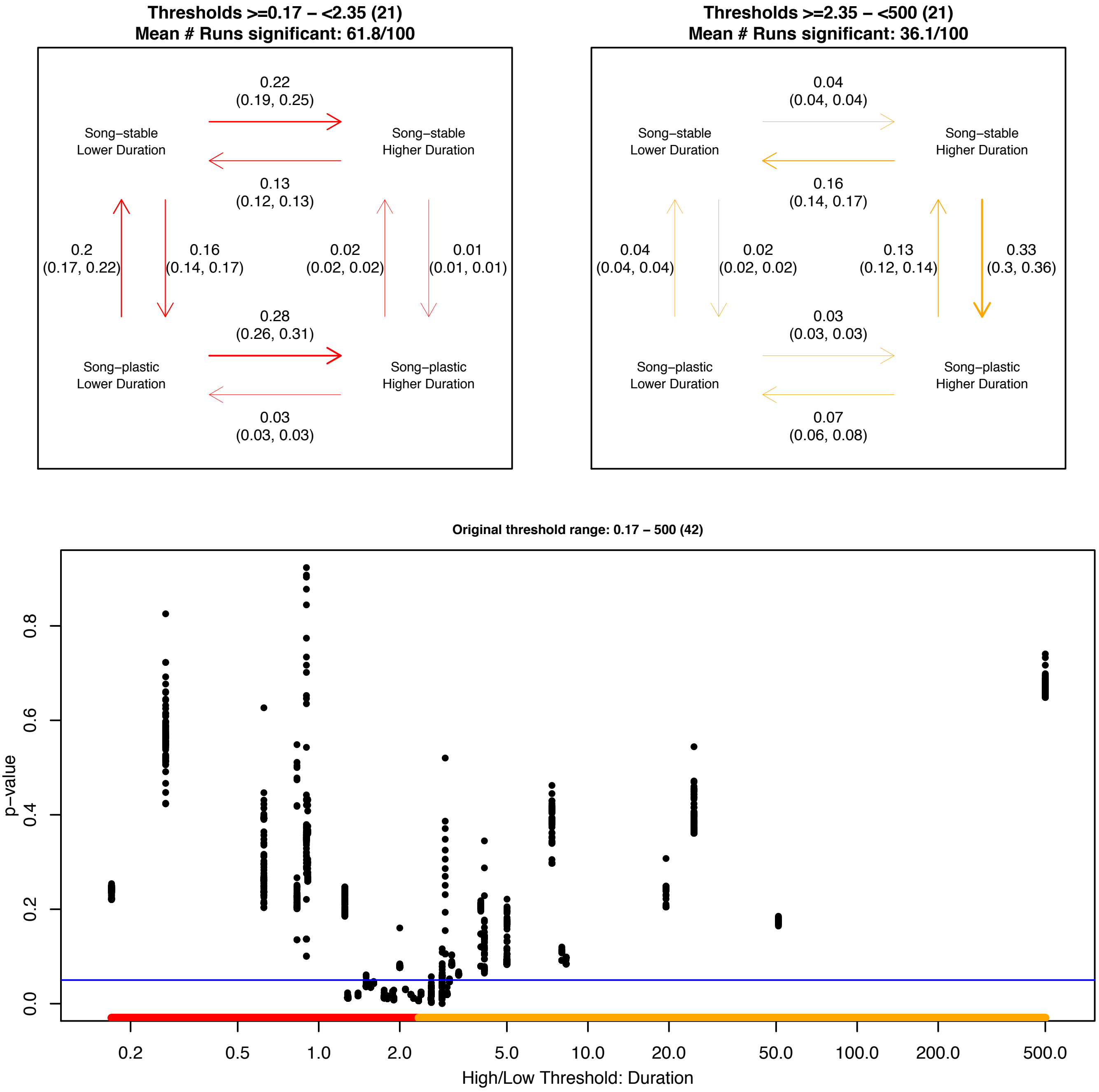

Thresholds >=0.17 – <2.35 (21)
Mean # Runs significant: 61.8/100
0.22
(0.19, 0.25)
Song−stable
Lower Duration
Song−stable
Higher Duration
0.13
(0.12, 0.13)
0.2
(0.17, 0.22)
0.16
(0.14, 0.17)
0.02
(0.02, 0.02)
0.01
(0.01, 0.01)
0.28
(0.26, 0.31)
Song−plastic
Lower Duration
Song−plastic
Higher Duration
0.03
(0.03, 0.03)
Thresholds >=2.35 – <500 (21)
Mean # Runs significant: 36.1/100
0.04
(0.04, 0.04)
Song−stable
Lower Duration
Song−stable
Higher Duration
0.16
(0.14, 0.17)
0.04
(0.04, 0.04)
0.02
(0.02, 0.02)
0.13
(0.12, 0.14)
0.33
(0.3, 0.36)
0.03
(0.03, 0.03)
Song−plastic
Lower Duration
Song−plastic
Higher Duration
0.07
(0.06, 0.08)
Original threshold range: 0.17 – 500 (42)
p−value
0.0
0.2
0.4
0.6
0.8
0.2
0.5
1.0
2.0
5.0
10.0
20.0
50.0
100.0
200.0
500.0
High/Low Threshold: Duration

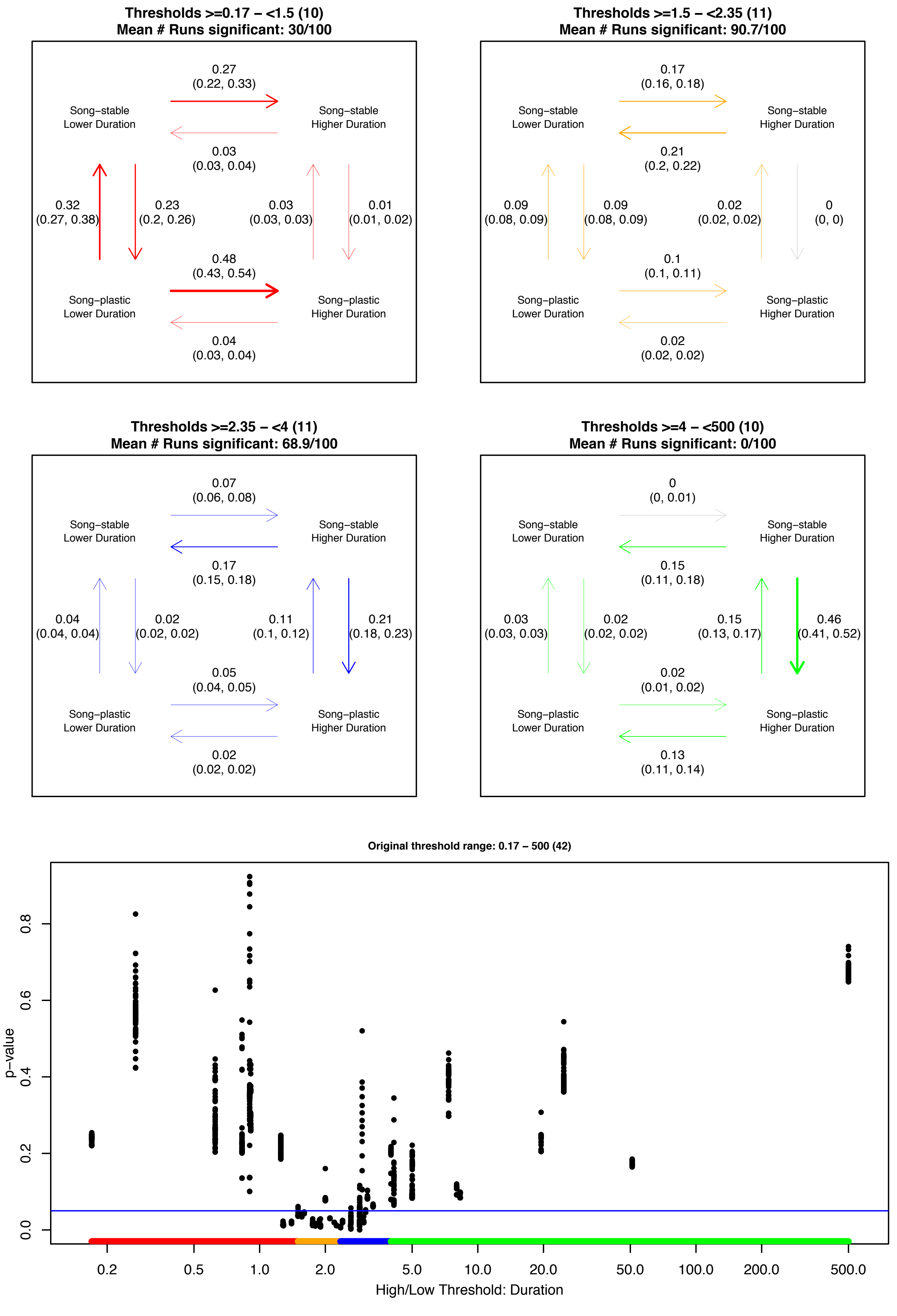
Thresholds >=0.17 – <1.5 (10)
Mean # Runs significant: 30/100
Song–stable Lower Duration
Song–stable Higher Duration
Song–plastic Lower Duration
Song–plastic Higher Duration
0.27 (0.22, 0.33)
0.03 (0.03, 0.04)
0.32 (0.27, 0.38)
0.23 (0.2, 0.26)
0.03 (0.03, 0.03)
0.01 (0.01, 0.02)
0.48 (0.43, 0.54)
0.04 (0.03, 0.04)
Thresholds >=1.5 – <2.35 (11)
Mean # Runs significant: 90.7/100
Song–stable Lower Duration
Song–stable Higher Duration
Song–plastic Lower Duration
Song–plastic Higher Duration
0.17 (0.16, 0.18)
0.21 (0.2, 0.22)
0.09 (0.08, 0.09)
0.09 (0.08, 0.09)
0.02 (0.02, 0.02)
0 (0, 0)
0.1 (0.1, 0.11)
0.02 (0.02, 0.02)
Thresholds >=2.35 – <4 (11)
Mean # Runs significant: 68.9/100
Song–stable Lower Duration
Song–stable Higher Duration
Song–plastic Lower Duration
Song–plastic Higher Duration
0.07 (0.06, 0.08)
0.17 (0.15, 0.18)
0.04 (0.04, 0.04)
0.02 (0.02, 0.02)
0.11 (0.1, 0.12)
0.21 (0.18, 0.23)
0.05 (0.04, 0.05)
0.02 (0.02, 0.02)
Thresholds >=4 – <500 (10)
Mean # Runs significant: 0/100
Song–stable Lower Duration
Song–stable Higher Duration
Song–plastic Lower Duration
Song–plastic Higher Duration
0 (0, 0.01)
0.15 (0.11, 0.18)
0.03 (0.03, 0.03)
0.02 (0.02, 0.02)
0.15 (0.13, 0.17)
0.46 (0.41, 0.52)
0.02 (0.01, 0.02)
0.13 (0.11, 0.14)
Original threshold range: 0.17 – 500 (42)
p–value
0.0
0.2
0.4
0.6
0.8
0.2
0.5
1.0
2.0
5.0
10.0
20.0
50.0
100.0
200.0
500.0
High/Low Threshold: Duration

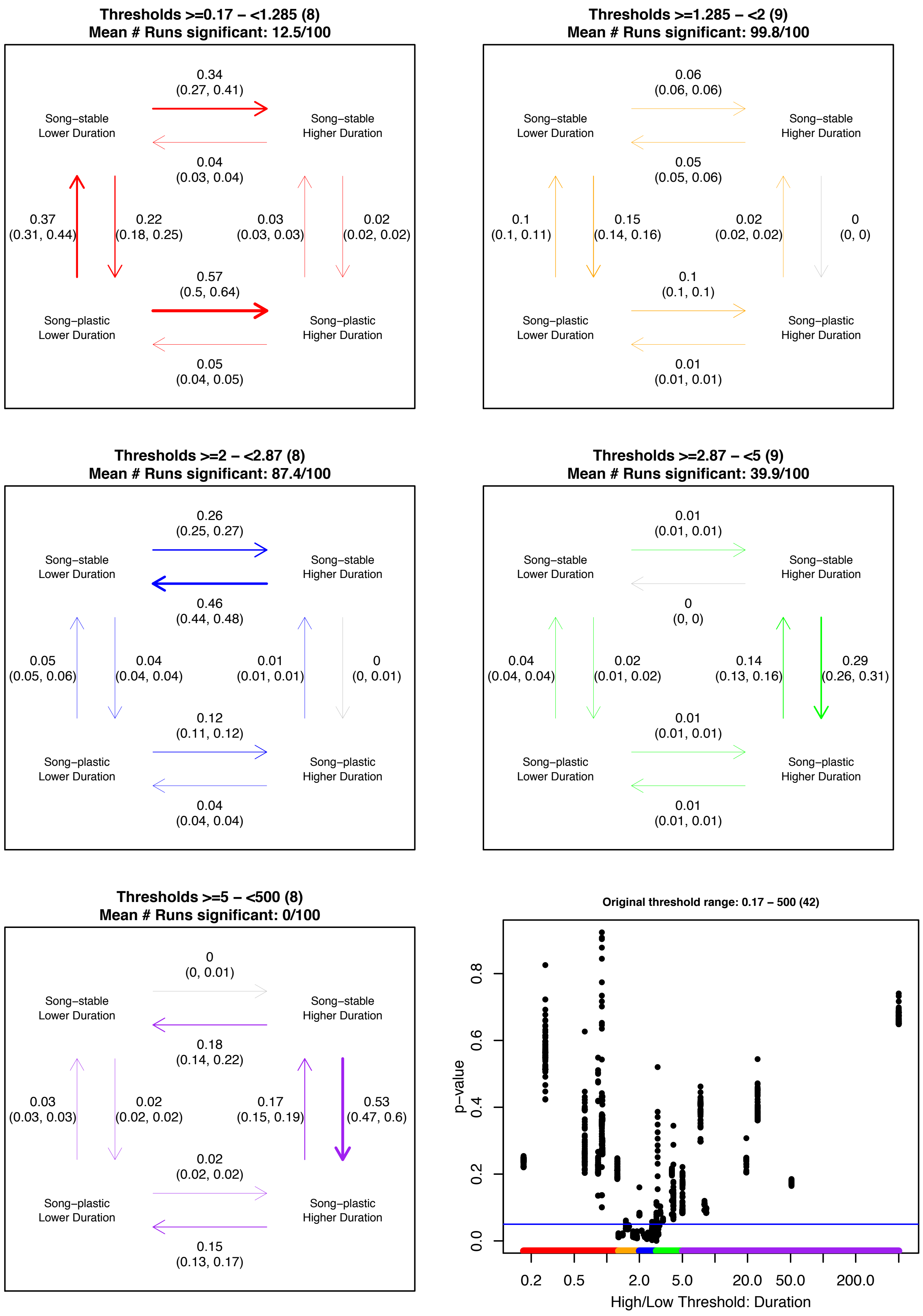

Thresholds >=0.17 – <1.285 (8)
Mean # Runs significant: 12.5/100
0.34
(0.27, 0.41)
Song−stable
Lower Duration
Song−stable
Higher Duration
0.04
(0.03, 0.04)
0.37
(0.31, 0.44)
0.22
(0.18, 0.25)
0.03
(0.03, 0.03)
0.02
(0.02, 0.02)
0.57
(0.5, 0.64)
Song−plastic
Lower Duration
Song−plastic
Higher Duration
0.05
(0.04, 0.05)
Thresholds >=1.285 – <2 (9)
Mean # Runs significant: 99.8/100
0.06
(0.06, 0.06)
Song−stable
Lower Duration
Song−stable
Higher Duration
0.05
(0.05, 0.06)
0.1
(0.1, 0.11)
0.15
(0.14, 0.16)
0.02
(0.02, 0.02)
0
(0, 0)
0.1
(0.1, 0.1)
Song−plastic
Lower Duration
Song−plastic
Higher Duration
0.01
(0.01, 0.01)
Thresholds >=2 – <2.87 (8)
Mean # Runs significant: 87.4/100
0.26
(0.25, 0.27)
Song−stable
Lower Duration
Song−stable
Higher Duration
0.46
(0.44, 0.48)
0.05
(0.05, 0.06)
0.04
(0.04, 0.04)
0.01
(0.01, 0.01)
0
(0, 0.01)
0.12
(0.11, 0.12)
Song−plastic
Lower Duration
Song−plastic
Higher Duration
0.04
(0.04, 0.04)
Thresholds >=2.87 – <5 (9)
Mean # Runs significant: 39.9/100
0.01
(0.01, 0.01)
Song−stable
Lower Duration
Song−stable
Higher Duration
0
(0, 0)
0.04
(0.04, 0.04)
0.02
(0.01, 0.02)
0.14
(0.13, 0.16)
0.29
(0.26, 0.31)
0.01
(0.01, 0.01)
Song−plastic
Lower Duration
Song−plastic
Higher Duration
0.01
(0.01, 0.01)
Thresholds >=5 – <500 (8)
Mean # Runs significant: 0/100
0
(0, 0.01)
Song−stable
Lower Duration
Song−stable
Higher Duration
0.18
(0.14, 0.22)
0.03
(0.03, 0.03)
0.02
(0.02, 0.02)
0.17
(0.15, 0.19)
0.53
(0.47, 0.6)
0.02
(0.02, 0.02)
Song−plastic
Lower Duration
Song−plastic
Higher Duration
0.15
(0.13, 0.17)
Original threshold range: 0.17 – 500 (42)
p−value
0.0
0.2
0.4
0.6
0.8
0.2
0.5
2.0
5.0
20.0
50.0
200.0
High/Low Threshold: Duration

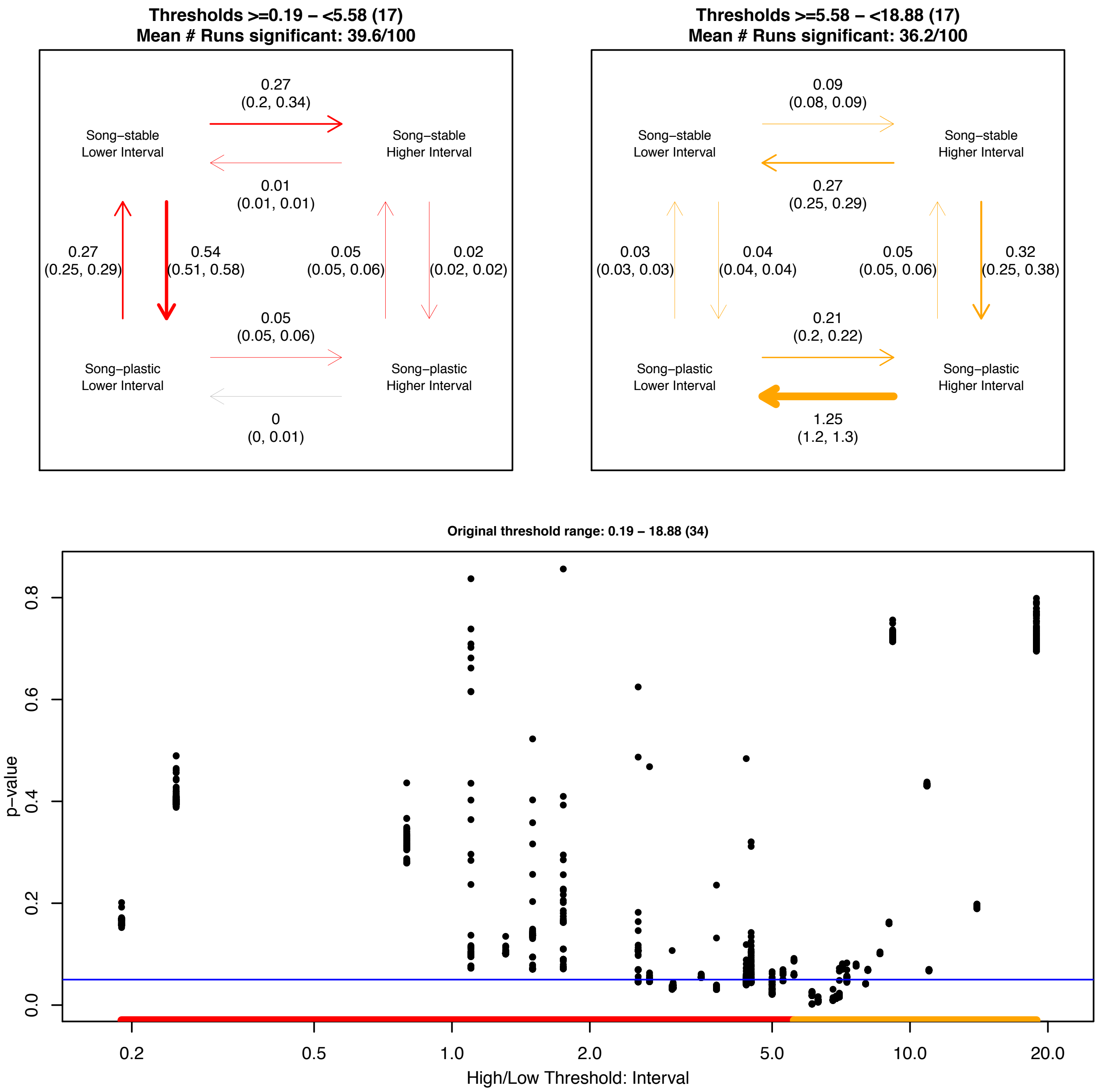
Thresholds >=0.19 – <5.58 (17)
Mean # Runs significant: 39.6/100
Song–stable Lower Interval
Song–stable Higher Interval
Song–plastic Lower Interval
Song–plastic Higher Interval
0.27 (0.2, 0.34)
0.01 (0.01, 0.01)
0.27 (0.25, 0.29)
0.54 (0.51, 0.58)
0.05 (0.05, 0.06)
0.02 (0.02, 0.02)
0.05 (0.05, 0.06)
0 (0, 0.01)
Thresholds >=5.58 – <18.88 (17)
Mean # Runs significant: 36.2/100
0.09 (0.08, 0.09)
0.27 (0.25, 0.29)
0.03 (0.03, 0.03)
0.04 (0.04, 0.04)
0.05 (0.05, 0.06)
0.32 (0.25, 0.38)
0.21 (0.2, 0.22)
1.25 (1.2, 1.3)
Original threshold range: 0.19 – 18.88 (34)
p–value
0.0
0.2
0.4
0.6
0.8
0.2
0.5
1.0
2.0
5.0
10.0
20.0
High/Low Threshold: Interval

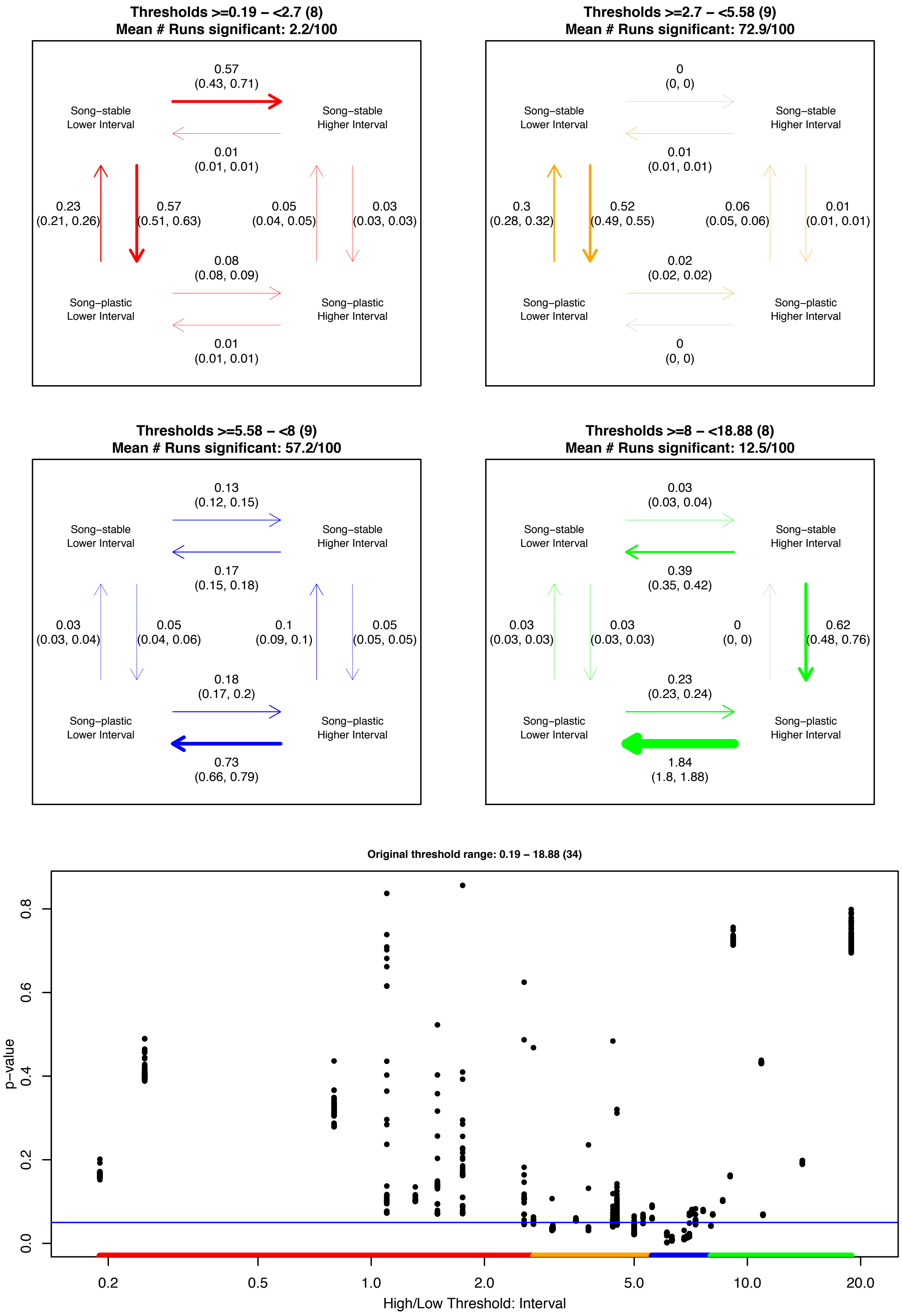
Thresholds >=0.19 – <2.7 (8)
Mean # Runs significant: 2.2/100
Song−stable Lower Interval
Song−stable Higher Interval
Song−plastic Lower Interval
Song−plastic Higher Interval
0.57 (0.43, 0.71)
0.01 (0.01, 0.01)
0.23 (0.21, 0.26)
0.57 (0.51, 0.63)
0.05 (0.04, 0.05)
0.03 (0.03, 0.03)
0.08 (0.08, 0.09)
0.01 (0.01, 0.01)
Thresholds >=2.7 – <5.58 (9)
Mean # Runs significant: 72.9/100
0 (0, 0)
0.01 (0.01, 0.01)
0.3 (0.28, 0.32)
0.52 (0.49, 0.55)
0.06 (0.05, 0.06)
0.01 (0.01, 0.01)
0.02 (0.02, 0.02)
0 (0, 0)
Thresholds >=5.58 – <8 (9)
Mean # Runs significant: 57.2/100
0.13 (0.12, 0.15)
0.17 (0.15, 0.18)
0.03 (0.03, 0.04)
0.05 (0.04, 0.06)
0.1 (0.09, 0.1)
0.05 (0.05, 0.05)
0.18 (0.17, 0.2)
0.73 (0.66, 0.79)
Thresholds >=8 – <18.88 (8)
Mean # Runs significant: 12.5/100
0.03 (0.03, 0.04)
0.39 (0.35, 0.42)
0.03 (0.03, 0.03)
0.03 (0.03, 0.03)
0 (0, 0)
0.62 (0.48, 0.76)
0.23 (0.23, 0.24)
1.84 (1.8, 1.88)
Original threshold range: 0.19 – 18.88 (34)
p−value
0.0
0.2
0.4
0.6
0.8
0.2
0.5
1.0
2.0
5.0
10.0
20.0
High/Low Threshold: Interval

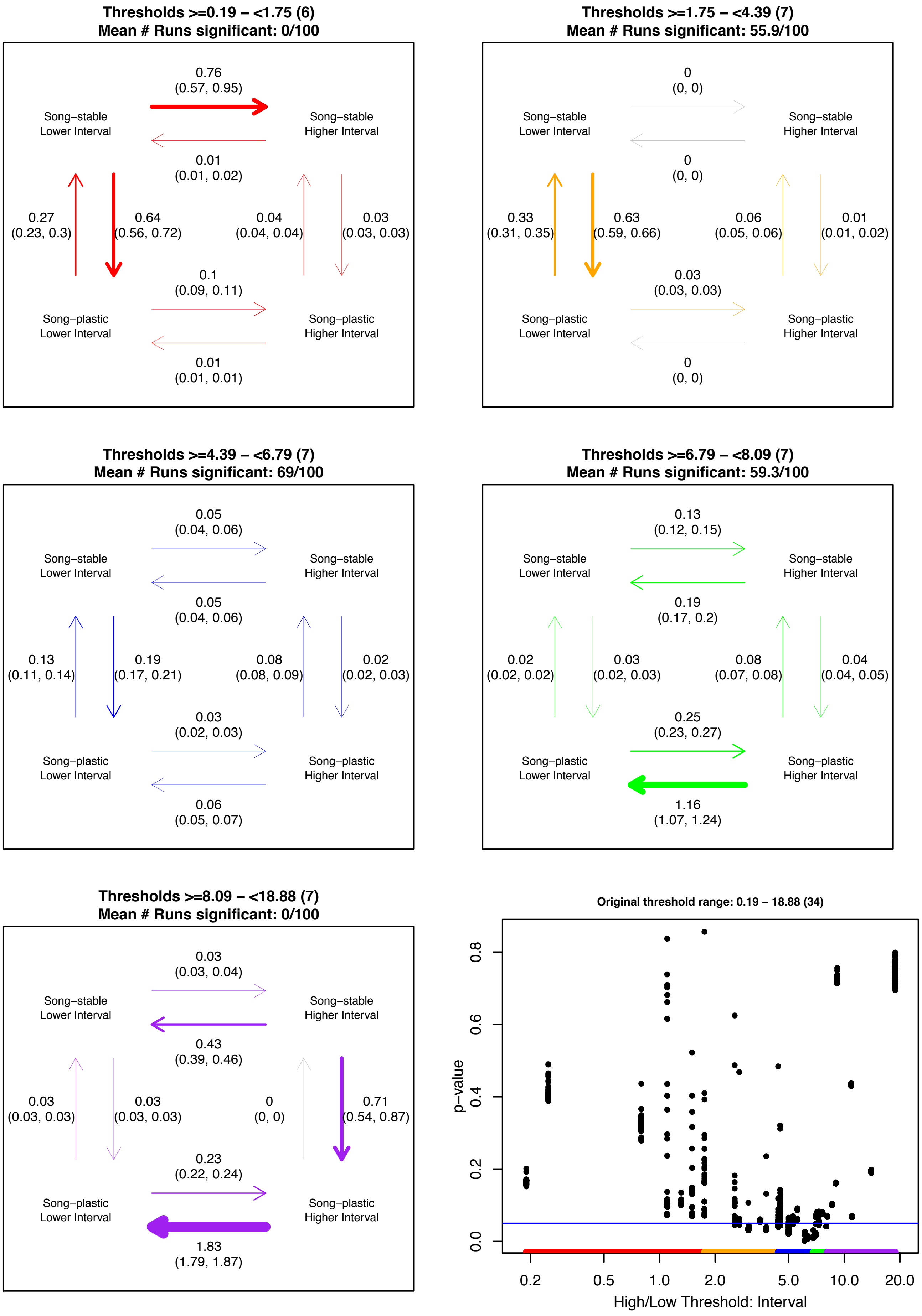
Thresholds >=0.19 – <1.75 (6)
Mean # Runs significant: 0/100
Song–stable Lower Interval
Song–stable Higher Interval
Song–plastic Lower Interval
Song–plastic Higher Interval
0.76 (0.57, 0.95)
0.01 (0.01, 0.02)
0.27 (0.23, 0.3)
0.64 (0.56, 0.72)
0.04 (0.04, 0.04)
0.03 (0.03, 0.03)
0.1 (0.09, 0.11)
0.01 (0.01, 0.01)
Thresholds >=1.75 – <4.39 (7)
Mean # Runs significant: 55.9/100
Song–stable Lower Interval
Song–stable Higher Interval
Song–plastic Lower Interval
Song–plastic Higher Interval
0 (0, 0)
0 (0, 0)
0.33 (0.31, 0.35)
0.63 (0.59, 0.66)
0.06 (0.05, 0.06)
0.01 (0.01, 0.02)
0.03 (0.03, 0.03)
0 (0, 0)
Thresholds >=4.39 – <6.79 (7)
Mean # Runs significant: 69/100
Song–stable Lower Interval
Song–stable Higher Interval
Song–plastic Lower Interval
Song–plastic Higher Interval
0.05 (0.04, 0.06)
0.05 (0.04, 0.06)
0.13 (0.11, 0.14)
0.19 (0.17, 0.21)
0.08 (0.08, 0.09)
0.02 (0.02, 0.03)
0.03 (0.02, 0.03)
0.06 (0.05, 0.07)
Thresholds >=6.79 – <8.09 (7)
Mean # Runs significant: 59.3/100
Song–stable Lower Interval
Song–stable Higher Interval
Song–plastic Lower Interval
Song–plastic Higher Interval
0.13 (0.12, 0.15)
0.19 (0.17, 0.2)
0.02 (0.02, 0.02)
0.03 (0.02, 0.03)
0.08 (0.07, 0.08)
0.04 (0.04, 0.05)
0.25 (0.23, 0.27)
1.16 (1.07, 1.24)
Thresholds >=8.09 – <18.88 (7)
Mean # Runs significant: 0/100
Song–stable Lower Interval
Song–stable Higher Interval
Song–plastic Lower Interval
Song–plastic Higher Interval
0.03 (0.03, 0.04)
0.43 (0.39, 0.46)
0.03 (0.03, 0.03)
0.03 (0.03, 0.03)
0 (0, 0)
0.71 (0.54, 0.87)
0.23 (0.22, 0.24)
1.83 (1.79, 1.87)
Original threshold range: 0.19 – 18.88 (34)
p–value
0.0
0.2
0.4
0.6
0.8
0.2
0.5
1.0
2.0
5.0
10.0
20.0
High/Low Threshold: Interval

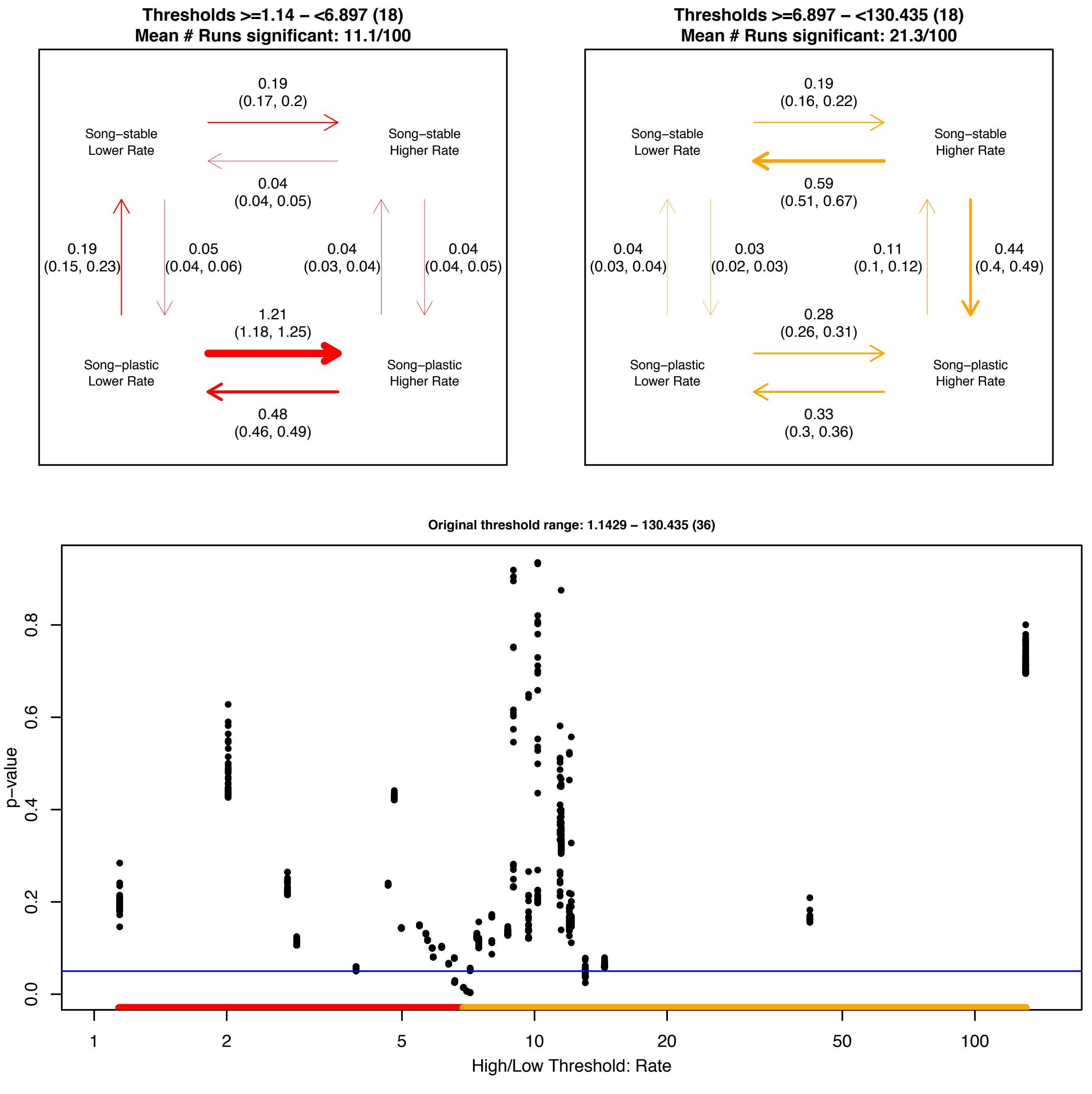
Thresholds >=1.14 – <6.897 (18)
Mean # Runs significant: 11.1/100
Song–stable Lower Rate
Song–stable Higher Rate
Song–plastic Lower Rate
Song–plastic Higher Rate
0.19 (0.17, 0.2)
0.04 (0.04, 0.05)
0.19 (0.15, 0.23)
0.05 (0.04, 0.06)
0.04 (0.03, 0.04)
0.04 (0.04, 0.05)
1.21 (1.18, 1.25)
0.48 (0.46, 0.49)
Thresholds >=6.897 – <130.435 (18)
Mean # Runs significant: 21.3/100
Song–stable Lower Rate
Song–stable Higher Rate
Song–plastic Lower Rate
Song–plastic Higher Rate
0.19 (0.16, 0.22)
0.59 (0.51, 0.67)
0.04 (0.03, 0.04)
0.03 (0.02, 0.03)
0.11 (0.1, 0.12)
0.44 (0.4, 0.49)
0.28 (0.26, 0.31)
0.33 (0.3, 0.36)
Original threshold range: 1.1429 – 130.435 (36)
p–value
0.0
0.2
0.4
0.6
0.8
1
2
5
10
20
50
100
High/Low Threshold: Rate

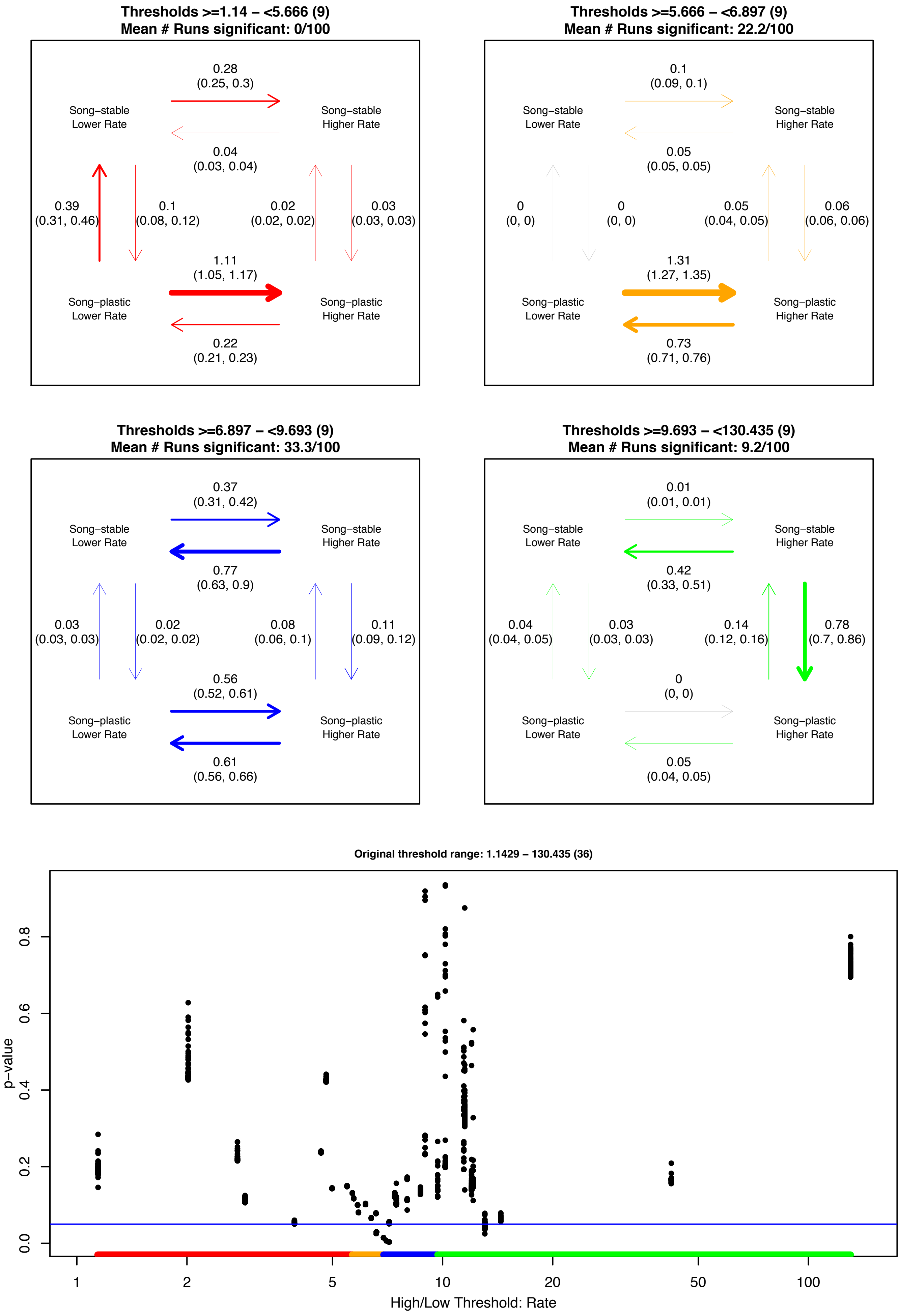

Thresholds >=1.14 – <5.666 (9)
Mean # Runs significant: 0/100
Song–stable Lower Rate
Song–stable Higher Rate
Song–plastic Lower Rate
Song–plastic Higher Rate
0.28 (0.25, 0.3)
0.04 (0.03, 0.04)
0.39 (0.31, 0.46)
0.1 (0.08, 0.12)
0.02 (0.02, 0.02)
0.03 (0.03, 0.03)
1.11 (1.05, 1.17)
0.22 (0.21, 0.23)
Thresholds >=5.666 – <6.897 (9)
Mean # Runs significant: 22.2/100
Song–stable Lower Rate
Song–stable Higher Rate
Song–plastic Lower Rate
Song–plastic Higher Rate
0.1 (0.09, 0.1)
0.05 (0.05, 0.05)
0 (0, 0)
0 (0, 0)
0.05 (0.04, 0.05)
0.06 (0.06, 0.06)
1.31 (1.27, 1.35)
0.73 (0.71, 0.76)
Thresholds >=6.897 – <9.693 (9)
Mean # Runs significant: 33.3/100
Song–stable Lower Rate
Song–stable Higher Rate
Song–plastic Lower Rate
Song–plastic Higher Rate
0.37 (0.31, 0.42)
0.77 (0.63, 0.9)
0.03 (0.03, 0.03)
0.02 (0.02, 0.02)
0.08 (0.06, 0.1)
0.11 (0.09, 0.12)
0.56 (0.52, 0.61)
0.61 (0.56, 0.66)
Thresholds >=9.693 – <130.435 (9)
Mean # Runs significant: 9.2/100
Song–stable Lower Rate
Song–stable Higher Rate
Song–plastic Lower Rate
Song–plastic Higher Rate
0.01 (0.01, 0.01)
0.42 (0.33, 0.51)
0.04 (0.04, 0.05)
0.03 (0.03, 0.03)
0.14 (0.12, 0.16)
0.78 (0.7, 0.86)
0 (0, 0)
0.05 (0.04, 0.05)
Original threshold range: 1.1429 – 130.435 (36)
p–value
0.0
0.2
0.4
0.6
0.8
1
2
5
10
20
50
100
High/Low Threshold: Rate

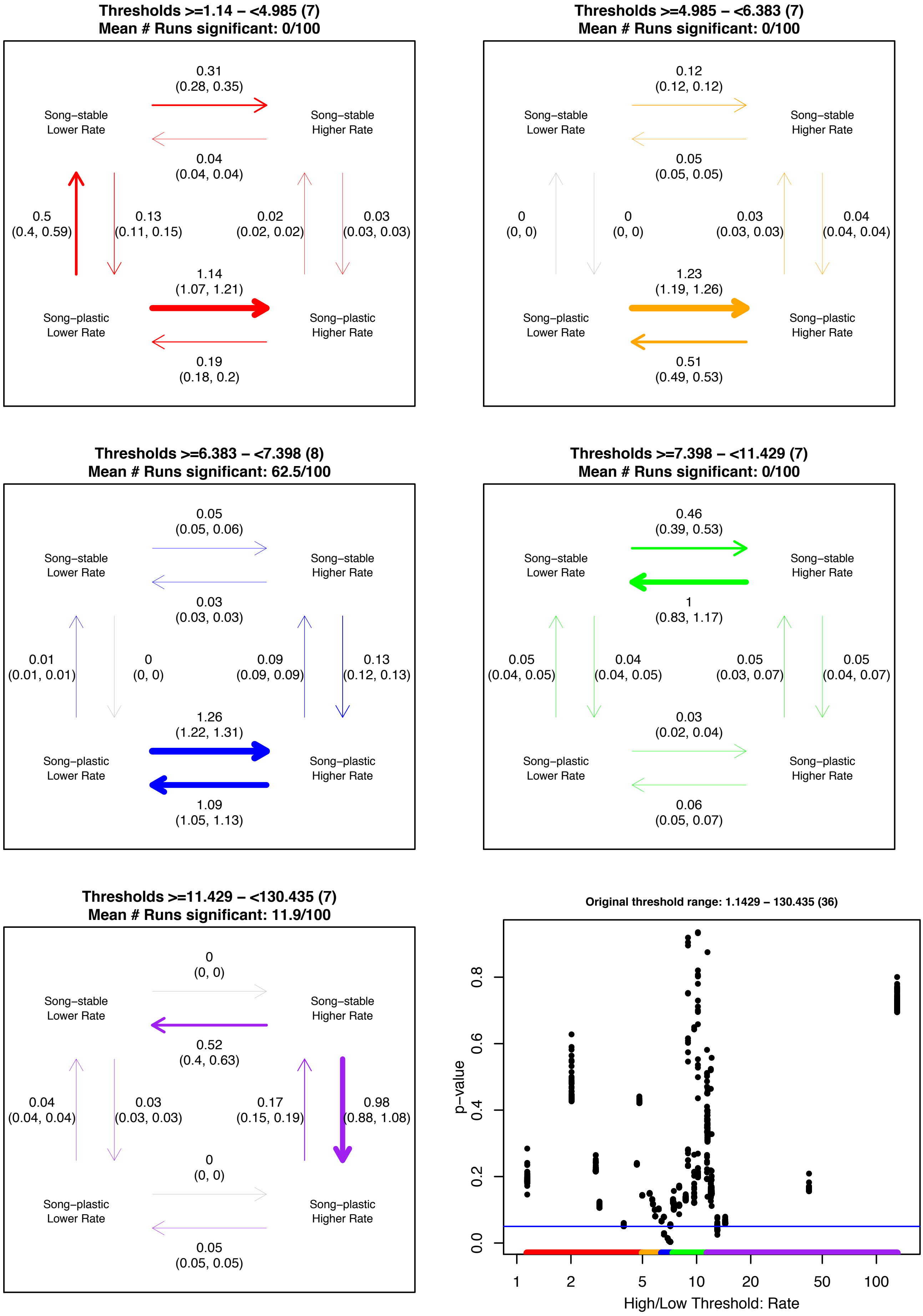
Thresholds >=1.14 – <4.985 (7)
Mean # Runs significant: 0/100
0.31
(0.28, 0.35)
Song–stable
Lower Rate
Song–stable
Higher Rate
0.04
(0.04, 0.04)
0.5
(0.4, 0.59)
0.13
(0.11, 0.15)
0.02
(0.02, 0.02)
0.03
(0.03, 0.03)
1.14
(1.07, 1.21)
Song–plastic
Lower Rate
Song–plastic
Higher Rate
0.19
(0.18, 0.2)
Thresholds >=4.985 – <6.383 (7)
Mean # Runs significant: 0/100
0.12
(0.12, 0.12)
Song–stable
Lower Rate
Song–stable
Higher Rate
0.05
(0.05, 0.05)
0
(0, 0)
0
(0, 0)
0.03
(0.03, 0.03)
0.04
(0.04, 0.04)
1.23
(1.19, 1.26)
Song–plastic
Lower Rate
Song–plastic
Higher Rate
0.51
(0.49, 0.53)
Thresholds >=6.383 – <7.398 (8)
Mean # Runs significant: 62.5/100
0.05
(0.05, 0.06)
Song–stable
Lower Rate
Song–stable
Higher Rate
0.03
(0.03, 0.03)
0.01
(0.01, 0.01)
0
(0, 0)
0.09
(0.09, 0.09)
0.13
(0.12, 0.13)
1.26
(1.22, 1.31)
Song–plastic
Lower Rate
Song–plastic
Higher Rate
1.09
(1.05, 1.13)
Thresholds >=7.398 – <11.429 (7)
Mean # Runs significant: 0/100
0.46
(0.39, 0.53)
Song–stable
Lower Rate
Song–stable
Higher Rate
1
(0.83, 1.17)
0.05
(0.04, 0.05)
0.04
(0.04, 0.05)
0.05
(0.03, 0.07)
0.05
(0.04, 0.07)
0.03
(0.02, 0.04)
Song–plastic
Lower Rate
Song–plastic
Higher Rate
0.06
(0.05, 0.07)
Thresholds >=11.429 – <130.435 (7)
Mean # Runs significant: 11.9/100
0
(0, 0)
Song–stable
Lower Rate
Song–stable
Higher Rate
0.52
(0.4, 0.63)
0.04
(0.04, 0.04)
0.03
(0.03, 0.03)
0.17
(0.15, 0.19)
0.98
(0.88, 1.08)
0
(0, 0)
Song–plastic
Lower Rate
Song–plastic
Higher Rate
0.05
(0.05, 0.05)
Original threshold range: 1.1429 – 130.435 (36)
p–value
0.0
0.2
0.4
0.6
0.8
1
2
5
10
20
50
100
High/Low Threshold: Rate

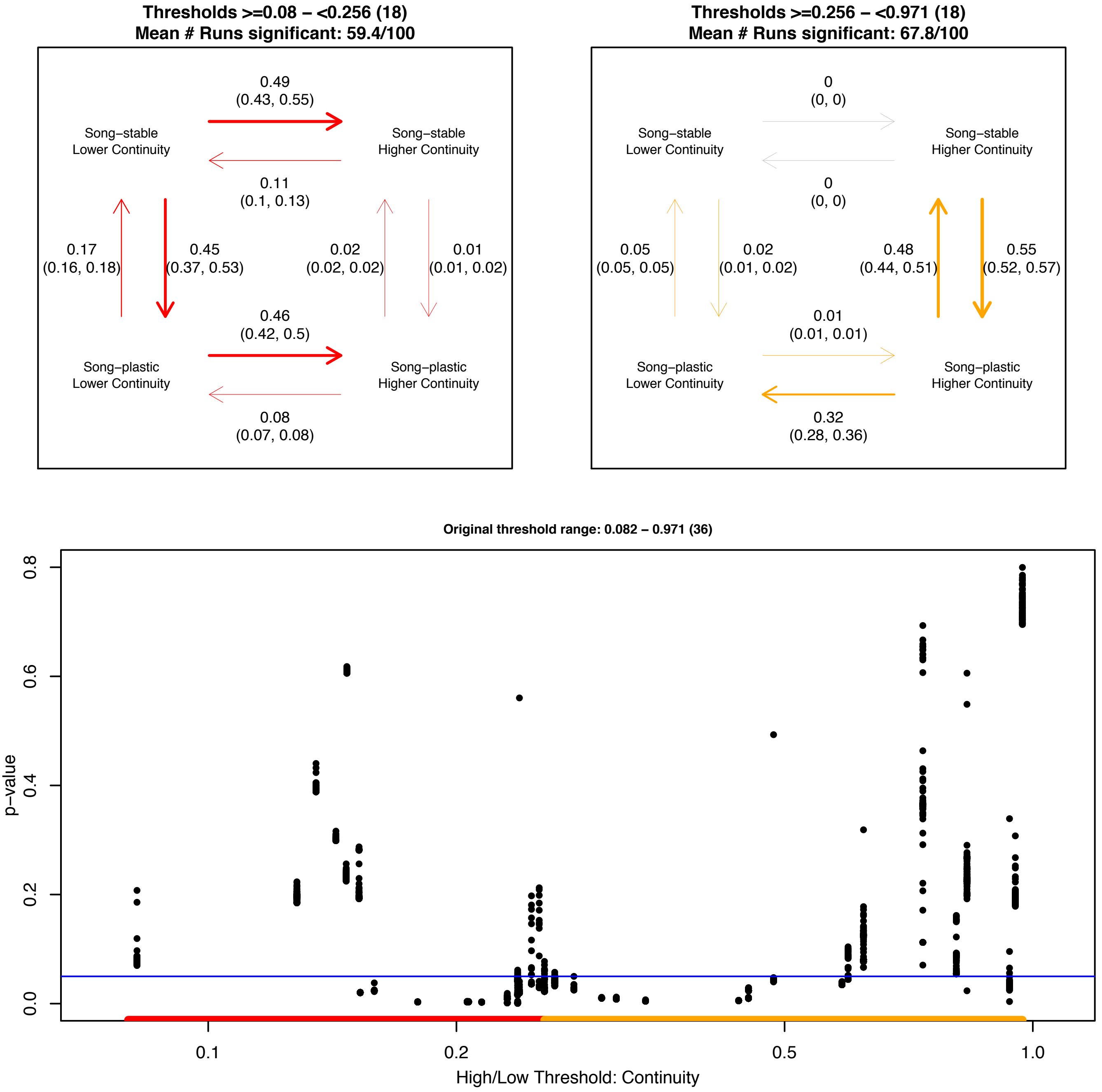

Thresholds >=0.08 – <0.256 (18)
Mean # Runs significant: 59.4/100
0.49
(0.43, 0.55)
Song–stable
Lower Continuity
Song–stable
Higher Continuity
0.11
(0.1, 0.13)
0.17
(0.16, 0.18)
0.45
(0.37, 0.53)
0.02
(0.02, 0.02)
0.01
(0.01, 0.02)
0.46
(0.42, 0.5)
Song–plastic
Lower Continuity
Song–plastic
Higher Continuity
0.08
(0.07, 0.08)
Thresholds >=0.256 – <0.971 (18)
Mean # Runs significant: 67.8/100
0
(0, 0)
Song–stable
Lower Continuity
Song–stable
Higher Continuity
0
(0, 0)
0.05
(0.05, 0.05)
0.02
(0.01, 0.02)
0.48
(0.44, 0.51)
0.55
(0.52, 0.57)
0.01
(0.01, 0.01)
Song–plastic
Lower Continuity
Song–plastic
Higher Continuity
0.32
(0.28, 0.36)
Original threshold range: 0.082 – 0.971 (36)
p–value
0.0
0.2
0.4
0.6
0.8
0.1
0.2
0.5
1.0
High/Low Threshold: Continuity

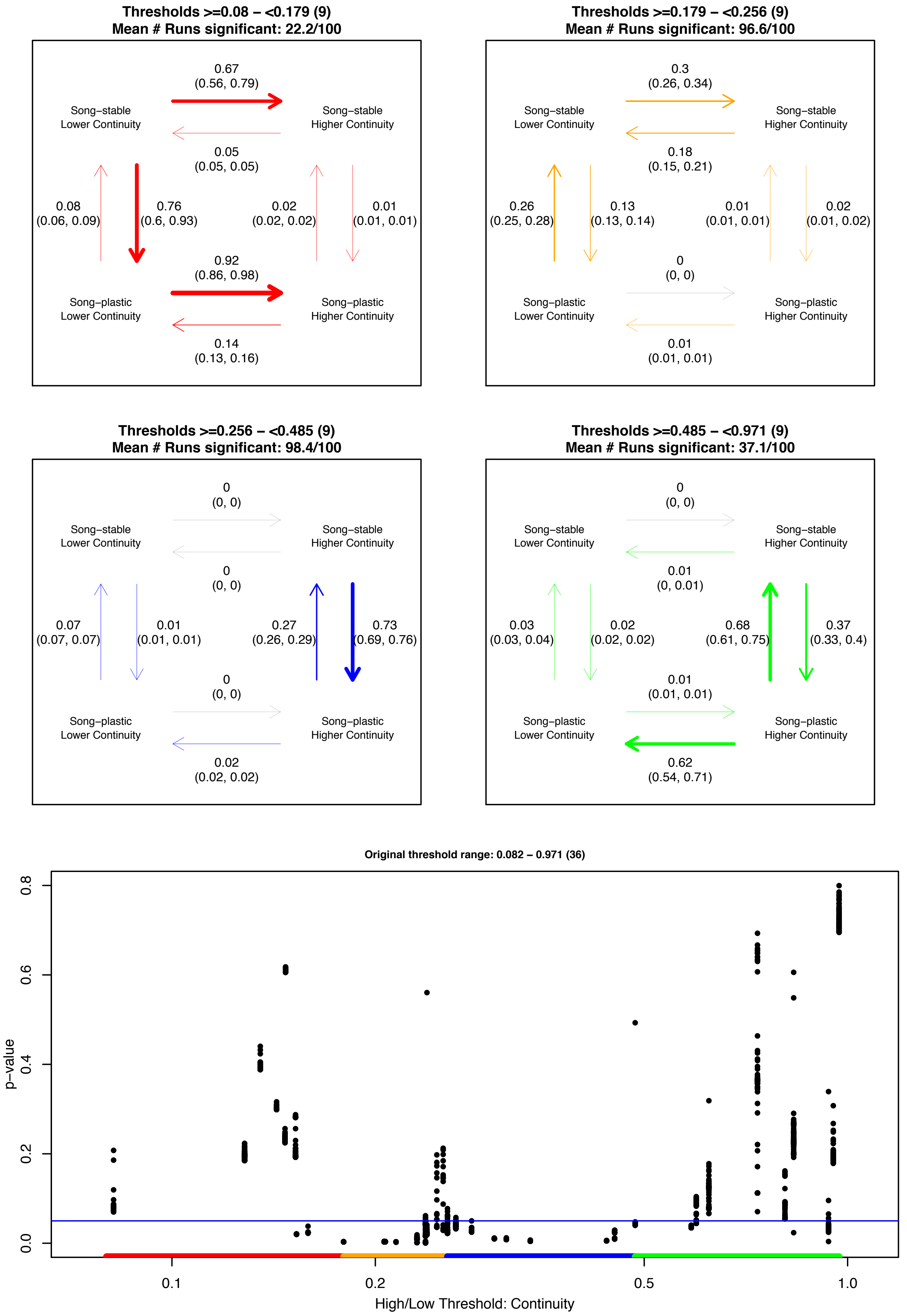
Thresholds >=0.08 – <0.179 (9)
Mean # Runs significant: 22.2/100
0.67
(0.56, 0.79)
Song−stable
Lower Continuity
Song−stable
Higher Continuity
0.05
(0.05, 0.05)
0.08
(0.06, 0.09)
0.76
(0.6, 0.93)
0.02
(0.02, 0.02)
0.01
(0.01, 0.01)
0.92
(0.86, 0.98)
Song−plastic
Lower Continuity
Song−plastic
Higher Continuity
0.14
(0.13, 0.16)
Thresholds >=0.179 – <0.256 (9)
Mean # Runs significant: 96.6/100
0.3
(0.26, 0.34)
Song−stable
Lower Continuity
Song−stable
Higher Continuity
0.18
(0.15, 0.21)
0.26
(0.25, 0.28)
0.13
(0.13, 0.14)
0.01
(0.01, 0.01)
0.02
(0.01, 0.02)
0
(0, 0)
Song−plastic
Lower Continuity
Song−plastic
Higher Continuity
0.01
(0.01, 0.01)
Thresholds >=0.256 – <0.485 (9)
Mean # Runs significant: 98.4/100
0
(0, 0)
Song−stable
Lower Continuity
Song−stable
Higher Continuity
0
(0, 0)
0.07
(0.07, 0.07)
0.01
(0.01, 0.01)
0.27
(0.26, 0.29)
0.73
(0.69, 0.76)
0
(0, 0)
Song−plastic
Lower Continuity
Song−plastic
Higher Continuity
0.02
(0.02, 0.02)
Thresholds >=0.485 – <0.971 (9)
Mean # Runs significant: 37.1/100
0
(0, 0)
Song−stable
Lower Continuity
Song−stable
Higher Continuity
0.01
(0, 0.01)
0.03
(0.03, 0.04)
0.02
(0.02, 0.02)
0.68
(0.61, 0.75)
0.37
(0.33, 0.4)
0.01
(0.01, 0.01)
Song−plastic
Lower Continuity
Song−plastic
Higher Continuity
0.62
(0.54, 0.71)
Original threshold range: 0.082 – 0.971 (36)
p−value
0.0
0.2
0.4
0.6
0.8
0.1
0.2
0.5
1.0
High/Low Threshold: Continuity

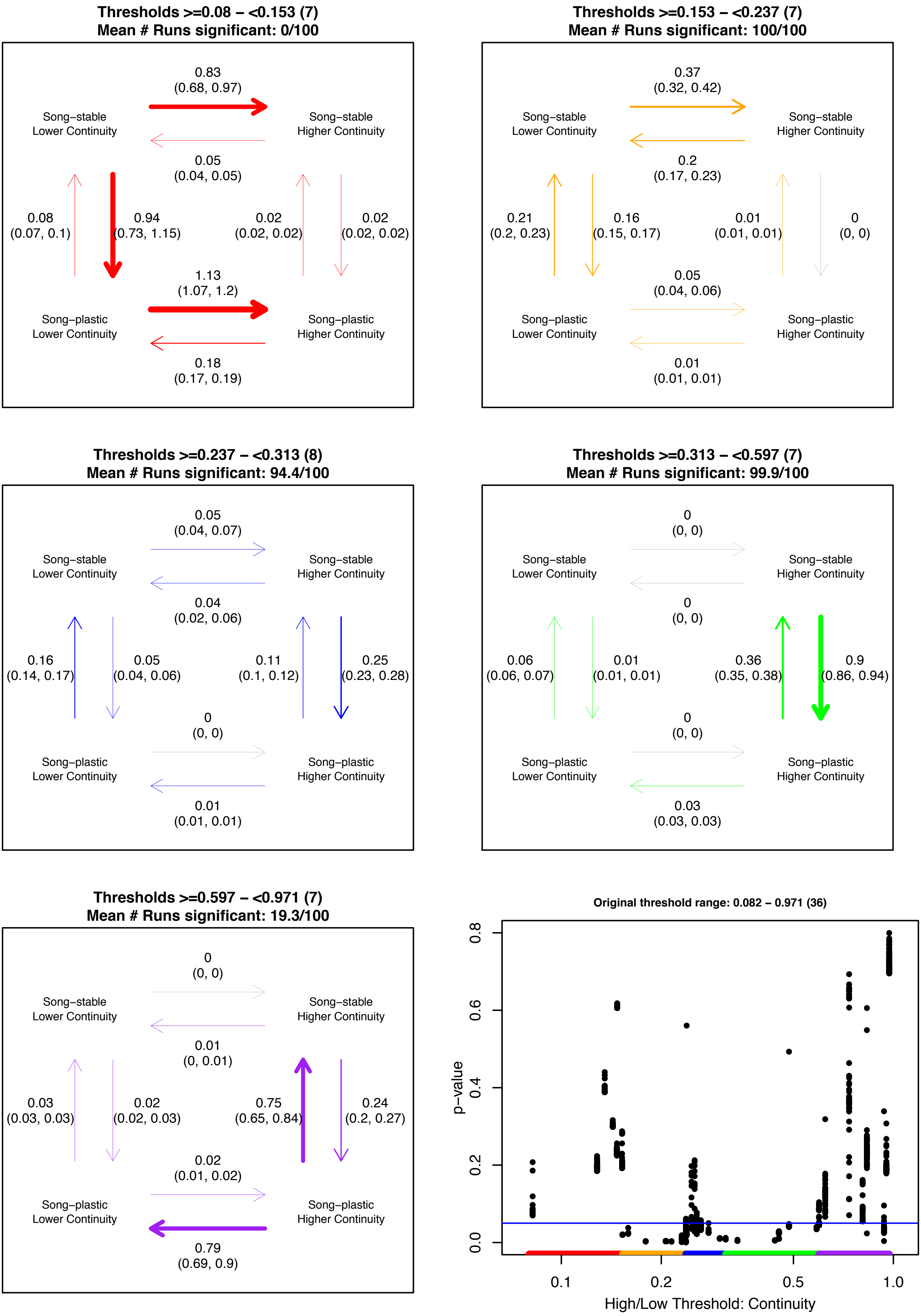

Thresholds >=0.08 – <0.153 (7)
Mean # Runs significant: 0/100
Song–stable Lower Continuity
Song–stable Higher Continuity
Song–plastic Lower Continuity
Song–plastic Higher Continuity
0.83 (0.68, 0.97)
0.05 (0.04, 0.05)
0.08 (0.07, 0.1)
0.94 (0.73, 1.15)
0.02 (0.02, 0.02)
0.02 (0.02, 0.02)
1.13 (1.07, 1.2)
0.18 (0.17, 0.19)
Thresholds >=0.153 – <0.237 (7)
Mean # Runs significant: 100/100
0.37 (0.32, 0.42)
0.2 (0.17, 0.23)
0.21 (0.2, 0.23)
0.16 (0.15, 0.17)
0.01 (0.01, 0.01)
0 (0, 0)
0.05 (0.04, 0.06)
0.01 (0.01, 0.01)
Thresholds >=0.237 – <0.313 (8)
Mean # Runs significant: 94.4/100
0.05 (0.04, 0.07)
0.04 (0.02, 0.06)
0.16 (0.14, 0.17)
0.05 (0.04, 0.06)
0.11 (0.1, 0.12)
0.25 (0.23, 0.28)
0 (0, 0)
0.01 (0.01, 0.01)
Thresholds >=0.313 – <0.597 (7)
Mean # Runs significant: 99.9/100
0 (0, 0)
0 (0, 0)
0.06 (0.06, 0.07)
0.01 (0.01, 0.01)
0.36 (0.35, 0.38)
0.9 (0.86, 0.94)
0 (0, 0)
0.03 (0.03, 0.03)
Thresholds >=0.597 – <0.971 (7)
Mean # Runs significant: 19.3/100
0 (0, 0)
0.01 (0, 0.01)
0.03 (0.03, 0.03)
0.02 (0.02, 0.03)
0.75 (0.65, 0.84)
0.24 (0.2, 0.27)
0.02 (0.01, 0.02)
0.79 (0.69, 0.9)
Original threshold range: 0.082 – 0.971 (36)
p–value
0.0
0.2
0.4
0.6
0.8
0.1
0.2
0.5
1.0
High/Low Threshold: Continuity
