# Supplementary material for: Correlated evolution between repertoire size and song plasticity predicts that sexual selection on song promotes open-ended learning
Source: eLife. 2019 Sep 3;8:e44454. doi: 10.7554/eLife.44454 (PMC6721395; doi:10.7554/eLife.44454)
Supplement: Figure 6—source data 2. — Labeling the same as in Figure 6 and Figure 6—figure supplement 1. [file elife-44454-fig6-data2.pdf]

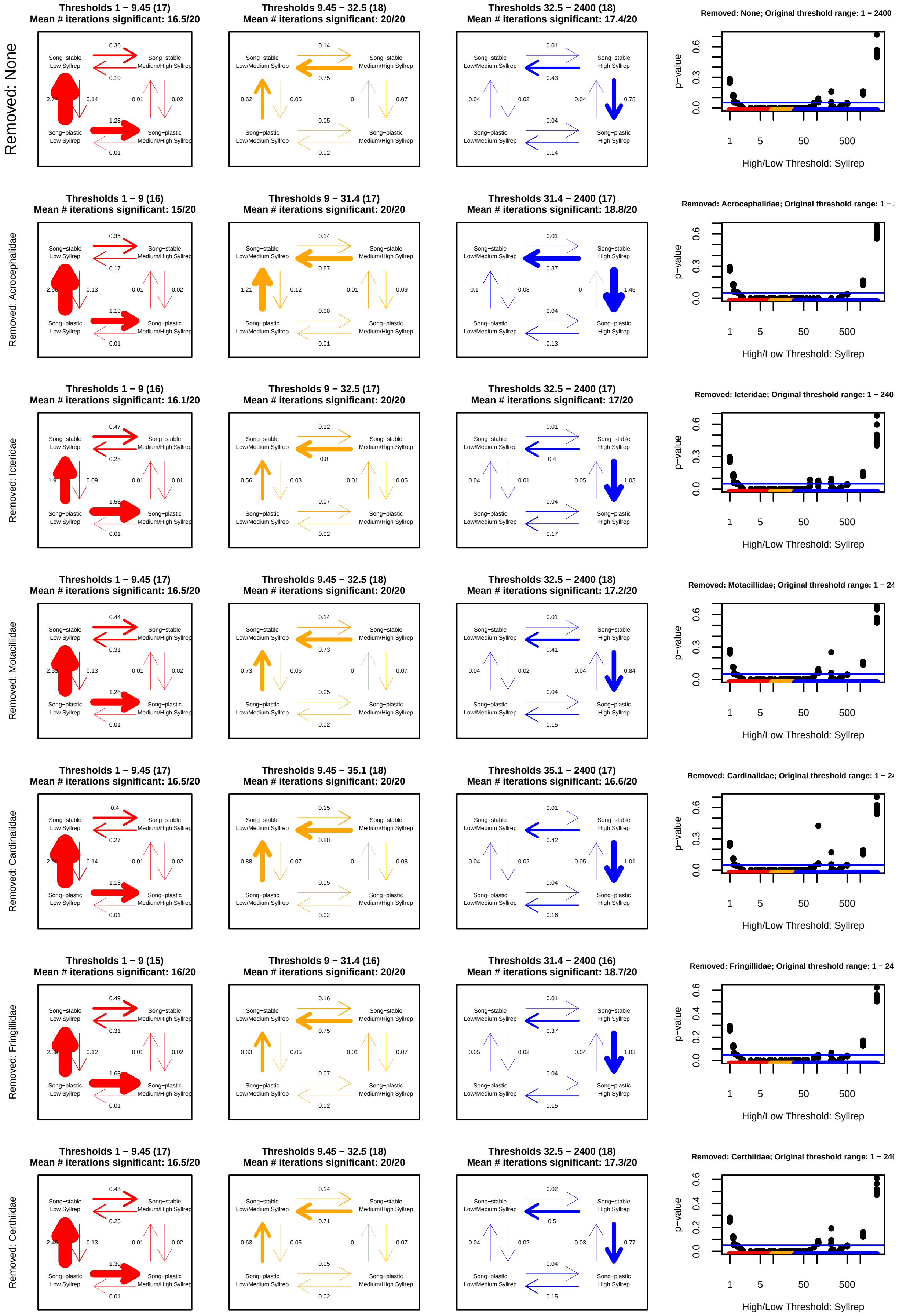

Removed: None
Thresholds 1 − 9.45 (17)
Mean # iterations significant: 16.5/20
Song−stable Low Syllrep
Song−stable Medium/High Syllrep
Song−plastic Low Syllrep
Song−plastic Medium/High Syllrep
0.36
0.19
2.77
0.14
0.01
0.02
1.28
0.01
Thresholds 9.45 − 32.5 (18)
Mean # iterations significant: 20/20
Song−stable Low/Medium Syllrep
Song−stable Medium/High Syllrep
Song−plastic Low/Medium Syllrep
Song−plastic Medium/High Syllrep
0.14
0.75
0.62
0.05
0
0.07
0.05
0.02
Thresholds 32.5 − 2400 (18)
Mean # iterations significant: 17.4/20
Song−stable Low/Medium Syllrep
Song−stable High Syllrep
Song−plastic Low/Medium Syllrep
Song−plastic High Syllrep
0.01
0.43
0.04
0.02
0.04
0.78
0.04
0.14
Removed: None; Original threshold range: 1 − 2400
p−value
0.0
0.3
0.6
1
5
50
500
High/Low Threshold: Syllrep
Removed: Acrocephalidae
Thresholds 1 − 9 (16)
Mean # iterations significant: 15/20
Song−stable Low Syllrep
Song−stable Medium/High Syllrep
Song−plastic Low Syllrep
Song−plastic Medium/High Syllrep
0.35
0.17
2.66
0.13
0.01
0.02
1.19
0.01
Thresholds 9 − 31.4 (17)
Mean # iterations significant: 20/20
Song−stable Low/Medium Syllrep
Song−stable Medium/High Syllrep
Song−plastic Low/Medium Syllrep
Song−plastic Medium/High Syllrep
0.14
0.87
1.21
0.12
0.01
0.09
0.08
0.01
Thresholds 31.4 − 2400 (17)
Mean # iterations significant: 18.8/20
Song−stable Low/Medium Syllrep
Song−stable High Syllrep
Song−plastic Low/Medium Syllrep
Song−plastic High Syllrep
0.01
0.87
0.1
0.03
0
1.45
0.04
0.13
Removed: Acrocephalidae; Original threshold range: 1 −
p−value
0.0
0.3
0.6
1
5
50
500
High/Low Threshold: Syllrep
Removed: Icteridae
Thresholds 1 − 9 (16)
Mean # iterations significant: 16.1/20
Song−stable Low Syllrep
Song−stable Medium/High Syllrep
Song−plastic Low Syllrep
Song−plastic Medium/High Syllrep
0.47
0.28
1.9
0.09
0.01
0.01
1.53
0.01
Thresholds 9 − 32.5 (17)
Mean # iterations significant: 20/20
Song−stable Low/Medium Syllrep
Song−stable Medium/High Syllrep
Song−plastic Low/Medium Syllrep
Song−plastic Medium/High Syllrep
0.12
0.8
0.56
0.03
0.01
0.05
0.07
0.02
Thresholds 32.5 − 2400 (17)
Mean # iterations significant: 17/20
Song−stable Low/Medium Syllrep
Song−stable High Syllrep
Song−plastic Low/Medium Syllrep
Song−plastic High Syllrep
0.01
0.4
0.04
0.01
0.05
1.03
0.04
0.17
Removed: Icteridae; Original threshold range: 1 − 240
p−value
0.0
0.3
0.6
1
5
50
500
High/Low Threshold: Syllrep
Removed: Motacillidae
Thresholds 1 − 9.45 (17)
Mean # iterations significant: 16.5/20
Song−stable Low Syllrep
Song−stable Medium/High Syllrep
Song−plastic Low Syllrep
Song−plastic Medium/High Syllrep
0.44
0.31
2.53
0.13
0.01
0.02
1.28
0.01
Thresholds 9.45 − 32.5 (18)
Mean # iterations significant: 20/20
Song−stable Low/Medium Syllrep
Song−stable Medium/High Syllrep
Song−plastic Low/Medium Syllrep
Song−plastic Medium/High Syllrep
0.14
0.73
0.73
0.06
0
0.07
0.05
0.02
Thresholds 32.5 − 2400 (18)
Mean # iterations significant: 17.2/20
Song−stable Low/Medium Syllrep
Song−stable High Syllrep
Song−plastic Low/Medium Syllrep
Song−plastic High Syllrep
0.01
0.41
0.04
0.02
0.04
0.84
0.04
0.15
Removed: Motacillidae; Original threshold range: 1 − 24
p−value
0.0
0.3
0.6
1
5
50
500
High/Low Threshold: Syllrep
Removed: Cardinalidae
Thresholds 1 − 9.45 (17)
Mean # iterations significant: 16.5/20
Song−stable Low Syllrep
Song−stable Medium/High Syllrep
Song−plastic Low Syllrep
Song−plastic Medium/High Syllrep
0.4
0.27
2.59
0.14
0.01
0.02
1.13
0.01
Thresholds 9.45 − 35.1 (18)
Mean # iterations significant: 20/20
Song−stable Low/Medium Syllrep
Song−stable Medium/High Syllrep
Song−plastic Low/Medium Syllrep
Song−plastic Medium/High Syllrep
0.15
0.88
0.88
0.07
0
0.08
0.05
0.02
Thresholds 35.1 − 2400 (17)
Mean # iterations significant: 16.6/20
Song−stable Low/Medium Syllrep
Song−stable High Syllrep
Song−plastic Low/Medium Syllrep
Song−plastic High Syllrep
0.01
0.42
0.04
0.02
0.05
1.01
0.04
0.16
Removed: Cardinalidae; Original threshold range: 1 − 24
p−value
0.0
0.3
0.6
1
5
50
500
High/Low Threshold: Syllrep
Removed: Fringillidae
Thresholds 1 − 9 (15)
Mean # iterations significant: 16/20
Song−stable Low Syllrep
Song−stable Medium/High Syllrep
Song−plastic Low Syllrep
Song−plastic Medium/High Syllrep
0.49
0.31
2.39
0.12
0.01
0.02
1.63
0.01
Thresholds 9 − 31.4 (16)
Mean # iterations significant: 20/20
Song−stable Low/Medium Syllrep
Song−stable Medium/High Syllrep
Song−plastic Low/Medium Syllrep
Song−plastic Medium/High Syllrep
0.16
0.75
0.63
0.05
0.01
0.07
0.07
0.02
Thresholds 31.4 − 2400 (16)
Mean # iterations significant: 18.7/20
Song−stable Low/Medium Syllrep
Song−stable High Syllrep
Song−plastic Low/Medium Syllrep
Song−plastic High Syllrep
0.01
0.37
0.05
0.02
0.04
1.03
0.04
0.15
Removed: Fringillidae; Original threshold range: 1 − 24
p−value
0.0
0.2
0.4
0.6
1
5
50
500
High/Low Threshold: Syllrep
Removed: Certhiidae
Thresholds 1 − 9.45 (17)
Mean # iterations significant: 16.5/20
Song−stable Low Syllrep
Song−stable Medium/High Syllrep
Song−plastic Low Syllrep
Song−plastic Medium/High Syllrep
0.43
0.25
2.45
0.13
0.01
0.02
1.39
0.01
Thresholds 9.45 − 32.5 (18)
Mean # iterations significant: 20/20
Song−stable Low/Medium Syllrep
Song−stable Medium/High Syllrep
Song−plastic Low/Medium Syllrep
Song−plastic Medium/High Syllrep
0.14
0.71
0.63
0.05
0
0.07
0.05
0.02
Thresholds 32.5 − 2400 (18)
Mean # iterations significant: 17.3/20
Song−stable Low/Medium Syllrep
Song−stable High Syllrep
Song−plastic Low/Medium Syllrep
Song−plastic High Syllrep
0.02
0.5
0.04
0.02
0.03
0.77
0.04
0.15
Removed: Certhiidae; Original threshold range: 1 − 240
p−value
0.0
0.2
0.4
0.6
1
5
50
500
High/Low Threshold: Syllrep

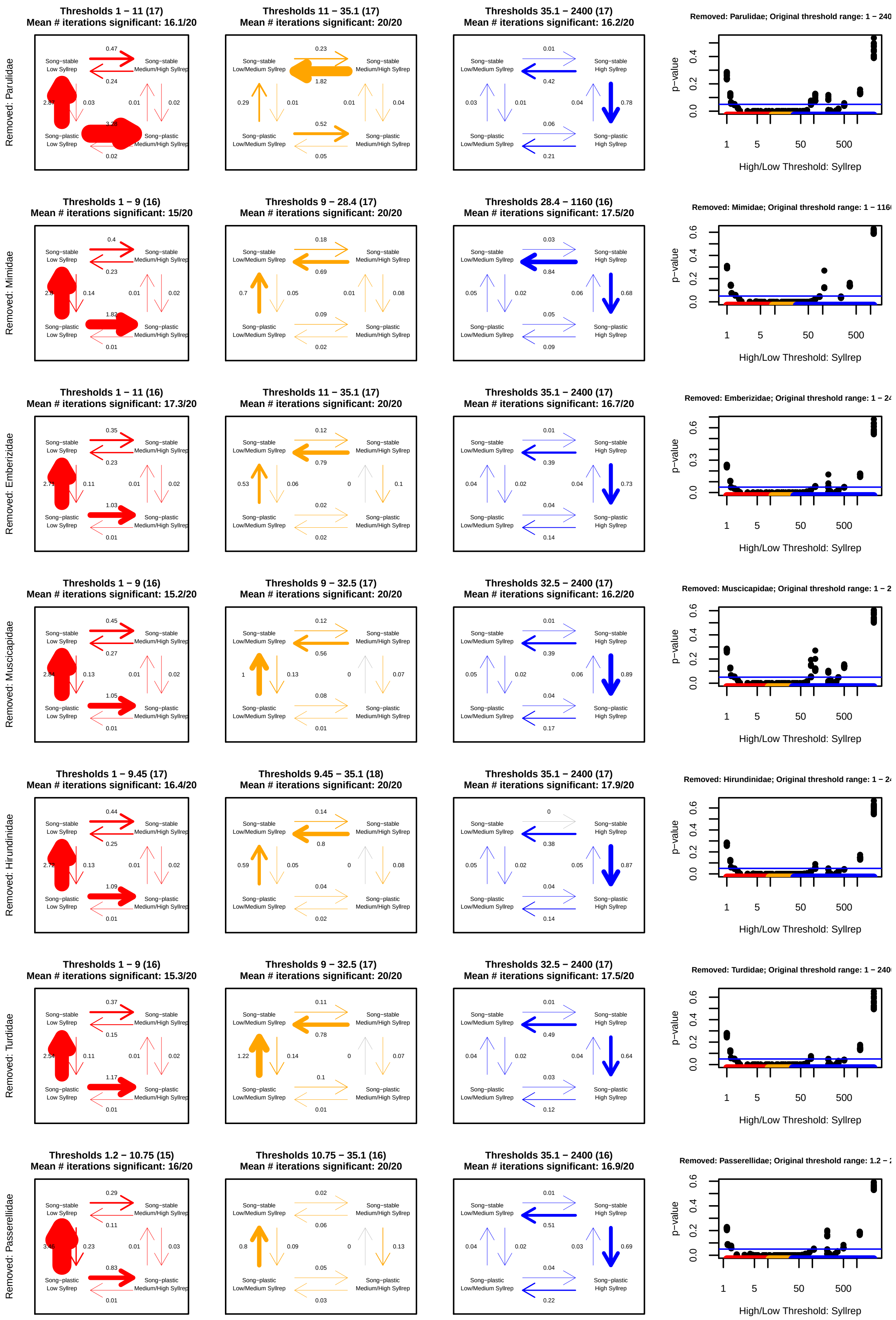

Removed: Parulidae
Thresholds 1 − 11 (17)
Mean # iterations significant: 16.1/20
Song−stable Low Syllrep
Song−stable Medium/High Syllrep
Song−plastic Low Syllrep
Song−plastic Medium/High Syllrep
0.47 0.24 2.87 0.03 0.01 0.02 3.28 0.02
Thresholds 11 − 35.1 (17)
Mean # iterations significant: 20/20
Song−stable Low/Medium Syllrep
Song−stable Medium/High Syllrep
Song−plastic Low/Medium Syllrep
Song−plastic Medium/High Syllrep
0.23 1.82 0.29 0.01 0.01 0.04 0.52 0.05
Thresholds 35.1 − 2400 (17)
Mean # iterations significant: 16.2/20
Song−stable Low/Medium Syllrep
Song−stable High Syllrep
Song−plastic Low/Medium Syllrep
Song−plastic High Syllrep
0.01 0.42 0.03 0.01 0.04 0.78 0.06 0.21
Removed: Parulidae; Original threshold range: 1 − 240
p−value
0.0 0.2 0.4
1 5 50 500
High/Low Threshold: Syllrep
Removed: Mimidae
Thresholds 1 − 9 (16)
Mean # iterations significant: 15/20
0.4 0.23 2.8 0.14 0.01 0.02 1.82 0.01
Thresholds 9 − 28.4 (17)
Mean # iterations significant: 20/20
0.18 0.69 0.7 0.05 0.01 0.08 0.09 0.02
Thresholds 28.4 − 1160 (16)
Mean # iterations significant: 17.5/20
0.03 0.84 0.05 0.02 0.06 0.68 0.05 0.09
Removed: Mimidae; Original threshold range: 1 − 116
p−value
0.0 0.2 0.4 0.6
1 5 50 500
High/Low Threshold: Syllrep
Removed: Emberizidae
Thresholds 1 − 11 (16)
Mean # iterations significant: 17.3/20
0.35 0.23 2.71 0.11 0.01 0.02 1.03 0.01
Thresholds 11 − 35.1 (17)
Mean # iterations significant: 20/20
0.12 0.79 0.53 0.06 0 0.1 0.02 0.02
Thresholds 35.1 − 2400 (17)
Mean # iterations significant: 16.7/20
0.01 0.39 0.04 0.02 0.04 0.73 0.04 0.14
Removed: Emberizidae; Original threshold range: 1 − 24
p−value
0.0 0.3 0.6
1 5 50 500
High/Low Threshold: Syllrep
Removed: Muscicapidae
Thresholds 1 − 9 (16)
Mean # iterations significant: 15.2/20
0.45 0.27 2.84 0.13 0.01 0.02 1.05 0.01
Thresholds 9 − 32.5 (17)
Mean # iterations significant: 20/20
0.12 0.56 1 0.13 0 0.07 0.08 0.01
Thresholds 32.5 − 2400 (17)
Mean # iterations significant: 16.2/20
0.01 0.39 0.05 0.02 0.06 0.89 0.04 0.17
Removed: Muscicapidae; Original threshold range: 1 − 2
p−value
0.0 0.2 0.4 0.6
1 5 50 500
High/Low Threshold: Syllrep
Removed: Hirundinidae
Thresholds 1 − 9.45 (17)
Mean # iterations significant: 16.4/20
0.44 0.25 2.72 0.13 0.01 0.02 1.09 0.01
Thresholds 9.45 − 35.1 (18)
Mean # iterations significant: 20/20
0.14 0.8 0.59 0.05 0 0.08 0.04 0.02
Thresholds 35.1 − 2400 (17)
Mean # iterations significant: 17.9/20
0 0.38 0.05 0.02 0.05 0.87 0.04 0.14
Removed: Hirundinidae; Original threshold range: 1 − 24
p−value
0.0 0.2 0.4 0.6
1 5 50 500
High/Low Threshold: Syllrep
Removed: Turdidae
Thresholds 1 − 9 (16)
Mean # iterations significant: 15.3/20
0.37 0.15 2.54 0.11 0.01 0.02 1.17 0.01
Thresholds 9 − 32.5 (17)
Mean # iterations significant: 20/20
0.11 0.78 1.22 0.14 0 0.07 0.1 0.01
Thresholds 32.5 − 2400 (17)
Mean # iterations significant: 17.5/20
0.01 0.49 0.04 0.02 0.04 0.64 0.03 0.12
Removed: Turdidae; Original threshold range: 1 − 240
p−value
0.0 0.2 0.4 0.6
1 5 50 500
High/Low Threshold: Syllrep
Removed: Passerellidae
Thresholds 1.2 − 10.75 (15)
Mean # iterations significant: 16/20
0.29 0.11 3.45 0.23 0.01 0.03 0.83 0.01
Thresholds 10.75 − 35.1 (16)
Mean # iterations significant: 20/20
0.02 0.06 0.8 0.09 0 0.13 0.05 0.03
Thresholds 35.1 − 2400 (16)
Mean # iterations significant: 16.9/20
0.01 0.51 0.04 0.02 0.03 0.69 0.04 0.22
Removed: Passerellidae; Original threshold range: 1.2 −
p−value
0.0 0.2 0.4 0.6
1 5 50 500
High/Low Threshold: Syllrep

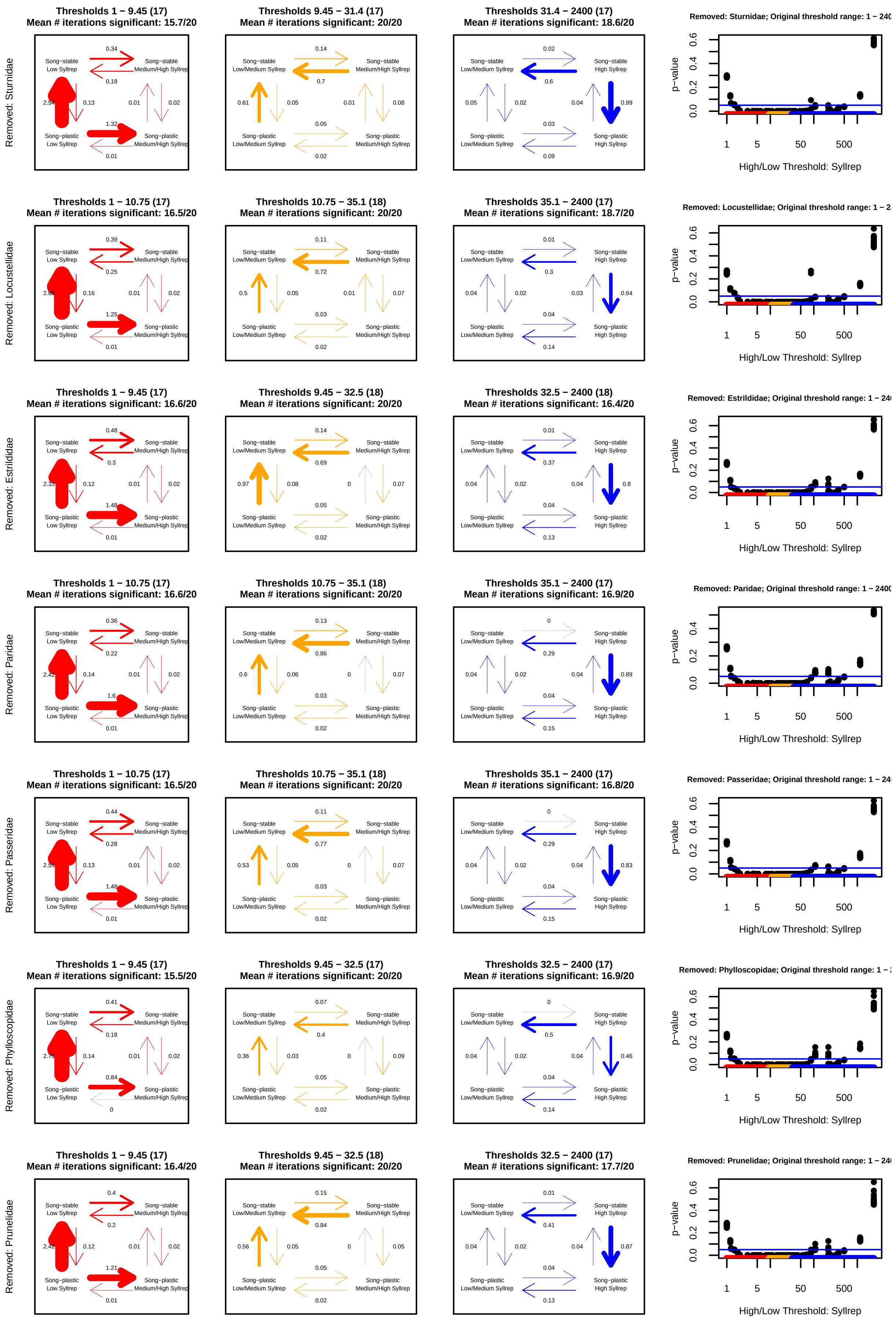
Removed: Sturnidae
Thresholds 1 – 9.45 (17)
Mean # iterations significant: 15.7/20
Thresholds 9.45 – 31.4 (17)
Mean # iterations significant: 20/20
Thresholds 31.4 – 2400 (17)
Mean # iterations significant: 18.6/20
Removed: Sturnidae; Original threshold range: 1 – 240
Removed: Locustellidae
Thresholds 1 – 10.75 (17)
Mean # iterations significant: 16.5/20
Thresholds 10.75 – 35.1 (18)
Mean # iterations significant: 20/20
Thresholds 35.1 – 2400 (17)
Mean # iterations significant: 18.7/20
Removed: Locustellidae; Original threshold range: 1 – 2
Removed: Estrildidae
Thresholds 1 – 9.45 (17)
Mean # iterations significant: 16.6/20
Thresholds 9.45 – 32.5 (18)
Mean # iterations significant: 20/20
Thresholds 32.5 – 2400 (18)
Mean # iterations significant: 16.4/20
Removed: Estrildidae; Original threshold range: 1 – 24
Removed: Paridae
Thresholds 1 – 10.75 (17)
Mean # iterations significant: 16.6/20
Thresholds 10.75 – 35.1 (18)
Mean # iterations significant: 20/20
Thresholds 35.1 – 2400 (17)
Mean # iterations significant: 16.9/20
Removed: Paridae; Original threshold range: 1 – 2400
Removed: Passeridae
Thresholds 1 – 10.75 (17)
Mean # iterations significant: 16.5/20
Thresholds 10.75 – 35.1 (18)
Mean # iterations significant: 20/20
Thresholds 35.1 – 2400 (17)
Mean # iterations significant: 16.8/20
Removed: Passeridae; Original threshold range: 1 – 24
Removed: Phylloscopidae
Thresholds 1 – 9.45 (17)
Mean # iterations significant: 15.5/20
Thresholds 9.45 – 32.5 (17)
Mean # iterations significant: 20/20
Thresholds 32.5 – 2400 (17)
Mean # iterations significant: 16.9/20
Removed: Phylloscopidae; Original threshold range: 1 –
Removed: Prunelidae
Thresholds 1 – 9.45 (17)
Mean # iterations significant: 16.4/20
Thresholds 9.45 – 32.5 (18)
Mean # iterations significant: 20/20
Thresholds 32.5 – 2400 (17)
Mean # iterations significant: 17.7/20
Removed: Prunelidae; Original threshold range: 1 – 24
Song−stable
Song−plastic
Low Syllrep
Low/Medium Syllrep
Medium/High Syllrep
High Syllrep
p−value
High/Low Threshold: Syllrep

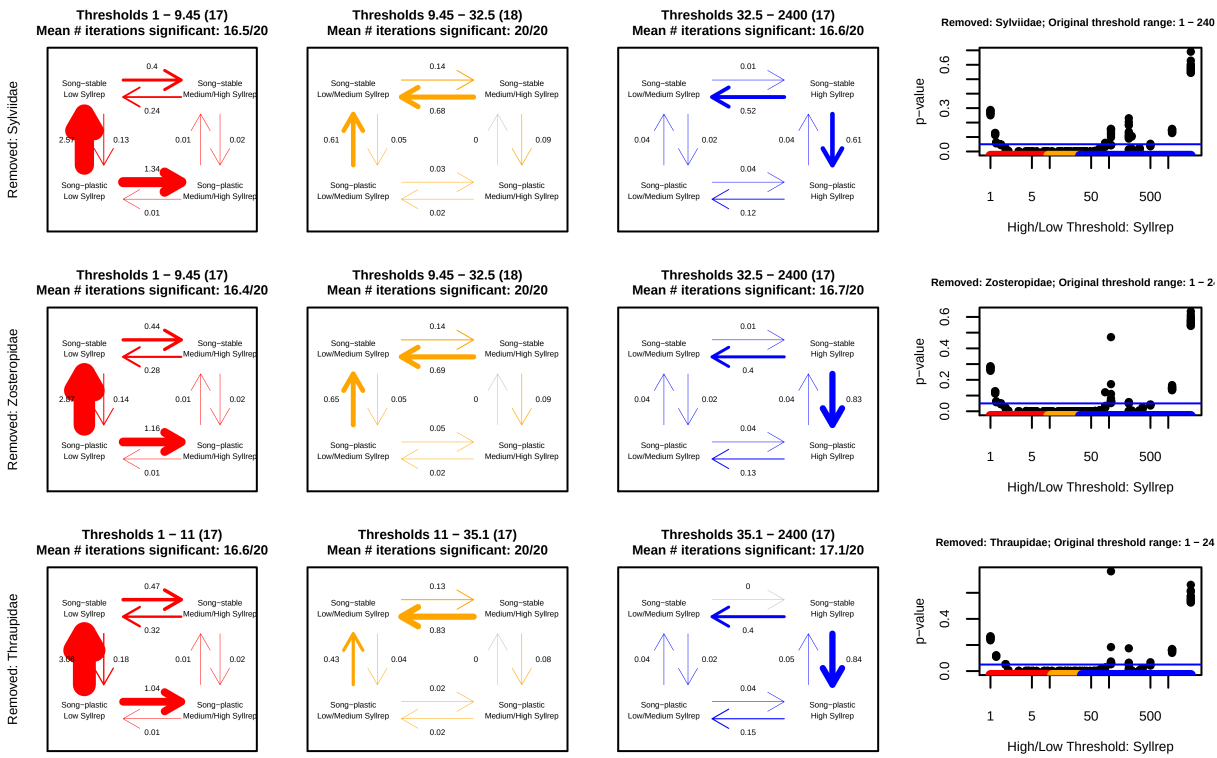

Thresholds 1 − 9.45 (17)
Mean # iterations significant: 16.5/20
Thresholds 9.45 − 32.5 (18)
Mean # iterations significant: 20/20
Thresholds 32.5 − 2400 (17)
Mean # iterations significant: 16.6/20
Removed: Sylviidae; Original threshold range: 1 − 240
Removed: Sylviidae
Song−stable Low Syllrep
Song−stable Medium/High Syllrep
Song−plastic Low Syllrep
Song−plastic Medium/High Syllrep
0.4
0.24
2.57
0.13
0.01
0.02
1.34
0.01
Song−stable Low/Medium Syllrep
Song−stable Medium/High Syllrep
Song−plastic Low/Medium Syllrep
Song−plastic Medium/High Syllrep
0.14
0.68
0.61
0.05
0
0.09
0.03
0.02
Song−stable Low/Medium Syllrep
Song−stable High Syllrep
Song−plastic Low/Medium Syllrep
Song−plastic High Syllrep
0.01
0.52
0.04
0.02
0.04
0.61
0.04
0.12
p−value
0.0
0.3
0.6
1
5
50
500
High/Low Threshold: Syllrep
Thresholds 1 − 9.45 (17)
Mean # iterations significant: 16.4/20
Thresholds 9.45 − 32.5 (18)
Mean # iterations significant: 20/20
Thresholds 32.5 − 2400 (17)
Mean # iterations significant: 16.7/20
Removed: Zosteropidae; Original threshold range: 1 − 2
Removed: Zosteropidae
Song−stable Low Syllrep
Song−stable Medium/High Syllrep
Song−plastic Low Syllrep
Song−plastic Medium/High Syllrep
0.44
0.28
2.87
0.14
0.01
0.02
1.16
0.01
Song−stable Low/Medium Syllrep
Song−stable Medium/High Syllrep
Song−plastic Low/Medium Syllrep
Song−plastic Medium/High Syllrep
0.14
0.69
0.65
0.05
0
0.09
0.05
0.02
Song−stable Low/Medium Syllrep
Song−stable High Syllrep
Song−plastic Low/Medium Syllrep
Song−plastic High Syllrep
0.01
0.4
0.04
0.02
0.04
0.83
0.04
0.13
p−value
0.0
0.2
0.4
0.6
1
5
50
500
High/Low Threshold: Syllrep
Thresholds 1 − 11 (17)
Mean # iterations significant: 16.6/20
Thresholds 11 − 35.1 (17)
Mean # iterations significant: 20/20
Thresholds 35.1 − 2400 (17)
Mean # iterations significant: 17.1/20
Removed: Thraupidae; Original threshold range: 1 − 24
Removed: Thraupidae
Song−stable Low Syllrep
Song−stable Medium/High Syllrep
Song−plastic Low Syllrep
Song−plastic Medium/High Syllrep
0.47
0.32
3.06
0.18
0.01
0.02
1.04
0.01
Song−stable Low/Medium Syllrep
Song−stable Medium/High Syllrep
Song−plastic Low/Medium Syllrep
Song−plastic Medium/High Syllrep
0.13
0.83
0.43
0.04
0
0.08
0.02
0.02
Song−stable Low/Medium Syllrep
Song−stable High Syllrep
Song−plastic Low/Medium Syllrep
Song−plastic High Syllrep
0
0.4
0.04
0.02
0.05
0.84
0.04
0.15
p−value
0.0
0.4
1
5
50
500
High/Low Threshold: Syllrep
