# Supplementary material for: Correlated evolution between repertoire size and song plasticity predicts that sexual selection on song promotes open-ended learning
Source: eLife. 2019 Sep 3;8:e44454. doi: 10.7554/eLife.44454 (PMC6721395; doi:10.7554/eLife.44454)
Supplement: Figure 6—source data 3. — Labeling the same as in Figure 6 and Figure 6—figure supplement 1. [file elife-44454-fig6-data3.pdf]

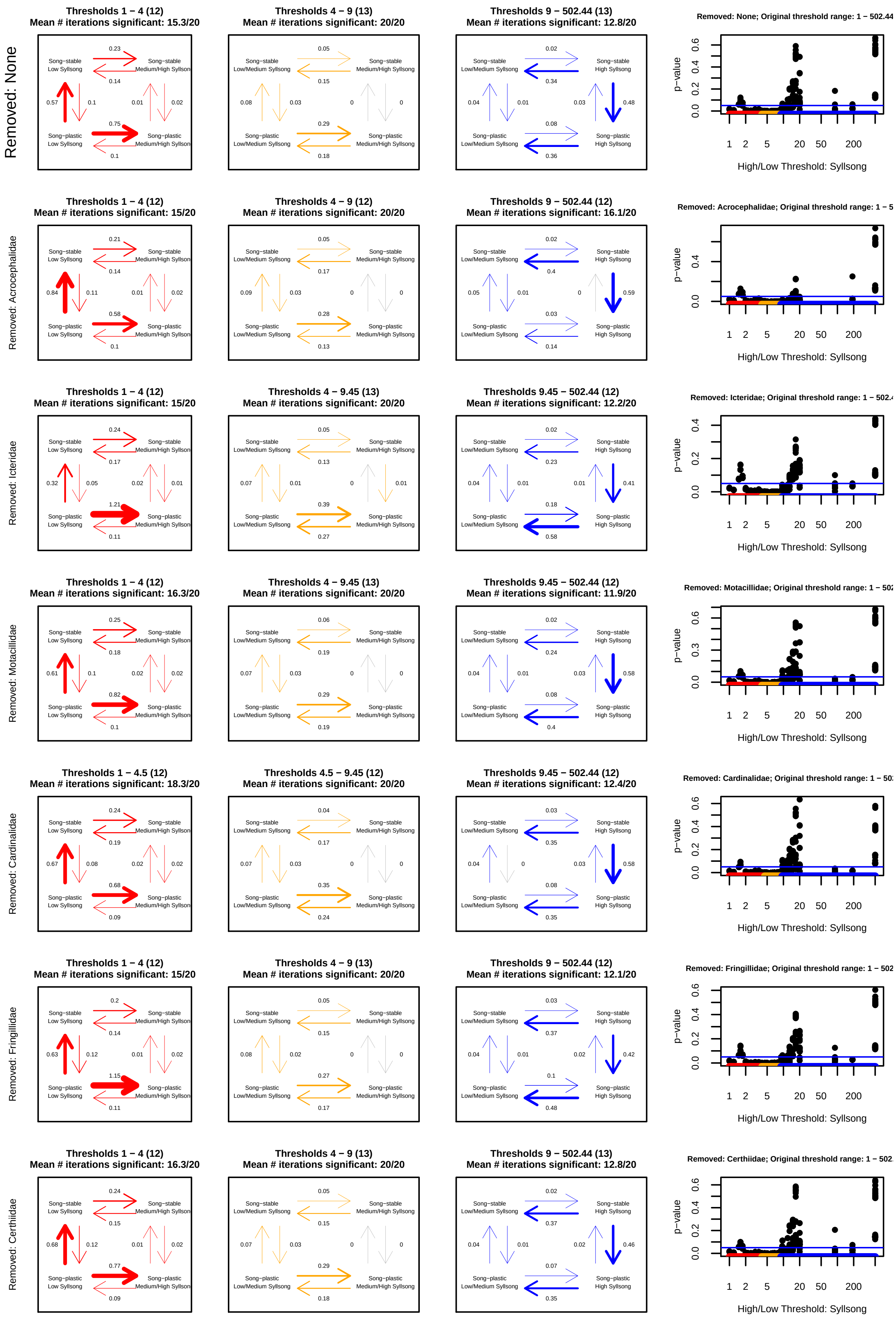

Removed: None
Thresholds 1 – 4 (12)
Mean # iterations significant: 15.3/20
Thresholds 4 – 9 (13)
Mean # iterations significant: 20/20
Thresholds 9 – 502.44 (13)
Mean # iterations significant: 12.8/20
Removed: None; Original threshold range: 1 – 502.44
Removed: Acrocephalidae
Thresholds 1 – 4 (12)
Mean # iterations significant: 15/20
Thresholds 4 – 9 (12)
Mean # iterations significant: 20/20
Thresholds 9 – 502.44 (12)
Mean # iterations significant: 16.1/20
Removed: Acrocephalidae; Original threshold range: 1 – 5
Removed: Icteridae
Thresholds 1 – 4 (12)
Mean # iterations significant: 15/20
Thresholds 4 – 9.45 (13)
Mean # iterations significant: 20/20
Thresholds 9.45 – 502.44 (12)
Mean # iterations significant: 12.2/20
Removed: Icteridae; Original threshold range: 1 – 502.4
Removed: Motacillidae
Thresholds 1 – 4 (12)
Mean # iterations significant: 16.3/20
Thresholds 4 – 9.45 (13)
Mean # iterations significant: 20/20
Thresholds 9.45 – 502.44 (12)
Mean # iterations significant: 11.9/20
Removed: Motacillidae; Original threshold range: 1 – 502
Removed: Cardinalidae
Thresholds 1 – 4.5 (12)
Mean # iterations significant: 18.3/20
Thresholds 4.5 – 9.45 (12)
Mean # iterations significant: 20/20
Thresholds 9.45 – 502.44 (12)
Mean # iterations significant: 12.4/20
Removed: Cardinalidae; Original threshold range: 1 – 50
Removed: Fringillidae
Thresholds 1 – 4 (12)
Mean # iterations significant: 15/20
Thresholds 4 – 9 (13)
Mean # iterations significant: 20/20
Thresholds 9 – 502.44 (12)
Mean # iterations significant: 12.1/20
Removed: Fringillidae; Original threshold range: 1 – 502
Removed: Certhiidae
Thresholds 1 – 4 (12)
Mean # iterations significant: 16.3/20
Thresholds 4 – 9 (13)
Mean # iterations significant: 20/20
Thresholds 9 – 502.44 (13)
Mean # iterations significant: 12.8/20
Removed: Certhiidae; Original threshold range: 1 – 502
Song−stable
Low Syllsong
Medium/High Syllsong
Low/Medium Syllsong
High Syllsong
Song−plastic
p−value
High/Low Threshold: Syllsong

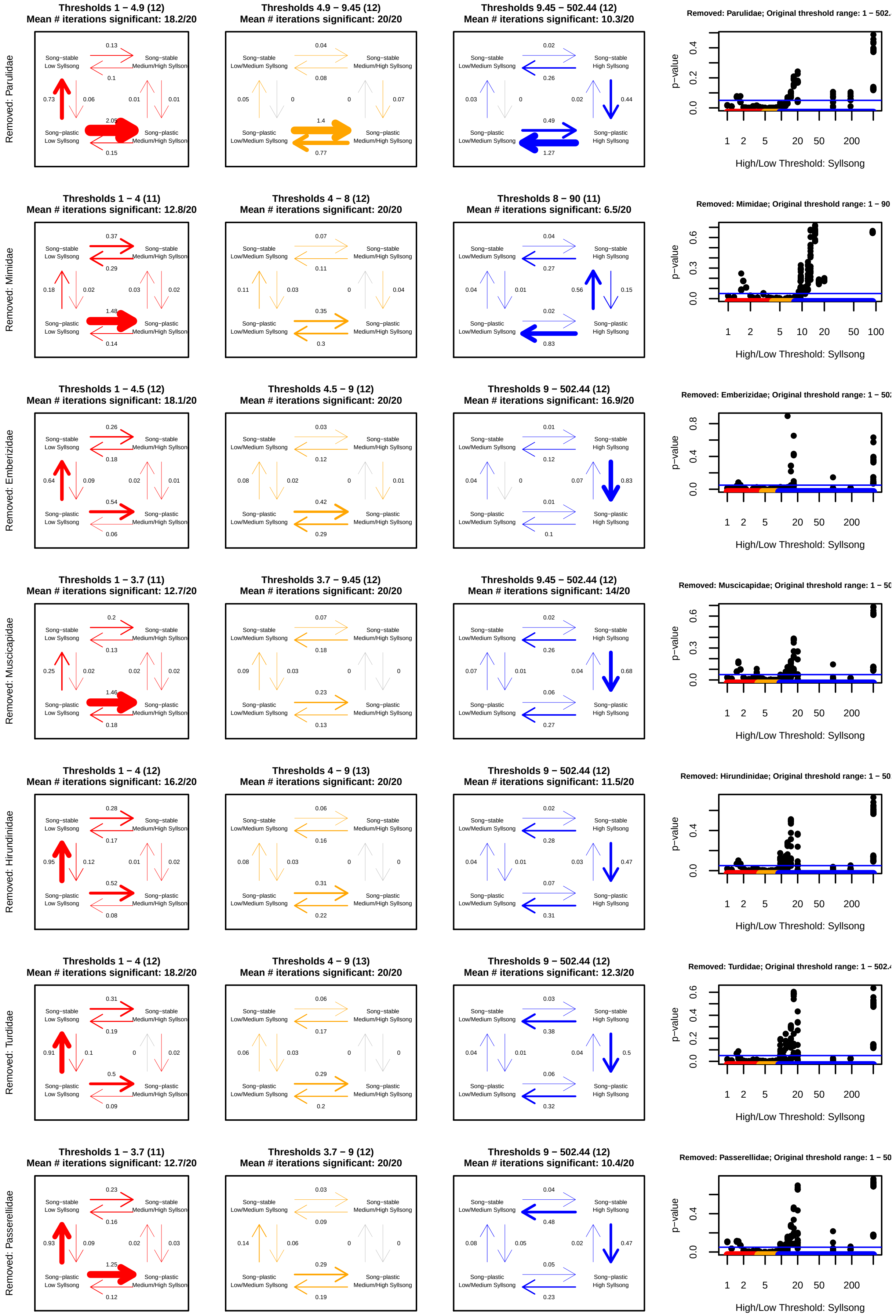
Removed: Parulidae
Thresholds 1 – 4.9 (12)
Mean # iterations significant: 18.2/20
Song−stable Low Syllsong
Song−stable Medium/High Syllsong
Song−plastic Low Syllsong
Song−plastic Medium/High Syllsong
0.13 0.1 0.73 0.06 0.01 0.01 2.05 0.15
Thresholds 4.9 – 9.45 (12)
Mean # iterations significant: 20/20
Song−stable Low/Medium Syllsong
Song−stable Medium/High Syllsong
Song−plastic Low/Medium Syllsong
Song−plastic Medium/High Syllsong
0.04 0.08 0.05 0 0 0.07 1.4 0.77
Thresholds 9.45 – 502.44 (12)
Mean # iterations significant: 10.3/20
Song−stable Low/Medium Syllsong
Song−stable High Syllsong
Song−plastic Low/Medium Syllsong
Song−plastic High Syllsong
0.02 0.26 0.03 0 0.02 0.44 0.49 1.27
Removed: Parulidae; Original threshold range: 1 – 502.
p−value
0.0 0.2 0.4
1 2 5 20 50 200
High/Low Threshold: Syllsong
Removed: Mimidae
Thresholds 1 – 4 (11)
Mean # iterations significant: 12.8/20
0.37 0.29 0.18 0.02 0.03 0.02 1.48 0.14
Thresholds 4 – 8 (12)
Mean # iterations significant: 20/20
0.07 0.11 0.11 0.03 0 0.04 0.35 0.3
Thresholds 8 – 90 (11)
Mean # iterations significant: 6.5/20
0.04 0.27 0.04 0.01 0.56 0.15 0.02 0.83
Removed: Mimidae; Original threshold range: 1 – 90
p−value
0.0 0.3 0.6
1 2 5 10 20 50 100
High/Low Threshold: Syllsong
Removed: Emberizidae
Thresholds 1 – 4.5 (12)
Mean # iterations significant: 18.1/20
0.26 0.18 0.64 0.09 0.02 0.01 0.54 0.06
Thresholds 4.5 – 9 (12)
Mean # iterations significant: 20/20
0.03 0.12 0.08 0.02 0 0.01 0.42 0.29
Thresholds 9 – 502.44 (12)
Mean # iterations significant: 16.9/20
0.01 0.12 0.04 0 0.07 0.83 0.01 0.1
Removed: Emberizidae; Original threshold range: 1 – 50
p−value
0.0 0.4 0.8
1 2 5 20 50 200
High/Low Threshold: Syllsong
Removed: Muscicapidae
Thresholds 1 – 3.7 (11)
Mean # iterations significant: 12.7/20
0.2 0.13 0.25 0.02 0.02 0.02 1.46 0.18
Thresholds 3.7 – 9.45 (12)
Mean # iterations significant: 20/20
0.07 0.18 0.09 0.03 0 0 0.23 0.13
Thresholds 9.45 – 502.44 (12)
Mean # iterations significant: 14/20
0.02 0.26 0.07 0.01 0.04 0.68 0.06 0.27
Removed: Muscicapidae; Original threshold range: 1 – 50
p−value
0.0 0.3 0.6
1 2 5 20 50 200
High/Low Threshold: Syllsong
Removed: Hirundinidae
Thresholds 1 – 4 (12)
Mean # iterations significant: 16.2/20
0.28 0.17 0.95 0.12 0.01 0.02 0.52 0.08
Thresholds 4 – 9 (13)
Mean # iterations significant: 20/20
0.06 0.16 0.08 0.03 0 0 0.31 0.22
Thresholds 9 – 502.44 (12)
Mean # iterations significant: 11.5/20
0.02 0.28 0.04 0.01 0.03 0.47 0.07 0.31
Removed: Hirundinidae; Original threshold range: 1 – 50
p−value
0.0 0.4
1 2 5 20 50 200
High/Low Threshold: Syllsong
Removed: Turdidae
Thresholds 1 – 4 (12)
Mean # iterations significant: 18.2/20
0.31 0.19 0.91 0.1 0 0.02 0.5 0.09
Thresholds 4 – 9 (13)
Mean # iterations significant: 20/20
0.06 0.17 0.06 0.03 0 0 0.29 0.2
Thresholds 9 – 502.44 (12)
Mean # iterations significant: 12.3/20
0.03 0.38 0.04 0.01 0.04 0.5 0.06 0.32
Removed: Turdidae; Original threshold range: 1 – 502.
p−value
0.0 0.2 0.4 0.6
1 2 5 20 50 200
High/Low Threshold: Syllsong
Removed: Passerellidae
Thresholds 1 – 3.7 (11)
Mean # iterations significant: 12.7/20
0.23 0.16 0.93 0.09 0.02 0.03 1.25 0.12
Thresholds 3.7 – 9 (12)
Mean # iterations significant: 20/20
0.03 0.09 0.14 0.06 0 0 0.29 0.19
Thresholds 9 – 502.44 (12)
Mean # iterations significant: 10.4/20
0.04 0.48 0.08 0.05 0.02 0.47 0.05 0.23
Removed: Passerellidae; Original threshold range: 1 – 50
p−value
0.0 0.4
1 2 5 20 50 200
High/Low Threshold: Syllsong

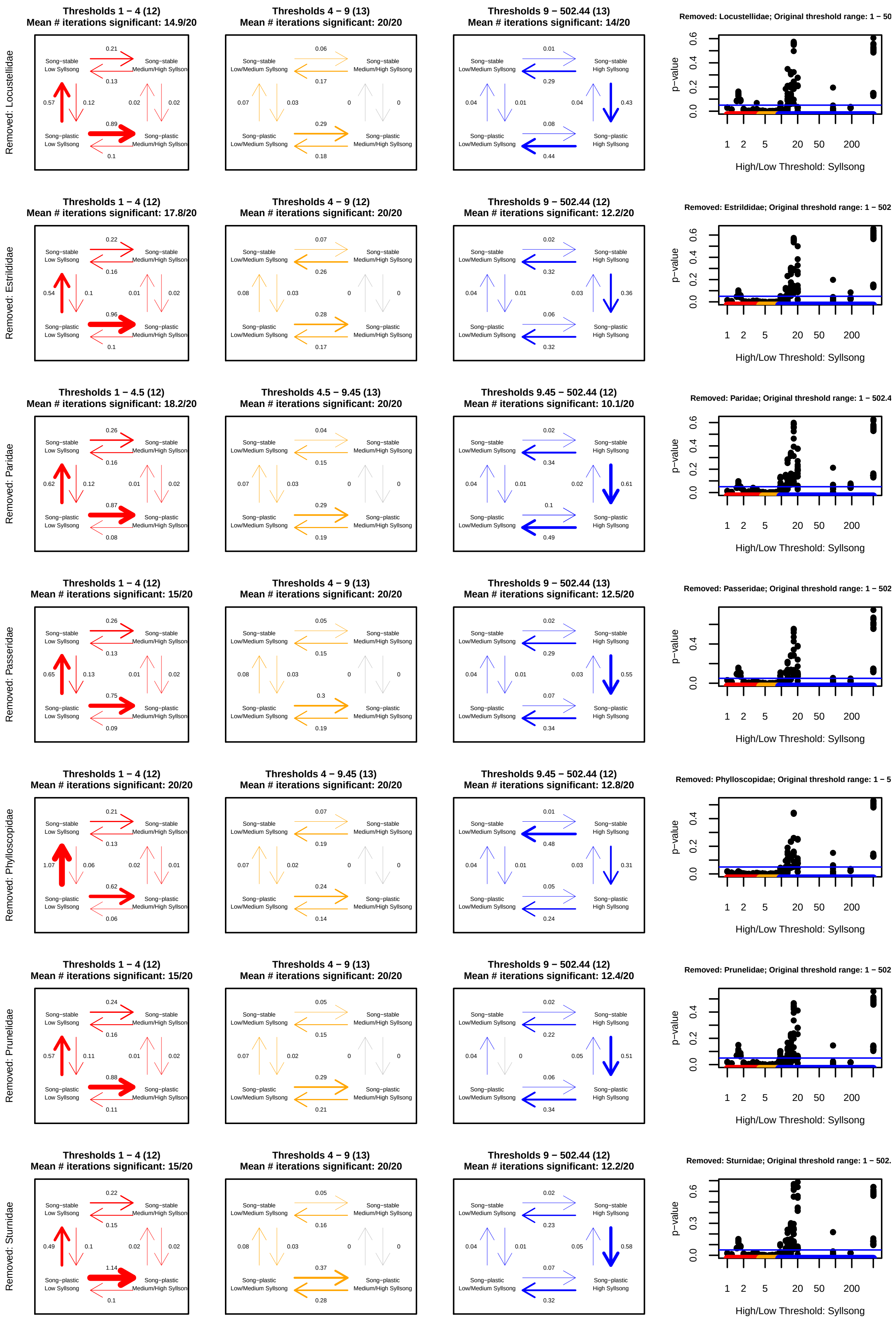

Removed: Locustellidae
Thresholds 1 – 4 (12)
Mean # iterations significant: 14.9/20
Thresholds 4 – 9 (13)
Mean # iterations significant: 20/20
Thresholds 9 – 502.44 (13)
Mean # iterations significant: 14/20
Removed: Locustellidae; Original threshold range: 1 – 50
Removed: Estrildidae
Thresholds 1 – 4 (12)
Mean # iterations significant: 17.8/20
Thresholds 4 – 9 (12)
Mean # iterations significant: 20/20
Thresholds 9 – 502.44 (12)
Mean # iterations significant: 12.2/20
Removed: Estrildidae; Original threshold range: 1 – 502
Removed: Paridae
Thresholds 1 – 4.5 (12)
Mean # iterations significant: 18.2/20
Thresholds 4.5 – 9.45 (13)
Mean # iterations significant: 20/20
Thresholds 9.45 – 502.44 (12)
Mean # iterations significant: 10.1/20
Removed: Paridae; Original threshold range: 1 – 502.4
Removed: Passeridae
Thresholds 1 – 4 (12)
Mean # iterations significant: 15/20
Thresholds 4 – 9 (13)
Mean # iterations significant: 20/20
Thresholds 9 – 502.44 (13)
Mean # iterations significant: 12.5/20
Removed: Passeridae; Original threshold range: 1 – 502
Removed: Phylloscopidae
Thresholds 1 – 4 (12)
Mean # iterations significant: 20/20
Thresholds 4 – 9.45 (13)
Mean # iterations significant: 20/20
Thresholds 9.45 – 502.44 (12)
Mean # iterations significant: 12.8/20
Removed: Phylloscopidae; Original threshold range: 1 – 5
Removed: Prunelidae
Thresholds 1 – 4 (12)
Mean # iterations significant: 15/20
Thresholds 4 – 9 (13)
Mean # iterations significant: 20/20
Thresholds 9 – 502.44 (12)
Mean # iterations significant: 12.4/20
Removed: Prunelidae; Original threshold range: 1 – 502
Removed: Sturnidae
Thresholds 1 – 4 (12)
Mean # iterations significant: 15/20
Thresholds 4 – 9 (13)
Mean # iterations significant: 20/20
Thresholds 9 – 502.44 (12)
Mean # iterations significant: 12.2/20
Removed: Sturnidae; Original threshold range: 1 – 502.
Song−stable
Low Syllsong
Medium/High Syllsong
Low/Medium Syllsong
High Syllsong
Song−plastic
p−value
High/Low Threshold: Syllsong

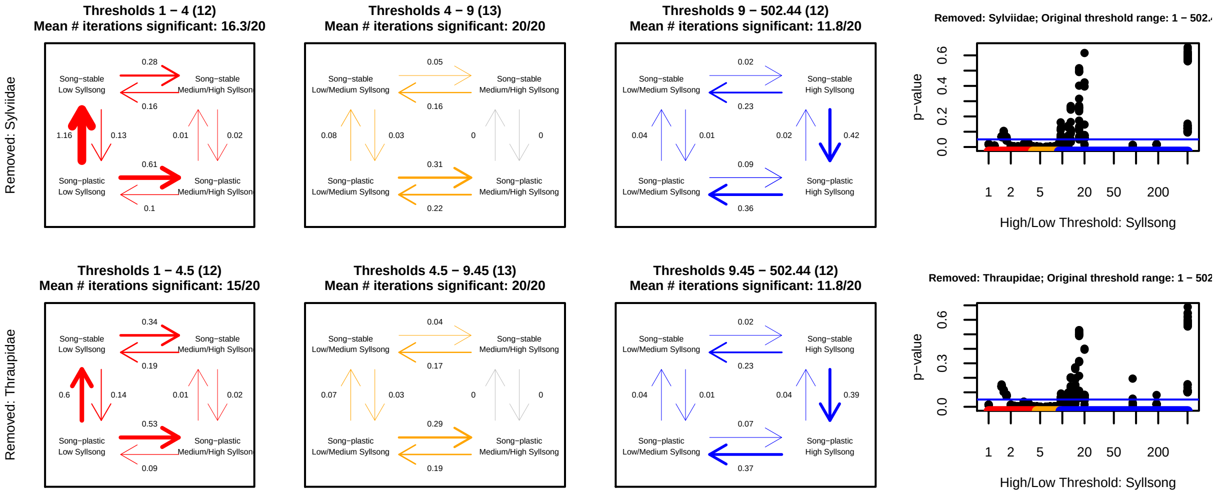
Removed: Sylviidae
Thresholds 1 − 4 (12)
Mean # iterations significant: 16.3/20
Song−stable Low Syllsong
Song−stable Medium/High Syllsong
Song−plastic Low Syllsong
Song−plastic Medium/High Syllsong
0.28
0.16
1.16
0.13
0.01
0.02
0.61
0.1
Thresholds 4 − 9 (13)
Mean # iterations significant: 20/20
Song−stable Low/Medium Syllsong
Song−stable Medium/High Syllsong
Song−plastic Low/Medium Syllsong
Song−plastic Medium/High Syllsong
0.05
0.16
0.08
0.03
0
0
0.31
0.22
Thresholds 9 − 502.44 (12)
Mean # iterations significant: 11.8/20
Song−stable Low/Medium Syllsong
Song−stable High Syllsong
Song−plastic Low/Medium Syllsong
Song−plastic High Syllsong
0.02
0.23
0.04
0.01
0.02
0.42
0.09
0.36
Removed: Sylviidae; Original threshold range: 1 − 502.4
p−value
0.0
0.2
0.4
0.6
1
2
5
20
50
200
High/Low Threshold: Syllsong
Removed: Thraupidae
Thresholds 1 − 4.5 (12)
Mean # iterations significant: 15/20
Song−stable Low Syllsong
Song−stable Medium/High Syllsong
Song−plastic Low Syllsong
Song−plastic Medium/High Syllsong
0.34
0.19
0.6
0.14
0.01
0.02
0.53
0.09
Thresholds 4.5 − 9.45 (13)
Mean # iterations significant: 20/20
Song−stable Low/Medium Syllsong
Song−stable Medium/High Syllsong
Song−plastic Low/Medium Syllsong
Song−plastic Medium/High Syllsong
0.04
0.17
0.07
0.03
0
0
0.29
0.19
Thresholds 9.45 − 502.44 (12)
Mean # iterations significant: 11.8/20
Song−stable Low/Medium Syllsong
Song−stable High Syllsong
Song−plastic Low/Medium Syllsong
Song−plastic High Syllsong
0.02
0.23
0.04
0.01
0.04
0.39
0.07
0.37
Removed: Thraupidae; Original threshold range: 1 − 502
p−value
0.0
0.3
0.6
1
2
5
20
50
200
High/Low Threshold: Syllsong
