# Supplementary material for: Correlated evolution between repertoire size and song plasticity predicts that sexual selection on song promotes open-ended learning
Source: eLife. 2019 Sep 3;8:e44454. doi: 10.7554/eLife.44454 (PMC6721395; doi:10.7554/eLife.44454)
Supplement: Figure 7—source data 1. — Labeling the same as in Figure 6 and Figure 6—figure supplement 2. [file elife-44454-fig7-data1.pdf]

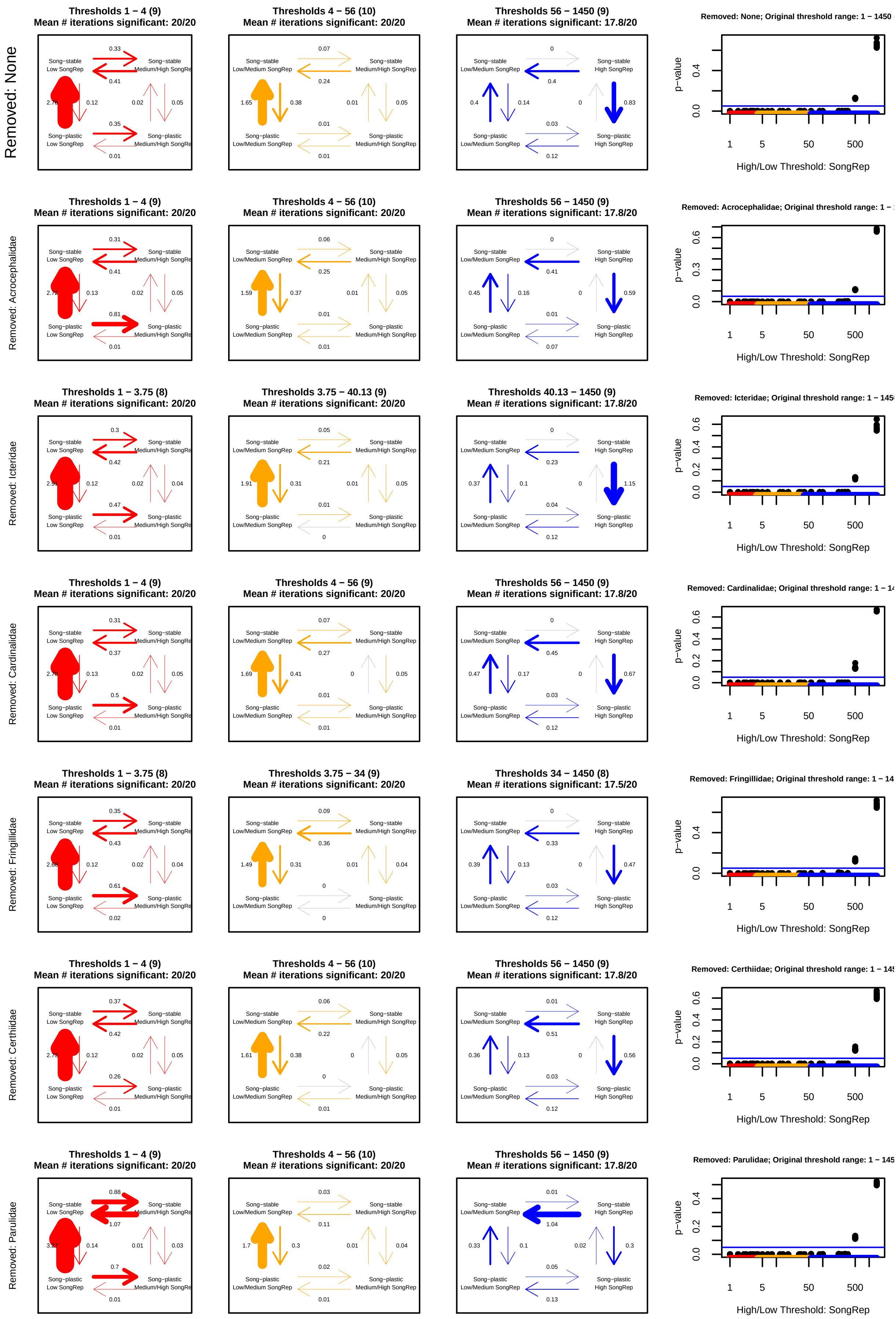

Removed: None
Thresholds 1 – 4 (9)
Mean # iterations significant: 20/20
Thresholds 4 – 56 (10)
Mean # iterations significant: 20/20
Thresholds 56 – 1450 (9)
Mean # iterations significant: 17.8/20
Removed: None; Original threshold range: 1 – 1450
Removed: Acrocephalidae
Thresholds 1 – 4 (9)
Mean # iterations significant: 20/20
Thresholds 4 – 56 (10)
Mean # iterations significant: 20/20
Thresholds 56 – 1450 (9)
Mean # iterations significant: 17.8/20
Removed: Acrocephalidae; Original threshold range: 1 –
Removed: Icteridae
Thresholds 1 – 3.75 (8)
Mean # iterations significant: 20/20
Thresholds 3.75 – 40.13 (9)
Mean # iterations significant: 20/20
Thresholds 40.13 – 1450 (9)
Mean # iterations significant: 17.8/20
Removed: Icteridae; Original threshold range: 1 – 145
Removed: Cardinalidae
Thresholds 1 – 4 (9)
Mean # iterations significant: 20/20
Thresholds 4 – 56 (9)
Mean # iterations significant: 20/20
Thresholds 56 – 1450 (9)
Mean # iterations significant: 17.8/20
Removed: Cardinalidae; Original threshold range: 1 – 14
Removed: Fringillidae
Thresholds 1 – 3.75 (8)
Mean # iterations significant: 20/20
Thresholds 3.75 – 34 (9)
Mean # iterations significant: 20/20
Thresholds 34 – 1450 (8)
Mean # iterations significant: 17.5/20
Removed: Fringillidae; Original threshold range: 1 – 14
Removed: Certhiidae
Thresholds 1 – 4 (9)
Mean # iterations significant: 20/20
Thresholds 4 – 56 (10)
Mean # iterations significant: 20/20
Thresholds 56 – 1450 (9)
Mean # iterations significant: 17.8/20
Removed: Certhiidae; Original threshold range: 1 – 145
Removed: Parulidae
Thresholds 1 – 4 (9)
Mean # iterations significant: 20/20
Thresholds 4 – 56 (10)
Mean # iterations significant: 20/20
Thresholds 56 – 1450 (9)
Mean # iterations significant: 17.8/20
Removed: Parulidae; Original threshold range: 1 – 145
p−value
High/Low Threshold: SongRep
1
5
50
500

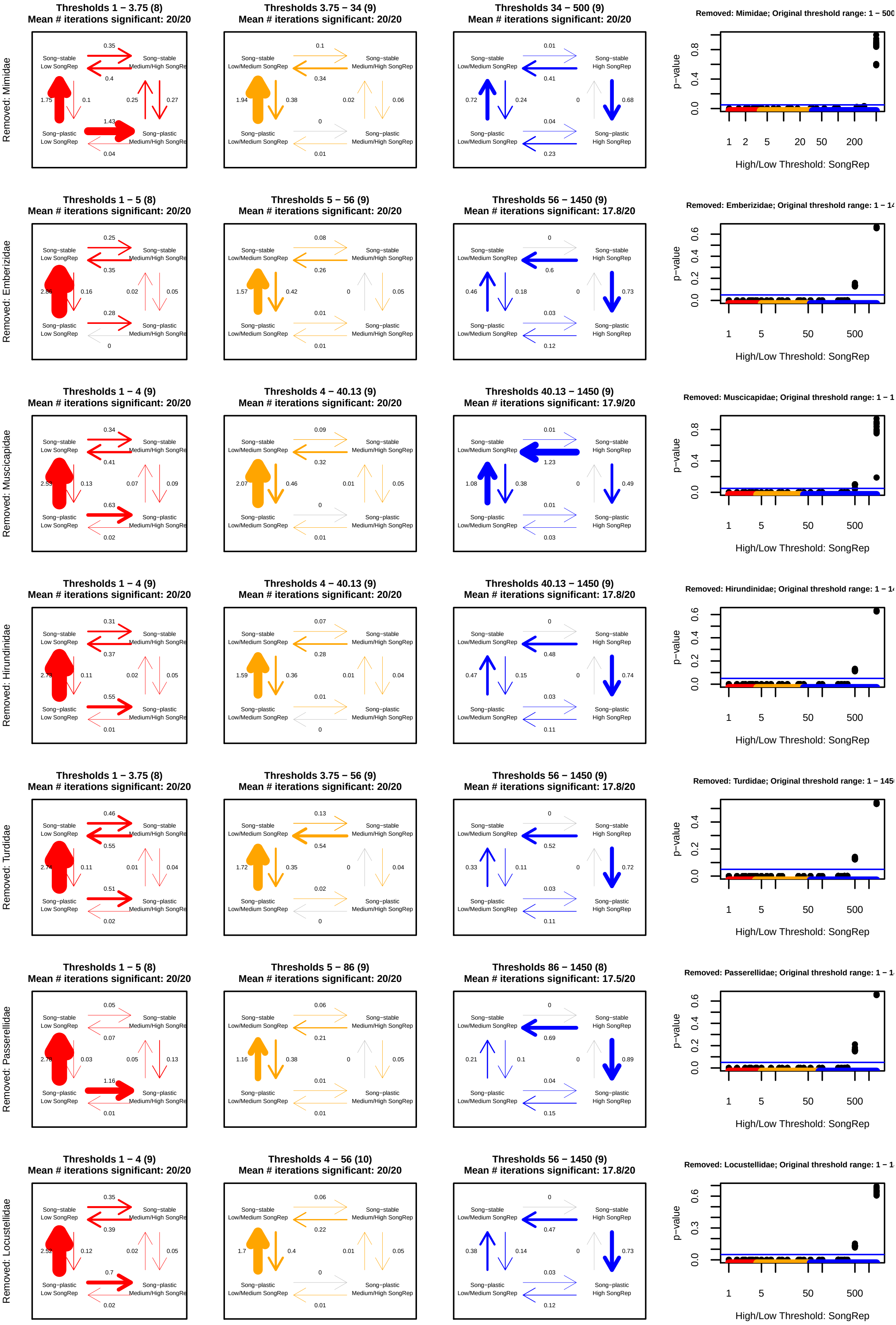
Thresholds 1 − 3.75 (8)
Mean # iterations significant: 20/20
Thresholds 3.75 − 34 (9)
Mean # iterations significant: 20/20
Thresholds 34 − 500 (9)
Mean # iterations significant: 20/20
Removed: Mimidae; Original threshold range: 1 − 500
Removed: Mimidae
Thresholds 1 − 5 (8)
Mean # iterations significant: 20/20
Thresholds 5 − 56 (9)
Mean # iterations significant: 20/20
Thresholds 56 − 1450 (9)
Mean # iterations significant: 17.8/20
Removed: Emberizidae
Thresholds 1 − 4 (9)
Mean # iterations significant: 20/20
Thresholds 4 − 40.13 (9)
Mean # iterations significant: 20/20
Thresholds 40.13 − 1450 (9)
Mean # iterations significant: 17.9/20
Removed: Muscicapidae
Thresholds 1 − 4 (9)
Mean # iterations significant: 20/20
Thresholds 4 − 40.13 (9)
Mean # iterations significant: 20/20
Thresholds 40.13 − 1450 (9)
Mean # iterations significant: 17.8/20
Removed: Hirundinidae
Thresholds 1 − 3.75 (8)
Mean # iterations significant: 20/20
Thresholds 3.75 − 56 (9)
Mean # iterations significant: 20/20
Thresholds 56 − 1450 (9)
Mean # iterations significant: 17.8/20
Removed: Turdidae
Thresholds 1 − 5 (8)
Mean # iterations significant: 20/20
Thresholds 5 − 86 (9)
Mean # iterations significant: 20/20
Thresholds 86 − 1450 (8)
Mean # iterations significant: 17.5/20
Removed: Passerellidae
Thresholds 1 − 4 (9)
Mean # iterations significant: 20/20
Thresholds 4 − 56 (10)
Mean # iterations significant: 20/20
Thresholds 56 − 1450 (9)
Mean # iterations significant: 17.8/20
Removed: Locustellidae
Song−stable
Song−plastic
Low SongRep
Low/Medium SongRep
Medium/High SongRep
High SongRep
p−value
High/Low Threshold: SongRep

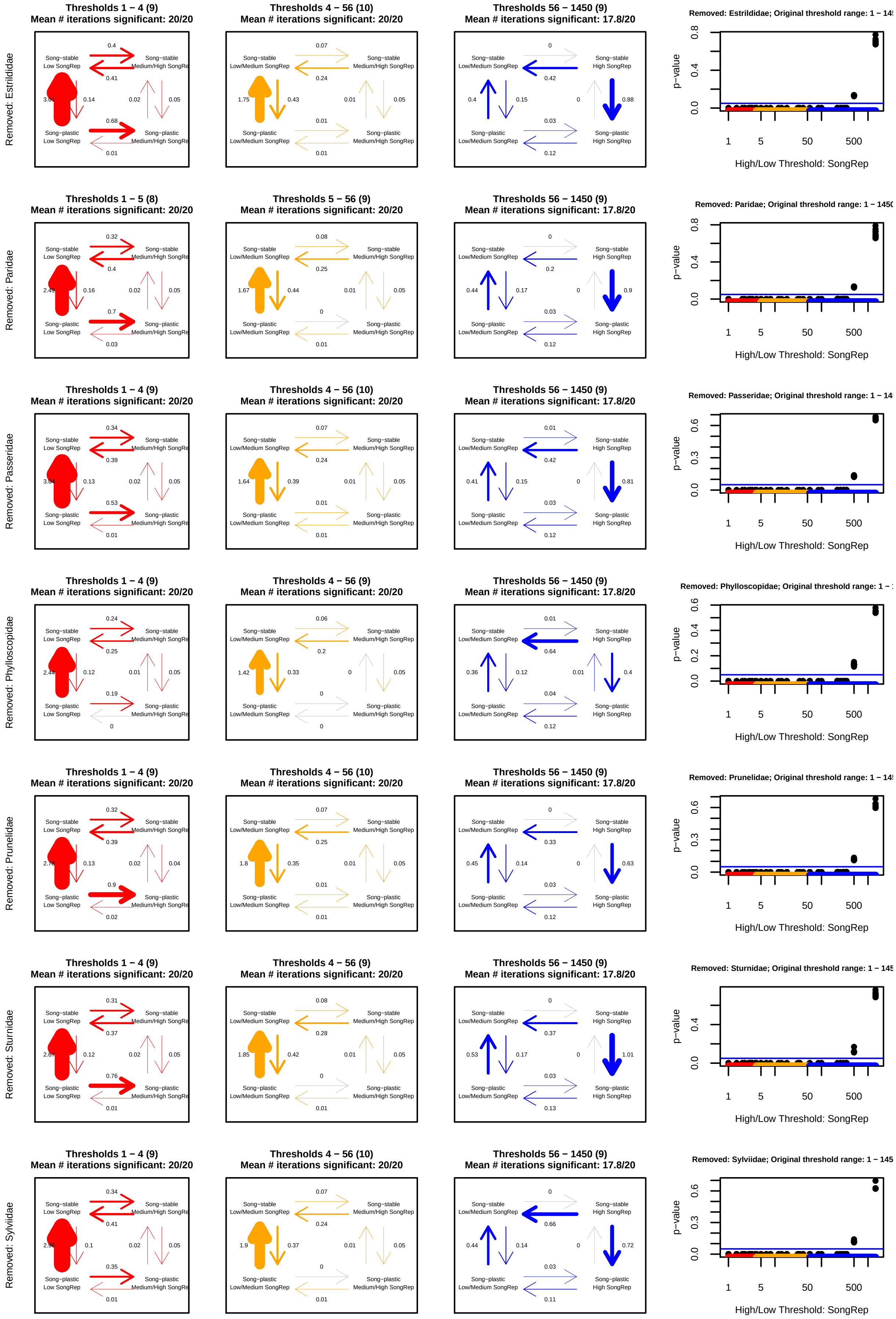
Removed: Estrildidae
Thresholds 1 – 4 (9)
Mean # iterations significant: 20/20
Thresholds 4 – 56 (10)
Mean # iterations significant: 20/20
Thresholds 56 – 1450 (9)
Mean # iterations significant: 17.8/20
Removed: Estrildidae; Original threshold range: 1 – 14
Removed: Paridae
Thresholds 1 – 5 (8)
Mean # iterations significant: 20/20
Thresholds 5 – 56 (9)
Mean # iterations significant: 20/20
Thresholds 56 – 1450 (9)
Mean # iterations significant: 17.8/20
Removed: Paridae; Original threshold range: 1 – 1450
Removed: Passeridae
Thresholds 1 – 4 (9)
Mean # iterations significant: 20/20
Thresholds 4 – 56 (10)
Mean # iterations significant: 20/20
Thresholds 56 – 1450 (9)
Mean # iterations significant: 17.8/20
Removed: Passeridae; Original threshold range: 1 – 14
Removed: Phylloscopidae
Thresholds 1 – 4 (9)
Mean # iterations significant: 20/20
Thresholds 4 – 56 (9)
Mean # iterations significant: 20/20
Thresholds 56 – 1450 (9)
Mean # iterations significant: 17.8/20
Removed: Phylloscopidae; Original threshold range: 1 –
Removed: Prunelidae
Thresholds 1 – 4 (9)
Mean # iterations significant: 20/20
Thresholds 4 – 56 (10)
Mean # iterations significant: 20/20
Thresholds 56 – 1450 (9)
Mean # iterations significant: 17.8/20
Removed: Prunelidae; Original threshold range: 1 – 14
Removed: Sturnidae
Thresholds 1 – 4 (9)
Mean # iterations significant: 20/20
Thresholds 4 – 56 (9)
Mean # iterations significant: 20/20
Thresholds 56 – 1450 (9)
Mean # iterations significant: 17.8/20
Removed: Sturnidae; Original threshold range: 1 – 145
Removed: Sylviidae
Thresholds 1 – 4 (9)
Mean # iterations significant: 20/20
Thresholds 4 – 56 (10)
Mean # iterations significant: 20/20
Thresholds 56 – 1450 (9)
Mean # iterations significant: 17.8/20
Removed: Sylviidae; Original threshold range: 1 – 145
Song−stable
Song−plastic
Low SongRep
Medium/High SongRep
Low/Medium SongRep
High SongRep
p−value
High/Low Threshold: SongRep

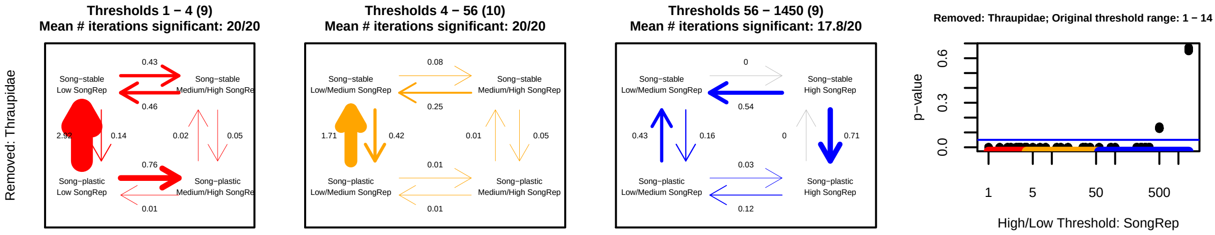
Removed: Thraupidae
Thresholds 1 − 4 (9)
Mean # iterations significant: 20/20
Song−stable
Low SongRep
Song−stable
Medium/High SongRep
Song−plastic
Low SongRep
Song−plastic
Medium/High SongRep
0.43
0.46
2.92
0.14
0.02
0.05
0.76
0.01
Thresholds 4 − 56 (10)
Mean # iterations significant: 20/20
Song−stable
Low/Medium SongRep
Song−stable
Medium/High SongRep
Song−plastic
Low/Medium SongRep
Song−plastic
Medium/High SongRep
0.08
0.25
1.71
0.42
0.01
0.05
0.01
0.01
Thresholds 56 − 1450 (9)
Mean # iterations significant: 17.8/20
Song−stable
Low/Medium SongRep
Song−stable
High SongRep
Song−plastic
Low/Medium SongRep
Song−plastic
High SongRep
0
0.54
0.43
0.16
0
0.71
0.03
0.12
Removed: Thraupidae; Original threshold range: 1 − 14
p−value
0.0
0.3
0.6
1
5
50
500
High/Low Threshold: SongRep
