# Supplementary material for: Correlated evolution between repertoire size and song plasticity predicts that sexual selection on song promotes open-ended learning
Source: eLife. 2019 Sep 3;8:e44454. doi: 10.7554/eLife.44454 (PMC6721395; doi:10.7554/eLife.44454)
Supplement: Source data 1. [file elife-44454-data1.docx]

| **Species** | **Stability** | **Reference** |
| --- | --- | --- |
| *Acrocephalus arundinaceus* | Plastic | [1], [2] |
| *Acrocephalus palustris* | Stable | [3] |
| *Acrocephalus schoenobaenus* | Plastic | [4] |
| *Acrocephalus stentoreus* | Plastic | [5] |
| *Agelaius phoeniceus* | Plastic | [6], [7] |
| *Anthus trivialis* | Stable | [8] |
| *Cacicus cela* | Stable | [9] |
| *Cardinalis cardinalis* | Stable | [10] |
| *Carduelis chloris* | Plastic | [11] |
| *Carpodacus cassinii* | Stable | [12] |
| *Carpodacus erythrinus* | Stable | [13], [14] However [15] |
| *Certhia familiaris* | Stable | [16] |
| *Dendroica pensylvanica* | Plastic | [17] |
| *Dendroica petechia* | Stable | [18] |
| *Dumetella carolinensis* | Plastic | [19] |
| *Emberiza citrinella* | Stable | [20] |
| *Emberiza hortulana* | Stable | [21], [22] However [23] |
| *Ficedula albicollis* | Plastic | [24] |
| *Ficedula hypoleuca* | Plastic | [25] |
| *Fringilla coelebs* | Stable | [26] |
| *Geospiza fortis* | Stable | [27] |
| *Geospiza scandens* | Stable | [27] |
| *Geothlypis trichas* | Stable | [28] |
| *Hirundo rustica* | Plastic | [29], [30] |
| *Hylocichla mustelina* | Stable | [31] |
| *Junco hyemalis* | Stable | [32] |
| *Lamprotornis superbus* | Plastic | [33] |
| *Locustella naevia* | Stable | [34] |
| *Lonchura striata* | Stable | [35] |
| *Luscinia megarhynchos* | Plastic | [36] |
| *Melospiza georgiana* | Stable | [37] |
| *Melospiza lincolnii* | Plastic | [38] |
| *Melospiza melodia* | Stable | [39] |
| *Miliaria calandra* | Stable | [40] |
| *Mimus gilvus* | Plastic | [41] |
| *Mimus polyglottos* | Plastic | [42] |
| *Molothrus ater* | Stable | [43] |
| *Padda oryzivora* | Stable | [44] |
| *Parus atricapillus* | Stable | [45] |
| *Parus major* | Stable | [46] |
| *Parus palustris* | Stable | [47] |
| *Passer domesticus* | Stable | [48] |
| *Passerculus sandwichensis* | Stable | [49] |
| *Passerella iliaca* | Stable | [50] |
| *Passerina cyanea* | Stable | [51] |
| *Philesturnus rufusater* | Plastic | [52] |
| *Phoenicurus ochruros* | Stable | [53] |
| *Phylloscopus collybita* | Stable | [54] |
| *Phylloscopus fuscatus* | Plastic | [55], [56] |
| *Phylloscopus trochilus* | Plastic | [57] |
| *Pipilo erythrophthalmus* | Stable | [58] |
| *Prunella modularis* | Plastic | [59] |
| *Serinus canaria* | Plastic | [60] |
| *Serinus serinus* | Plastic | [61] |
| *Setophaga ruticilla* | Plastic | [62] |
| *Spizella passerina* | Stable | [63] |
| *Spizella pusilla* | Stable | [64] |
| *Sturnella neglecta* | Plastic | [65] |
| *Sturnus vulgaris* | Plastic | [66], [67] |
| *Sylvia communis* | Plastic | [68] |
| *Taeniopygia guttata* | Stable | [69] |
| *Toxostoma rufum* | Plastic | [70] |
| *Turdus grayi* | Plastic | [71] |
| *Turdus merula* | Plastic | [72] |
| *Zonotrichia albicollis* | Stable | [73] |
| *Zonotrichia leucophrys* | Stable | [74] |
| *Zosterops lateralis* | Plastic | [75] |
|  |  |  |
|  |  |  |

[1] D. Hasselquist, “Polygyny in Great Reed Warblers: a Long-Term Study of Factors Contributing To Male Fitness,” *Ecology*, vol. 79, no. 7, pp. 2376–2390, 1998.

[2] S. Nowicki, D. Hasselquist, S. Bensch, and S. Peters, “Nestling growth and song repertoire size in great reed warblers: evidence for song learning as an indicator mechanism in mate choice.,” *Proc. Biol. Sci.*, vol. 267, no. 1460, pp. 2419–24, 2000.

[3] F. Dowsett-Lemaire, “The imitative range of the song of the marsh warbler *Acrocephalus palustris*, with special reference to imitations of african birds,” *Ibis (Lond. 1859).*, vol. 121, no. 4, pp. 453–468, 1979.

[4] J. S. Nicholson, K. L. Buchanan, R. C. Marshall, and C. K. Catchpole, “Song sharing and repertoire size in the sedge warbler, *Acrocephalus schoenobaenus*: Changes within and between years,” *Anim. Behav.*, 2007.

[5] A. S. Opaev and V. V. Ivanitskii, “Advertising vocalization of the clamorous reed warbler (Acrocephalus stentoreus, Sylviidae),” *Biol. Bull.*, vol. 37, no. 8, pp. 768–779, 2010.

[6] K. Yasukawa, J. L. Blank, and C. B. Patterson, “Song repertoires and sexual selection in the red-winged blackbird,” *Behav. Ecol. Sociobiol.*, vol. 7, no. 3, pp. 233–238, 1980.

[7] B. Y. P. Marler, P. Mundinger, M. S. U. E. Waser, and A. N. N. Lutjen, “Song development in red-winged blackbirds (*Agelaius phoeniceus*),” *Anim. Behav.*, pp. 586–606, 1972.

[8] T. Petrusková, T. S. Osiejuk, and A. Petrusek, “Geographic variation in songs of the tree pipit at two spatial scales,” *Auk*, vol. 127, no. 2, pp. 274–282, 2010.

[9] J. M. Trainer and R. J. Parsons, “Delayed Vocal Maturation in Polygynous Yellow-Rumped Caciques,” *Wilson Bull.*, vol. 114, no. 2, pp. 249–254, 2002.

[10] R. E. Lemon, “Geographic variation in the song of cardinals,” *Can. J. Zool.*, 1966.

[11] H. R. Güttinger, “The Integration of Learnt and Genetically Programmed Behaviour: A Study of Hierarchical Organization in Songs of Canaries, Greenfinches and their Hybrids,” *Z. Tierpsychol.*, vol. 49, no. 3, pp. 285–303, 1979.

[12] F. B. Samson, “Vocalizations of Cassin’s Finch in Northern Utah,” *Condor*, vol. 80, no. 2, p. 203, 1978.

[13] M. Bjorklund, “Reproductive Success in the Common Rosefinch ( Carpodacus Erythrinus ),” *Auk*, no. January, pp. 35–44, 1990.

[14] J. Martens and P. Kessler, “Territorial song and song neighbourhoods in the Scarlet Rosefinch Carpodacus erythrinus,” *J. Avian Biol.*, vol. 31, no. 3, pp. 399–411, 2000.

[15] M. Björklund, “Microgeographic Variation in the Song of the Scarlet Rosefinch Carpodacus erythrinus,” *Ornis Scand.*, 1989.

[16] G. Thielcke, “Lernen von Gesang als möglicher Schrittmacher der Evolution,” *J. Zool. Syst. Evol. Res.*, 1970.

[17] B. E. Byers, “Geographic variation of song form within and among chestnut-sided warbler populations,” *Auk*, vol. 113, no. 2, pp. 288–299, 1996.

[18] S. E. Cosens and S. G. Sealy, “Age-related variation in song repertoire size and repertoire sharing of yellow warblers ( Dendroica petechia ),” *Can. J. Zool.*, vol. 64, no. 9, pp. 1926–1929, 1986.

[19] L. E. Fletcher and D. G. Smith, “Some parameters of song important in conspecific recognition by Gray Catbirds,” *Auk*, 1978.

[20] P. Hansen, “Geographic song variation in the Yellowhammer (Emberiza citrinella),” *Nat. Jutl.*, 1985.

[21] T. S. Osiejuk, K. Ratyńska, J. P. Cygan, and S. Dale, “Frequency shift in homologue syllables of the Ortolan Bunting Emberiza hortulana,” *Behav. Processes*, vol. 68, no. 1, pp. 69–83, 2005.

[22] L. Wiener, “Song learning in birds: Possible models for human language acquisition,” WORD, vol. 37, no. 3, pp. 159–175, 1986.

[23] S. E. Cosens and S. G. Sealy, “Age-related variation in song repertoire size and repertoire sharing of yellow warblers ( *Dendroica petechia* ),” *Can. J. Zool.*, 1986.

[24] L. Z. Garamszegi, J. Török, G. Hegyi, E. Szöllõsi, B. Rosivall, and M. Eens, “Age-dependent expression of song in the collared flycatcher, *Ficedula albicollis*,” *Ethology*, vol. 113, no. 3, pp. 246–256, 2007.

[25] A. Eriksen, T. Slagsvold, and H. M. Lampe, “Vocal plasticity - are pied flycatchers, *Ficedula Hypoleuca*, open-ended learners?,” *Ethology*, 2011.

[26] W. H. Thorpe, “THE LEARNING OF SONG PATTERNS BY BIRDS, WITH ESPECIAL REFERENCE TO THE SONG OF THE CHAFFINCH FRINGILLA COELEBS,” *Ibis (Lond. 1859).*, 1958.

[27] B. R. Grant and P. R. Grant, “Cultural Inheritance of Song and Its Role in the Evolution of Darwin’s Finches,” *Evolution (N. Y).*, vol. 50, no. 6, pp. 2471–2487, 1996.

[28] D. J. Borror, “Songs of the Yellowthroat,” *Living Bird*, vol. 6, pp. 141–161, 1967.

[29] L. Z. Garamszegi, D. Heylen, A. P. Møller, M. Eens, and F. De Lope, “Age-dependent health status and song characteristics in the barn swallow,” *Behav. Ecol.*, 2005.

[30] P. Galeotti, N. Saino, E. Perani, R. Sacchi, and A. R. Møller, “Age-related song variation in male barn swallows,” *Ital. J. Zool.*, vol. 68, no. 4, pp. 305–310, 2001.

[31] W. E. Lanyon, “Development of Song in the Wood Thrush ( Hylocichla mustelina ), With Notes on a Technique for Hand-rearing Passerines from the Egg,” *Am. Museum Novit.*, vol. 2666, pp. 1–27, 1979.

[32] R. C. Titus, E. D. Ketterson, and V. Nolan, “High testosterone prior to song crystallization inhibits singing behavior in captive yearling dark-eyed juncos (Junco hyemalis),” *Horm. Behav.*, 1997.

[33] S. Keen, C. D. Meliza, J. Pilowsky, and D. R. Rubenstein, “Song in a Social and Sexual Context: Vocalizations Signal Identity and Rank in Both Sexes of a Cooperative Breeder,” *Front. Ecol. Evol.*, vol. 4, no. May, pp. 1–9, 2016.

[34] P. Becker, “Song of the grasshopper warbler Locustella naevia in acoustic isolation,” *Vogelwarte*, vol. 35, pp. 257–267, 1990.

[35] N. S. Clayton, “Song learning in Bengalese finches: a comparison with zebra finches,” *Ethology*, vol. 76, no. 3, pp. 247–255, 1987.

[36] S. Kiefer *et al.*, “First-year common nightingales (Luscinia megarhynchos) have smaller song-type repertoire sizes than older males,” *Ethology*, vol. 112, no. 12, pp. 1217–1224, 2006.

[37] P. Marler and S. Peters, “Structural changes in song ontogeny in the swamp sparrow Melospiza georgiana,” *Auk*, 1982.

[38] C. Cicero and Z. M. Benowitz-Fredericks, “Song Types and Variation in Insular Populations of Lincoln’s Sparrow (Melospiza Lincolnii), and Comparisons with Other Melospiza,” *Auk*, vol. 117, no. 1, p. 52, 2000.

[39] J. C. Nordby, S. E. Campbell, and M. D. Beecher, “Adult song sparrows do not alter their song repertoires,” *Ethology*, 2002.

[40] P. K. McGregor, *Playback and studies of animal communication*. 1992.

[41] J. J. Price and D. Yuan, “Song-type sharing and matching in a bird with very large song repertoires, the tropical mockingbird,” *Behaviour*, vol. 148, pp. 673–689, 2011.

[42] R. D. Howard, “The influence of sexual selection and interspecific competition on mockingbird song (*Mimus polyglottus*),” *Evolution (N. Y).*, vol. 28, no. 3, pp. 428–438, 1974.

[43] A. L. O’Loghlen and S. I. Rothstein, “Ecological effects on song learning: Delayed development is widespread in wild populations of brown-headed cowbirds,” *Anim. Behav.*, 2002.

[44] N. Ota and M. Soma, “Age-dependent song changes in a closed-ended vocal learner: Elevation of song performance after song crystallization,” *J. Avian Biol.*, vol. 45, no. 6, pp. 566–573, 2014.

[45] S. a. Shackleton and L. Ratcliffe, “Development of Song in Hand-Reared Black-Capped Chickadees,” *Wilson Bull.*, vol. 105, no. 4, pp. 637–644, 1993.

[46] H. F. Rivera-Gutierrez, R. Pinxten, and M. Eens, “Difficulties when assessing birdsong learning programmes under field conditions: A re-evaluation of song repertoire flexibility in the great tit,” *PLoS One*, 2011.

[47] V. P. H. Becker, “Der Einfluß des Lernens auf einfache und komplexe Gesangsstrophen der Sumpfmeise ( Parus palustris),” *J. f{ü}r Ornithol.*, pp. 388–411, 1978.

[48] G. Wang, C. E. Harpole, J. Paulose, and V. M. Cassone, “The role of the pineal gland in the photoperiodic control of bird song frequency and repertoire in the house sparrow, Passer domesticus,” *Horm. Behav.*, vol. 65, no. 4, pp. 372–379, 2014.

[49] N. T. Wheelwright, M. B. Swett, I. I. Levin, D. E. Kroodsma, C. R. Freeman-Gallant, and H. Williams, “The influence of different tutor types on song learning in a natural bird population,” *Anim. Behav.*, vol. 75, no. 4, pp. 1479–1493, 2008.

[50] D. J. Martin, “Songs of the Fox Sparrow . I . Structure of Song and Its Comparison With Song in Other Emberizidae,” *Condor*, vol. 79, pp. 209–221, 1977.

[51] R. B. Payne, “Ecological consequences of song matching: breeding success and intraspecific song mimicry in indigo buntings ( Passerina cyanea, Michigan).,” *Ecology*, 1982.

[52] P. F. Jenkins, “Cultural transmission of song patterns and dialect development in a free-living bird population,” *Anim. Behav.*, vol. 26, no. PART 1, pp. 50–78, 1978.

[53] T. I. Draganoiu, L. Ravaux, A. Moreau, N. Mathevon, and W. Bonckaert, “Song stability and neighbour recognition in a migratory songbird, the black redstart,” *Behaviour*, 2014.

[54] G. Thielcke and U. Zimmer, “Early Experience Determines the Song of the Chiffchaff (Phylloscopus collybita),” *Ethology*, 1986.

[55] W. Forstmeier, B. Kempenaers, A. Meyer, and B. Leisler, “A novel song parameter correlates with extra-pair paternity and reflects male longevity,” *Proc. Biol. Sci.*, vol. 269, no. 1499, pp. 1479–85, 2002.

[56] W. Forstmeier and T. J. S. Balsby, “Why mated dusky warblers sing so much: Territory guarding and male quality announcement,” *Behaviour*, 2002.

[57] D. Gil, J. L. S. Cobb, and P. J. B. Slater, “Song characteristics are age dependent in the willow warbler, Phylloscopus trochilus,” *Anim. Behav.*, 2001.

[58] G. T. Smith, E. A. Brenowitz, M. D. Beecher, and J. C. Wingfield, “Seasonal changes in testosterone, neural attributes of song control nuclei, and song structure in wild songbirds.,” *J. Neurosci.*, 1997.

[59] D. W. Snow and B. K. Snow, “Territorial song of the Dunnock Prunella modularis,” *Bird Study*, vol. 30, no. 1, pp. 51–56, 1983.

[60] F. Nottebohm and M. E. Nottebohm, “Relationship between Song Repertoire and Age in the Canary, Serinus canarius,” *Zeitschrift f??r Tierpsychologie*, 1978.

[61] A. T. Mamede and P. G. Mota, “Limited Inter-Annual Song Variation in the Serin (Serinus serinus),” *Ethology*, vol. 118, no. 12, pp. 1157–1164, 2012.

[62] R. E. L. C. W. D. P. G. Clifton, “Songs of American Redstarts (Setophaga ruticilla): Sequencing Rules and their Relationships to Repertoire Size,” *ethology*, vol. 93, no. 3, pp. 198–210, 1993.

[63] W. -c. Liu and F. Nottebohm, “A learning program that ensures prompt and versatile vocal imitation,” *Proc. Natl. Acad. Sci.*, 2007.

[64] D. A. Nelson, “Song overproduction and selective attrition lead to song sharing in the field sparrow (Spizella pusilla),” *Behav. Ecol. Sociobiol.*, 1992.

[65] A. Horn, “Structure of western meadowlark (Sturnella neglecta) song repertoires,” *Can. J. Zool.*, vol. 66, no. 2, pp. 284–288, 1988.

[66] D. J. Mountjoy and R. E. Lemon, “Extended song learning in wild European starlings,” *Anim. Behav.*, vol. 49, no. 2, pp. 357–366, 1995.

[67] M. Eens, R. Pinxten, and R. F. Verheyen, “Male song as a cue for mate choice in the European starling,” *Behaviour*, vol. 116, no. 3–4, pp. 210–238, 1991.

[68] T. J. S. Balsby and P. Hansen, “Element repertoire: Change and development with age in whitethroat *Sylvia communis* song,” *J. Ornithol.*, 2010.

[69] K. Immelmann, “Song development in the zebra finch and other estrildid finches,” in *Bird Vocalizations*, R. A. Hinde, Ed. Cambridge: Cambridge Univ Press, 1969, pp. 61–74.

[70] D. E. Kroodsma and L. D. Parker, “Vocal virtuosity in the Brown Thrasher,” *Auk*, 1977.

[71] L. E. Vargas-Castro, N. V. Sánchez, and G. Barrantes, “Repertoire size and syllable sharing in the song of the Clay-Colored Thrush (Turdus grayi),” *Wilson J. Ornithol.*, vol. 124, no. 3, pp. 446–453, 2012.

[72] N. Hesler, R. Mundry, and T. Dabelsteen, “Are there age-related differences in the song repertoire size of Eurasian blackbirds?,” *Acta Ethol.*, 2012.

[73] W. W. H. Gunn and D. J. BORROR, “VARIATION IN WHITE-THROATED SPARROW SONGS,” *Auk*, vol. 82, pp. 26–47, 1965.

[74] D. A. Nelson, P. Marler, and M. L. Morton, “Overproduction in song development: An evolutionary correlate with migration,” *Anim. Behav.*, 1996.

[75] M. C. Baker, “Silvereyes (Zosterops lateralis) Song Differentiation in an Island-Mainland Comparison: Analyses of a Complex Cultural Trait,” *Wilson J. Ornithol.*, vol. 124, no. 3, pp. 454–466, 2012.
