# Supplementary material for: Correlated evolution between repertoire size and song plasticity predicts that sexual selection on song promotes open-ended learning
Source: eLife. 2019 Sep 3;8:e44454. doi: 10.7554/eLife.44454 (PMC6721395; doi:10.7554/eLife.44454)
Supplement: Supplementary file 1. [file elife-44454-supp1.pdf]

Table S1: PhylANOVA results for song traits when either the maximum or minimum reported values are used and birds are divided into song-stable and song-plastic. Song traits are sorted from most to least significant. Song-Stable and Song-Plastic columns show means. Corrected α indicates the threshold for significance with the Holm-Bonferroni correction. * denotes traits with significantly different groups.

Song Trait	Song-Stable	Song-Plastic	F-Value	Corrected α	p-Value
Syllable Rep Min	1.8106	3.7433	39.2342	0.0071	<0.001*
Syllable Rep Max	1.9176	4.0608	41.99	0.0071	<0.001*
Song Rep Min	0.8721	3.3024	37.8962	0.0083	<0.001*
Song Rep Max	1.181	4.211	34.4445	0.0083	<0.001*
Syll Song Max	1.2971	2.47	9.714	0.01	0.087
Syll Song Min	1.1881	1.8088	5.6204	0.01	0.217
Duration Max	0.7895	1.3853	2.4515	0.0125	0.389
Interval Max	1.6908	1.2852	1.7593	0.025	0.512
Duration Min	0.7503	1.1234	1.3346	0.0125	0.54
Interval Min	1.4121	0.9398	0.8895	0.025	0.661

Table S2: PhylANOVA results for song traits when either the maximum or minimum reported values are used and birds are divided into early song-stable, delayed song-stable, and song-plastic. Song traits are sorted from most to least significant. Early, Delayed, and Plastic columns show means. Corrected α indicates the threshold for significance with the Holm-Bonferroni correction.* denotes traits with significantly different groups.

Song Trait	Early	Delayed	Plastic	F-Value	Corrected α	p-Value
Syllable Rep Min	1.5451	1.9802	3.7433	16.317	0.0071	0.003*
Syllable Rep Max	1.697	2.0233	4.0608	17.209	0.0071	0.002*
Song Rep Min	0.5887	1.1625	3.3024	14.2085	0.0083	0.006*
Song Rep Max	0.7427	1.5669	4.211	13.059	0.0083	0.011
Syll Song Min	1.1987	1.1694	1.8088	2.4789	0.01	0.398
Syll Song Max	1.3186	1.3015	2.47	3.7714	0.01	0.243

Table S3: Post-hoc pairwise phylANOVA tests for significant song traits when either the maximum or minimum reported values are used and birds are divided into early song-stable, delayed song-stable, and song-plastic. Corrected α indicates the threshold for significance with the Holm-Bonferroni correction. * denotes traits with significantly different groups.

Song Trait	State 1	State 2	T-Value	p-Value
Syllable Rep Min	Plastic	Delayed	4.6745	0.014*
Syllable Rep Min	Early	Plastic	4.6194	0.003*
Syllable Rep Min	Early	Delayed	0.8659	0.581
Syllable Rep Max	Plastic	Delayed	4.9639	0.01*
Syllable Rep Max	Early	Plastic	4.5646	0.003*
Syllable Rep Max	Early	Delayed	0.5967	0.697
Song Rep Min	Plastic	Delayed	4.3491	0.022*
Song Rep Min	Early	Plastic	4.4142	0.012*
Song Rep Min	Early	Delayed	0.8988	0.614

Table S4: Brownie results for song traits when either the maximum or minimum reported values are used and birds are divided into song-stable and song-plastic. Song traits are sorted from most to least significant. * denotes traits where the two-rate model fit the data significantly better than the one-rate model.

Song Trait	One Rate	Two Rates	p-Value
Interval Max	-43.8289	-38.1264	<0.001*
Duration Max	-74.1132	-68.1884	<0.001*
Syll Song Max	-119.0363	-105.4808	<0.001*
Duration Min	-65.7687	-62.6047	0.012*
Interval Min	-58.6807	-56.4811	0.036*
Syll Song Min	-88.5573	-87.264	0.108
Song Rep Max	-120.3993	-119.6825	0.231
Syllable Rep Max	-125.0196	-124.3595	0.251
Song Rep Min	-100.8545	-100.5988	0.475
Syllable Rep Min	-118.6497	-118.4524	0.53

Table S5: Brownie results for song traits when either the maximum or minimum reported values are used and birds are divided into early song-stable, delayed song-stable, and song-plastic. Song traits are sorted from most to least significant. * denotes traits where the three-rate model fit the data significantly better than the one-rate model.

Song Trait	One Rate	Three Rates	p-Value
Syll Song Max	-105.7229	-91.4766	<0.001*
Song Rep Max	-107.1249	-103.2302	0.005*
Syll Song Min	-75.3007	-72.5126	0.018*
Syllable Rep Min	-106.2125	-104.0387	0.037*
Syllable Rep Max	-111.8435	-109.7931	0.043*
Song Rep Min	-88.7243	-86.9529	0.06

Table S6: PhylANOVA results for syllable repertoire when each bird family is omitted. Song traits are sorted from most to least significant. Song-Stable and Song-Plastic columns show means. Corrected α indicates the threshold for significance with the Holm-Bonferroni correction. * denotes significantly different groups.

Removed Family	Song-Stable	Song-Plastic	F-Value	Corrected α	p-Value
Acrocephalidae	1.8266	4.0232	47.6941	0.0071	<0.001*
Icteridae	1.8195	3.9652	40.9489	0.0071	<0.001*
Mimidae	1.8807	3.5707	32.426	0.0071	<0.001*
Muscicapidae	1.888	3.8686	36.7766	0.0071	<0.001*
Parulidae	1.8817	4.099	46.2305	0.0071	<0.001*
Passerellidae	1.9468	3.9792	32.4733	0.0071	<0.001*
Fringillidae	1.8177	3.9474	37.5394	0.0071	0.003*

Table S7: PhylANOVA results for interval when each bird family is omitted. Song traits are sorted from most to least significant. Song-Stable and Song-Plastic columns show means. Corrected α indicates the threshold for significance with the Holm-Bonferroni correction. * denotes significantly different groups.

Removed Family	Song-Stable	Song-Plastic	F-Value	Corrected α	p-Value
Muscicapidae	1.6075	1.0449	2.6556	0.025	0.326
Acrocephalidae	1.8406	1.3251	3.0278	0.025	0.404
Icteridae	1.6075	1.0659	2.5898	0.025	0.479
Parulidae	1.5815	1.1387	1.4568	0.025	0.595
Fringillidae	1.5821	1.218	1.1582	0.025	0.628
Passerellidae	1.4435	1.218	0.3135	0.025	0.725
Mimidae	1.6075	1.6071	0	0.025	1

Table S8: PhylANOVA results for duration when each bird family is omitted. Song traits are sorted from most to least significant. Song-Stable and Song-Plastic columns show means. Corrected α indicates the threshold for significance with the Holm-Bonferroni correction. * denotes significantly different groups.

Removed Family	Song-Stable	Song-Plastic	F-Value	Corrected α	p-Value
Mimidae	0.7736	1.5596	4.8068	0.0125	0.155
Muscicapidae	0.7736	1.3495	2.183	0.0125	0.354
Passerellidae	0.8056	1.2927	1.1871	0.0125	0.403
Acrocephalidae	0.62	1.1605	2.3871	0.0125	0.409
Parulidae	0.7993	1.4276	2.6748	0.0125	0.421
Icteridae	0.7736	1.3928	2.7298	0.0125	0.435
Fringillidae	0.7747	0.9735	0.4281	0.0125	0.784

Table S9: PhylANOVA results for syllables per song when each bird family is omitted. Song traits are sorted from most to least significant. Song-Stable and Song-Plastic columns show means. Corrected α indicates the threshold for significance with the Holm-Bonferroni correction. * denotes significantly different groups.

Removed Family	Song-Stable	Song-Plastic	F-Value	Corrected α	p-Value
Muscicapidae	1.2556	2.342	8.7962	0.01	0.086
Acrocephalidae	1.1915	2.3503	11.5839	0.01	0.091
Parulidae	1.2432	2.4329	10.9929	0.01	0.095
Passerellidae	1.3391	2.2962	5.8932	0.01	0.1
Fringillidae	1.2335	2.3554	9.4058	0.01	0.119
Icteridae	1.2556	2.3636	9.7574	0.01	0.13
Mimidae	1.2556	1.792	4.3591	0.01	0.222

Table S10: PhylANOVA results for song rate when each bird family is omitted. Song traits are sorted from most to least significant. Song-Stable and Song-Plastic columns show means. Corrected α indicates the threshold for significance with the Holm-Bonferroni correction. * denotes significantly different groups.

Removed Family	Song-Stable	Song-Plastic	F-Value	Corrected α	p-Value
Muscicapidae	1.8969	2.1768	1.0364	0.05	0.564
Mimidae	1.8969	1.7406	0.7394	0.05	0.608
Passerellidae	1.8818	2.0971	0.4666	0.05	0.669
Icteridae	1.8969	2.1642	0.9848	0.05	0.67
Acrocephalidae	1.9649	2.1804	0.7139	0.05	0.688
Fringillidae	1.9034	2.0971	0.5401	0.05	0.748
Parulidae	1.8829	2.0942	0.5365	0.05	0.76

Table S11: PhylANOVA results for song repertoire when each bird family is omitted. Song traits are sorted from most to least significant. Song-Stable and Song-Plastic columns show means. Corrected α indicates the threshold for significance with the Holm-Bonferroni correction. * denotes traits different groups.

Removed Family	Song-Stable	Song-Plastic	F-Value	Corrected α	p-Value
Acrocephalidae	1.033	3.722	31.0779	0.0083	<0.001*
Fringillidae	1.0383	3.7006	29.3831	0.0083	<0.001*
Mimidae	1.1055	3.3384	22.2449	0.0083	<0.001*
Muscicapidae	1.1055	3.6384	26.0734	0.0083	<0.001*
Passerellidae	1.218	4.0131	26.4254	0.0083	<0.001*
Icteridae	0.9628	4.0616	43.9031	0.0083	0.002*
Parulidae	1.0972	4.0946	37.4843	0.0083	0.002*

Table S12: Brownie results for syllable repertoire when each bird family is omitted. Song traits are sorted from most to least significant. * denotes cases where the two-rate model fit the data significantly better than the one-rate model.

Removed Family	One Rate	Two Rates	p-Value
Passerellidae	-99.3059	-97.9546	0.1
Acrocephalidae	-109.715	-109.2164	0.318
Mimidae	-111.7997	-111.489	0.43
Fringillidae	-110.9574	-110.7158	0.487
Icteridae	-113.4483	-113.2436	0.522
Parulidae	-115.6046	-115.401	0.523
Muscicapidae	-111.2268	-111.0404	0.542

Table S13: Brownie results for interval when each bird family is omitted. Song traits are Song traits are sorted from most to least significant. * denotes cases where the two-rate model fit the data significantly better than the one-rate model.

Removed Family	One Rate	Two Rates	p-Value
Acrocephalidae	-38.372	-31.0345	<0.001*
Icteridae	-43.5138	-38.6556	0.002*
Fringillidae	-44.2357	-39.9282	0.003*
Parulidae	-43.2215	-39.0435	0.004*
Mimidae	-37.7323	-34.1732	0.008*
Passerellidae	-40.7556	-37.7238	0.014*
Muscicapidae	-35.1535	-33.3576	0.058

Table S14: Brownie results for duration when each bird family is omitted. Song traits are Song traits are sorted from most to least significant. * denotes cases where the two-rate model fit the data significantly better than the one-rate model.

Removed Family	One Rate	Two Rates	p-Value
Acrocephalidae	-63.9978	-57.4808	<0.001*
Muscicapidae	-68.4218	-62.5466	<0.001*
Icteridae	-69.2203	-64.2909	0.002*
Parulidae	-67.9632	-63.404	0.003*
Mimidae	-64.2702	-60.1031	0.004*
Passerellidae	-63.0661	-60.5806	0.026*
Fringillidae	-53.9545	-52.6407	0.105

Table S15: Brownie results for syllables per song when each bird family is omitted. Song traits are Song traits are sorted from most to least significant. * denotes cases where the two-rate model fit the data significantly better than the one-rate model.

Removed Family	One Rate	Two Rates	p-Value
Acrocephalidae	-102.1934	-89.5897	<0.001*
Fringillidae	-103.9832	-94.7314	<0.001*
Icteridae	-107.6333	-97.659	<0.001*
Muscicapidae	-106.7467	-95.6359	<0.001*
Parulidae	-105.5031	-96.1153	<0.001*
Passerellidae	-96.8595	-88.2423	<0.001*
Mimidae	-71.5741	-70.2893	0.109

Table S16: Brownie results for song rate when each bird family is omitted. Song traits are Song traits are sorted from most to least significant. * denotes cases where the two-rate model fit the data significantly better than the one-rate model.

Removed Family	One Rate	Two Rates	p-Value
Acrocephalidae	-36.7513	-28.3824	<0.001*
Icteridae	-41.9163	-36.9836	0.002*
Fringillidae	-42.4949	-38.1179	0.003*
Parulidae	-41.5382	-37.2602	0.003*
Muscicapidae	-35.7804	-32.5759	0.011*
Passerellidae	-39.2489	-36.1562	0.013*
Mimidae	-33.6912	-30.9239	0.019*

Table S17: Brownie results for song repertoire when each bird family is omitted. Song traits are Song traits are sorted from most to least significant. * denotes cases where the two-rate model fit the data significantly better than the one-rate model.

Removed Family	One Rate	Two Rates	p-Value
Icteridae	-104.254	-103.5109	0.223
Mimidae	-106.4131	-106.0144	0.372
Fringillidae	-100.0817	-99.7318	0.403
Acrocephalidae	-103.331	-103.0443	0.449
Parulidae	-107.5562	-107.3195	0.491
Muscicapidae	-106.4831	-106.2634	0.507
Passerellidae	-97.4923	-97.3567	0.602

Table S18: Brownie results for syllables per song when each mimid sepcies is omitted. Song traits are Song traits are sorted from most to least significant. * denotes cases where the two-rate model fit the data significantly better than the one-rate model.

Removed Mimid	One Rate	Two Rates	p-Value
Toxostoma rufum	-108.0309	-98.3481	<0.001*
Dumetella carolinensis	-108.1329	-98.3281	<0.001*
Mimus polyglottos	-89.0035	-87.4045	0.074
Mimus gilvus	-82.2923	-82.0747	0.509

Table S19: PhylANOVA results for all song traits when *Melospiza melodia* is labeled Song-Stable. Song-Stable and Song-Plastic columns show means. Song traits are sorted from most to least significant. Corrected α indicates the threshold for significance with the Holm-Bonferroni correction. * denotes traits with significantly different groups.

Song Trait	Song-Stable	Song-Plastic	F-Value	Corrected α	p-Value
Syllable Rep	1.9098	3.9792	41.1603	0.0071	<0.001*
Song Rep	1.094	4.0131	39.4823	0.0083	<0.001*
Syll Song	1.2556	2.2962	9.2658	0.01	0.094
Duration	0.7736	1.2927	2.0783	0.0125	0.42
Continuity	-1.3453	-1.0286	2.1537	0.0167	0.474
Interval	1.6075	1.218	1.3879	0.025	0.567
Song Rate	1.8969	2.0971	0.6079	0.05	0.713

Table S20: Brownie results for song traits when *Melospiza melodia* is labeled Song-Stable. Song traits are sorted from most to least significant. * denotes traits where the two-rate model fit the data significantly better than the one-rate model.

Song Trait	One Rate	Two Rates	p-Value
Syll Song	-110.6482	-100.7673	<0.001*
Interval	-45.2842	-40.5004	0.002*
Duration	-71.2042	-66.3122	0.002*
Song Rep	-113.5829	-113.3919	0.537
Syllable Rep	-120.2983	-120.1567	0.595

Table S21: Adapted from Jenkins 1977 table 2 with new syllable repertoire and syllables per song data included. Syl Rep is the sum of distinct syllables within each song, while Corrected is the number of unique syllables across the full repertoire (note that SR and VPH shared one syllable). The mean of Corrected was used in our paper. Syllables per song was calculated following the definition from our main methods. * marks the individual that was missing from table 2 in the original publication.

Song Type	SR	VPH	CC	PH	KS	DC	ZZ	SE			
Syll/Song	3	2	3	3	2	1	1	4	Syl Rep	Corrected	Syl/Song
_A	1	1							5	4	2.5
Fern	1	1							5	4	2.5
Knob	1	1							5	4	2.5
BR_A			1						3	3	3
_WA			1						3	3	3
A_RB			1						3	3	3
AW_			1						3	3	3
AG_			1						3	3	3
A_RY			1						3	3	3
m YW_A			1						3	3	3
AT_			1	1					6	6	3
_YA				1					3	3	3
GW_A				1					3	3	3
A_RG				1			1		4	4	2
RA_Y				1					3	3	3
YR_AG				1	1				5	5	2.5
AR_					1	1			3	3	1.5
Y_AR					1	1			3	3	1.5
A_WR						1			1	1	1
A_GW						1			1	1	1
_A							1		1	1	1
H. Gully							1	1	5	5	2.5
G_RA							1	1	5	5	2.5
AY_GR							1	1	5	5	2.5
_AY							1	1	5	5	2.5
B_WA								1	4	4	4
Y_AY								1	4	4	4
A_RW*						1			1	1	1
								Mean:	3.5	3.393	2.5

Table S22: To count the syllable repertoire of *Geospiza fortis*, recordings from MaCaulay Library (top section of the table) and sonograms published in Grant and Grant (1996) (bottom section of the table) were examined. The first colmn gives the MaCaulay Library recording ID or the Grant's sonogram ID. The mean number of syllables we counted is at the bottom of the column 3. Because this species has a song repertoire of one, the value for syllables per song was the same as the syllable repertoire.

Geospiza fortis		
ML	Recordist	Syllables
86782	Robert I. Bowman	1
86729	Robert I. Bowman	1
86728	Robert I. Bowman	2
86727	Robert I. Bowman	1
86726	Robert I. Bowman	1
86724	Robert I. Bowman	1
86723	Robert I. Bowman	1
86719	Robert I. Bowman	2
86718	Robert I. Bowman	1
86717	Robert I. Bowman	1
86716	Robert I. Bowman	2
86714	Robert I. Bowman	1
82869	Robert I. Bowman	2
82865	Robert I. Bowman	1
82863	Robert I. Bowman	1
82597	Robert I. Bowman	2
82595	Robert I. Bowman	1
82576	Robert I. Bowman	1
82575	Robert I. Bowman	1
82574	Robert I. Bowman	1
46372	Margery R. Plymire	1

Grant and Grant (1996)	Recordist	Syllables
2666	Grant and Grant	1
4446	Grant and Grant	1
10826	Grant and Grant	1
4339	Grant and Grant	1
5555	Grant and Grant	1
5921	Grant and Grant	1
13901	Grant and Grant	1
17835	Grant and Grant	1
3612	Grant and Grant	1
16805	Grant and Grant	1
17103	Grant and Grant	1
5505	Grant and Grant	1
17796	Grant and Grant	1
5578	Grant and Grant	1
15236	Grant and Grant	1
15359	Grant and Grant	1

14963	Grant and Grant	1
10228	Grant and Grant	1
4946	Grant and Grant	1
4913	Grant and Grant	2
714	Grant and Grant	1
16167	Grant and Grant	2
5110	Grant and Grant	2
14687	Grant and Grant	1
5275	Grant and Grant	1
15514	Grant and Grant	1
A	Grant and Grant	1
10081	Grant and Grant	1
2639	Grant and Grant	1
10211	Grant and Grant	1
10550	Grant and Grant	2
14720	Grant and Grant	1
4620	Grant and Grant	2
B	Grant and Grant	2
	Mean:	1.20

Table S23: To count the syllable repertoire of *Geospiza scadens*, recordings from MaCaulay Library were examined. The first colmn gives the MaCaulay Library recording ID. The mean number of syllables we counted is at the bottom of the column 3. Because this species has a song repertoire of one, the value for syllables per song was the same as the syllable repertoire.

Geospiza scandens		
ML	Recordist	Syllables
133749351	Eric DeFonso	1
46235	Robert I. Bowman	1
46234	Robert I. Bowman	2
46233	Robert I. Bowman	2
46228	Robert I. Bowman	2
46224	Robert I. Bowman	2
46222	Robert I. Bowman	2
46220	Robert I. Bowman	1
49219	Robert I. Bowman	2
46218	Robert I. Bowman	1
46217	Robert I. Bowman	1
	Mean:	1.54
